# The NRF2 transcription factor plays a dual role in colorectal cancer: A systematic review

**DOI:** 10.1371/journal.pone.0177549

**Published:** 2017-05-18

**Authors:** C. Gonzalez-Donquiles, J. Alonso-Molero, T. Fernandez-Villa, L. Vilorio-Marqués, A. J. Molina, V. Martín

**Affiliations:** 1 CIBER Epidemiología y Salud Pública (CIBERESP), Madrid, Spain; 2 Gene-Environment and Health Research Group, University of Leon, León, Spain; Fox Chase Cancer Center, UNITED STATES

## Abstract

**Background:**

Colorectal cancer is one of the most common cancers worldwide, and is influenced by the interplay of various factors, including a very strong genetic component. For instance, incorrect mitochondrial biogenesis is correlated with increased risk of developing colorectal cancer. Thus, it is important to understand the consequences of changes in both the expression and the correct function of the transcription factors that regulate mitochondrial biogenesis, namely NRF2.

**Objectives:**

The main objective of this paper is to characterise the relationship between NRF2 and colorectal cancer by compiling data from an exhaustive literature search.

**Methods:**

Information was obtained by defining specific search terms and searching in several databases. After a strict selection procedure, data were tabulated and the relationships between articles were assessed by measuring heterogeneity and by constructing conceptual maps.

**Results and discussion:**

We found a general consensus in the literature that the presence of oxidizing agents as well as the inhibition of the NRF2 repressor Keap1 maintain NRF2 expression at basal levels. This predominantly exerts a cytoprotective effect on cells and decreases risk of colorectal cancer. However, if NRF2 is inhibited, protection against external agents disappears and risk of colorectal cancer increases. Interestingly, colorectal cancer risk is also increased when NRF2 becomes overexpressed. In this case, the increased risk arises from NRF2-induced inflammation and resistance to chemotherapy.

**Conclusion:**

The proper basal function of NRF2 and Keap1 are essential for preventing oncogenic processes in the colon. Consequently, any disruption to the expression of these genes can promote the genesis and progression of colon cancer.

## Introduction

Colorectal cancer (CRC) is one of the most common cancers worldwide, and causes more than half a million deaths per year [1]. This type of cancer is a useful model for studying both carcinogenesis and tumour progression, as colonocytes follow a systematic process of proliferation, differentiation and transformation (adenoma to a carcinoma) [2]. CRC arises from stem cells in the colon [3], and its development is partly due to functional changes in the mitochondrial genome (mtDNA) [4].

Mitochondria play a crucial role in physiological processes, and their deregulation contributes to the genesis and progression of CRC. This is known as the Warburg effect, which consists of metabolic reprogramming that is characteristic of tumour processes. Thus, it is important that all genes involved in mitochondrial biogenesis are expressed according to a standard pattern. This process is highly coordinated and depends on the activity of several key proteins, including Nuclear Respiratory Factor 1 (NRF1) and Nuclear Respiratory Factor 2 (NRF2), which regulate the expression of respiratory chain proteins, and act as anti-oxidants [5].

NRF transcription factors control the functionality of the respiratory chain, the main pathway responsible for producing reactive oxygen species (ROS). ROS are extremely harmful for cellular micromolecules because they induce tumorigenesis through non-specific reactions with nucleic acids, proteins, and lipids [6].

**Fig 1:**
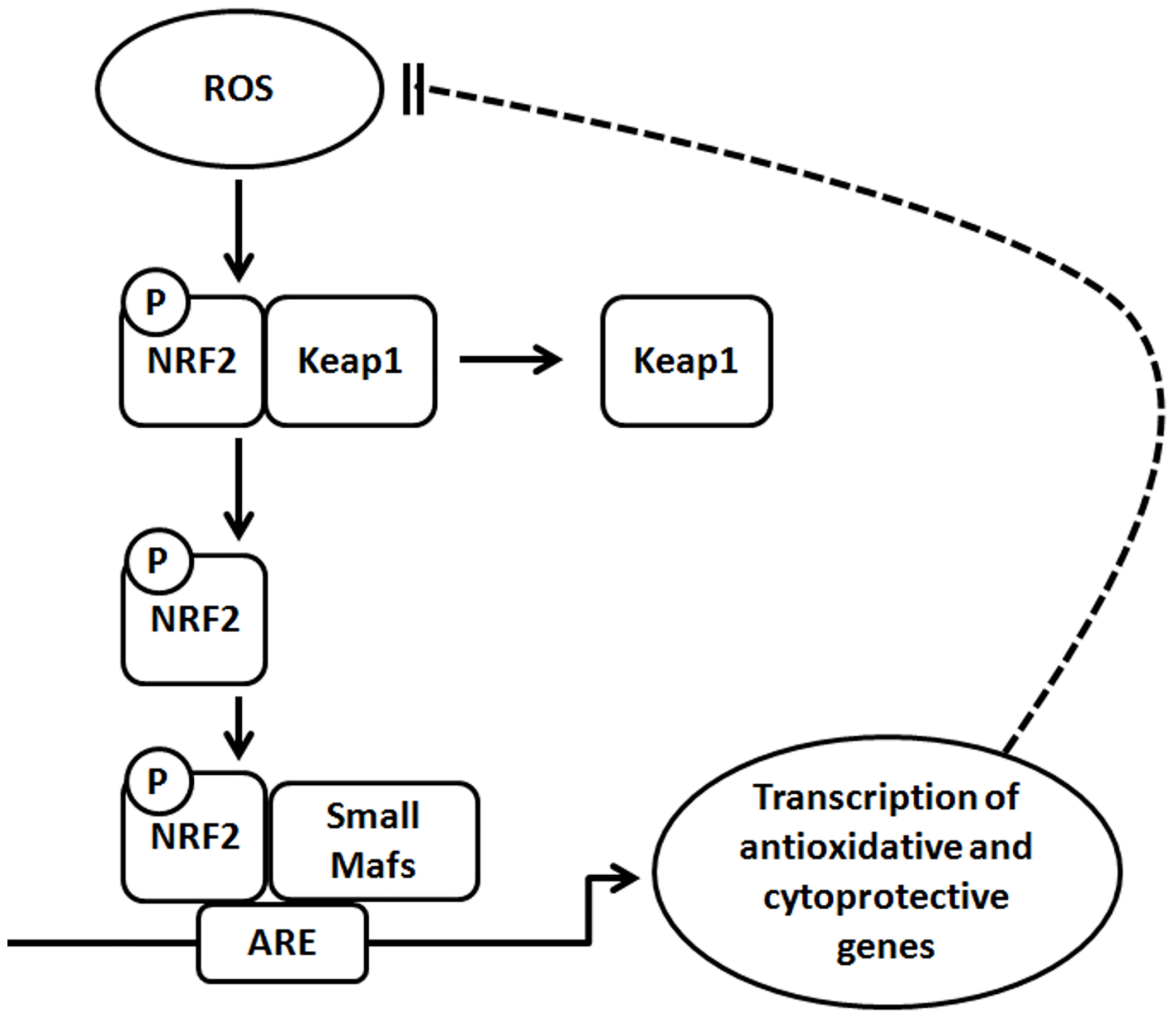
Simplified diagram of the activation of NRF2 transcription factor via ROS. When ROS is acumulated, NRF2 is released from Keap1 and heterodimerizes with Small Mafs. In this way, it binds to AREs and the transcription of antioxidant and cytoprotective genes occurs.

NRF2 is considered to be a main defence mechanism and regulator of cell survival [7]. Under healthy conditions, NRF2 protects against tumorigenesis and cancer progression by attenuating genotoxic compounds that emerge both intrinsically and extrinsically. However, activation of the NRF2 defence response can promote the survival of both normal and cancer cells by creating an optimal environment for cell growth. Furthermore, NRF2 protects tumour cells from oxidative stress, chemotherapeutic agents, and radiotherapy, promoting tumour genesis and progression, and also metabolic reprogramming to anabolic pathways [8]. Fig 1 shows a simplified diagram of the mechanisms that can activate the transcription factor NRF2. NRF2 is sequestered by the inhibitor Keap1 and is transcriptionally inactive until Keap1 is dissociated by ROS. This dissociation allows NRF2 to form transcriptionally active complexes with other proteins, such as Mafs, and, after the transcription of cytoprotective and antioxidative genes, to remove ROS [9].

In this study, we provide a qualitative systematic review to summarize the role of the NRF2 transcription factor. Our goal was to assess the effects of differential expression of NRF2 on CRC.

## Methods

### Literature search strategy

Literature searches and quality assessment were performed according to 'Guidance on the Conduct of Narrative Synthesis in Systematic Reviews' (S1 File) [10]. The search results were independently assessed by two individuals to minimize bias (one was an expert on this topic and the other had no knowledge of it).

### Design of search strategies and use of Boolean operators

For each search, synonyms, related terms and spelling variations were taken into account, and Boolean operators were used to relate the terms. Searches were performed on eight different databases (PubMed, Scopus, Web of Science (WOS), Cochrane library, PysCinfo, PLoS One, Scielo and PubMedCentral (PMC)) using the following terms:

'Colorectal OR Colon OR Rectum OR Rectal' AND 'Cancer OR Carcinoma OR Tumor OR Tumour OR Neoplasm OR Cancer Cells' AND 'Mitochondrial Biogenesis OR Mitochondrial dysfunction OR Mitochondria OR Mitochondrion' AND 'NRF2 OR NFE2L2 OR Nuclear Respiratory Factor 2 OR Nuclear Factor Erythroid 2 Like 2'.
'Colorectal OR Colon OR Rectum OR Rectal' AND 'Cancer OR Carcinoma OR Tumor OR Tumour OR Neoplasm OR Cancer Cells' AND 'Warburg effect' AND 'NRF2 OR NFE2L2 OR Nuclear Respiratory Factor 2 OR Nuclear Factor Erythroid 2 Like 2'.
'Colorectal OR Colon OR Rectum OR Rectal' AND 'Cancer OR Carcinoma OR Tumor OR Tumour OR Neoplasm OR Cancer Cells' AND 'Oxidative phosphorylation OR OXPHOS OR Anaerobic glycolysis' AND 'NRF2 OR NFE2L2 OR Nuclear Respiratory Factor 2 OR Nuclear Factor Erythroid 2 Like 2'.

To retrieve optimal results when using the Scopus database, it was necessary to modify the search criteria. Thus, only the following more specific terms were used:

'CRC' AND 'NRF2 OR NFE2L2' AND 'Warburg'
'CRC' AND 'NRF2 OR NFE2L2' AND 'OXPHOS'.

We included only scientific reports written in English, and published after 2006, to obtain a current review of this issue. We collected articles using all combinations of search terms.

### Selection process

The articles returned by the searches were manually screened to identify relevant articles to be included in this review: **1**. Search results were integrated using informatics programs. We used the EndNote reference manager to remove duplicate articles. **2**. Titles and abstracts were assessed to remove irrelevant articles. Only articles satisfying the following criteria were included: (i) the article considers CRC or other related diseases; (ii) the title of the article includes the word NRF2, or the name of another related gene; (iii) the article discusses the relationship between CRC and either mitochondrial biogenesis, OXPHOS or the Warburg effect. **3**. The full texts of potentially relevant articles were retrieved and analysed to verify that they met the inclusion criteria.

**Fig 2:**
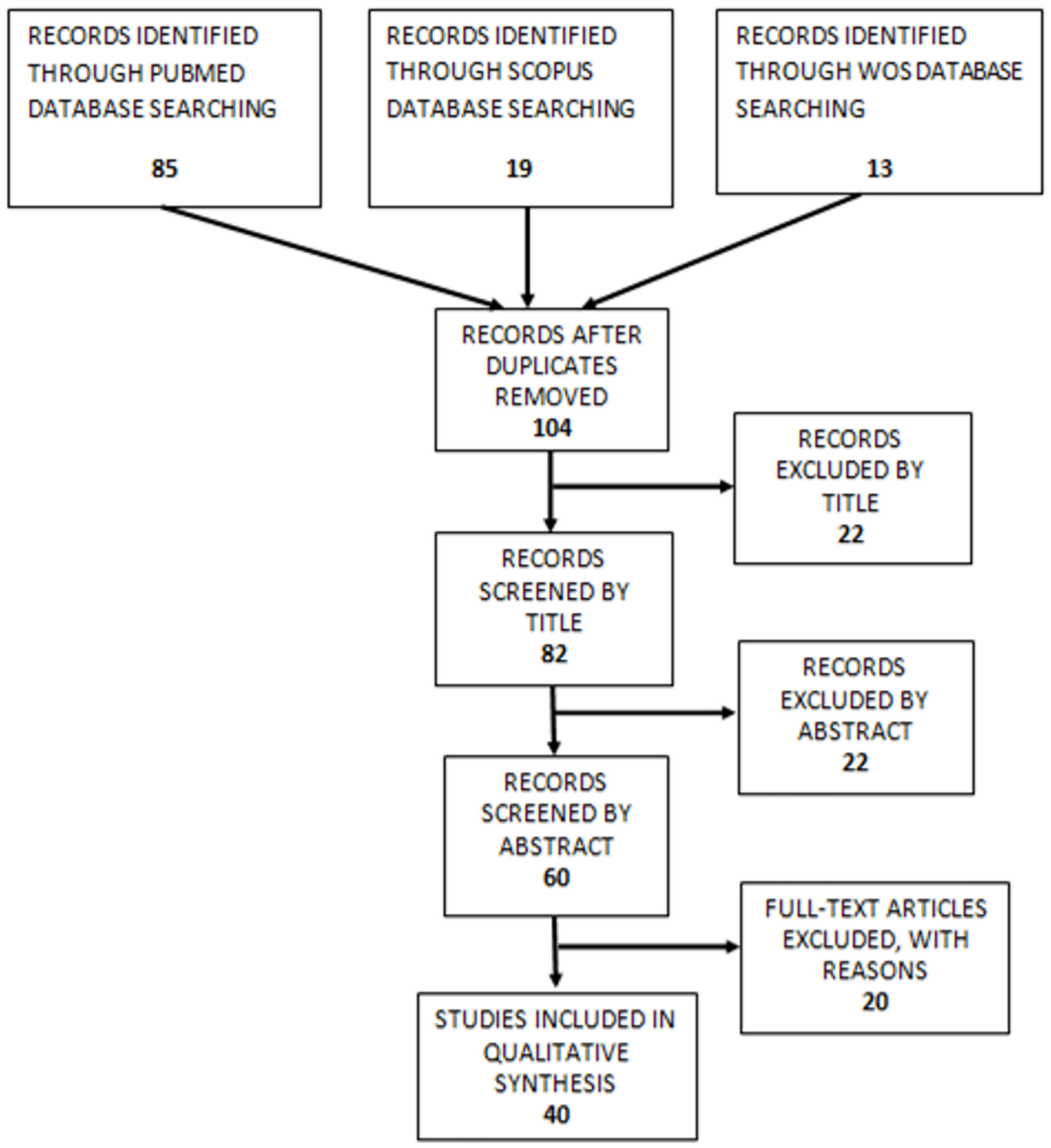
PRISMA 2009 flow diagram. This flow chart shows the process of selecting articles included in the qualitative systematic review.

Fig 2 shows a flow chart summarising each step in the search process. Reports that did not meet the inclusion criteria were excluded from the analysis. S1 Table lists the articles excluded in the last step of the search process.

### Assessment of results

#### Tabulation

We tabulated the following key data from each article: first author, publication year, organism/cell type, primary results and conclusion (S2 Table).

#### Heterogeneity

**Fig 3:**
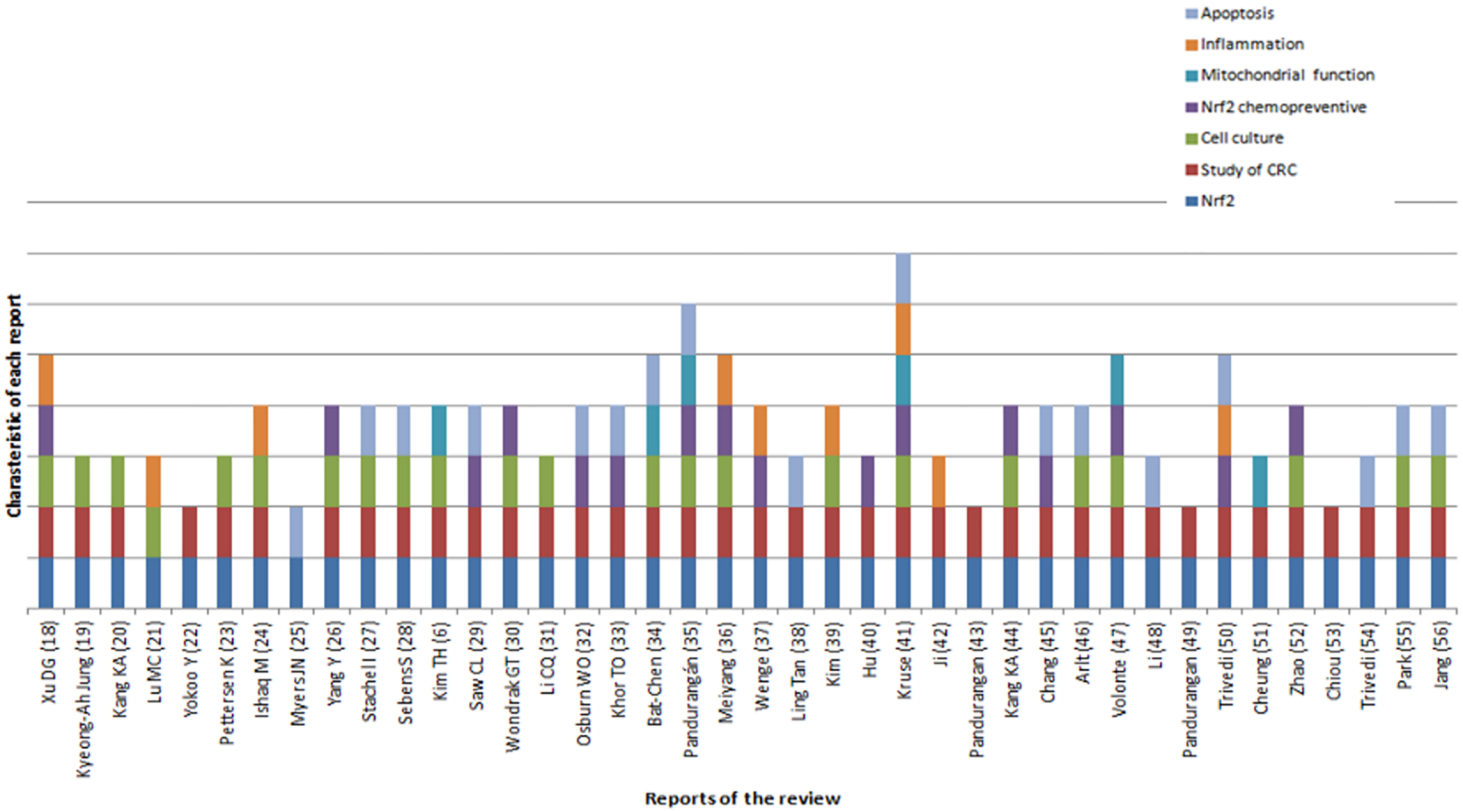
Heterogeneity assessment. This graph evaluates if certain features are present in the articles included in this review.

To assess the heterogeneity of the selected articles, we analysed the following aspects: expression of NRF2, relationship to CRC, use of cell culture, NRF2 as a chemotherapeutic agent, apoptosis, the inflammatory process and mitochondrial function. To more easily view the specific characteristics of each article, and to evaluate the homogeneity among them, we represented each article in a stacked bar chart (Fig 3).

#### Concept map

We constructed three maps to visualize the links between the selected articles. We aimed to relate three key concepts behind NRF2 expression and its role in the development of CRC so that we could draw conclusions and suggest ideas for future research.

## Results

### Article selection

The search described above identified relevant articles only in the Pubmed, Web of Science, and Scopus databases. Removing duplicated articles from these search results resulted in a total of 104 articles. We reviewed the titles of these articles and discarded those not related to our topic, reducing the number of articles to 82. We then reviewed the abstracts, further reducing the number of articles to 60. Finally, we reviewed the full text, and selected 40 articles for the review. This article selection process is summarized in Fig 2. Articles excluded in the last step are summarized in Supplementary Information 1.

### Tabulation

Complete data from the 40 articles selected for this review are shown in Supplementary Information 2. All selected papers were published after 2006. The geographic settings of the studies vary, with nine from in the USA, nine from China, seven from Korea, four from Germany, four from Malaysia, three from India, two from Japan, and one each from the United Kingdom, Norway, and Australia. Most studies were based on human cancer cell lines. Only 13 studies used animal experimentation (mice), and four considered patients with ulcerative colitis or Crohn's disease. The most important results from the selected articles are shown in Supplementary Information 2. Titles, author and publication date of the included articles are shown in Table 1.

**Table 1:** Summarized table about the included reports.

First author	Primary results
Kim et al., 2011 [6]	Hypoxia-induced angiogenesis can be blocked by inhibiting Nrf2. Inhibition of Nrf2 reduced tumour growth and decreased angiogenesis in mice xenografts, and was associated with a lower accumulation of HIF-1 α under hypoxic conditions. Hypoxia cannot activate HIF-1α in Nrf2-inhibited colon cancer cells. PGC1-α and NRF1 levels were unaffected by inhibition of Nrf2. However, destabilization of HIF-1α is associated with a weakened mitochondrial function in colon cancer cells.
Bat-Chen et al., 2010 [11]	Allicin induces apoptosis in colon cancer cells and stimulates Nrf2 nuclear accumulation.
Pandurangan et al., 2014 [12]	Nrf2 is activated by luteolin
Kim et al., 2013 [13]	Catechol residues are essential for activating Nrf2.
Pandurangan et al., 2015 [14]	Cocoa actives Nrf2 expression.
Li et al., 2016 [15]	Nrf2 is activated by luteolin.
Chiou et al., 2011 [16]	PS and RS promote Nrf2 activation and reduce colon tumorigenesis.
Trivedi et al., 2013 [17]	Lipoic acid (LA) increases Nrf2 and HO-1 expression in the colon. LA protects against DNA damage in the colon.
Pettersen et al.,2016[18]	In SW620 cells, DHA induces an increase in ROS and causes the nuclear import of Nrf2. An increase in the level of Nrf2 was detected in the nucleus of CaCo2 and SW620 cells.
Myers et al., 2014[19]	Nrf2 levels are lower in inflamed tissue of patients with UC and Crohn´s disease. Nrf2 protein levels were twice as high in patients with diverticulitis than in patients with UC-associated cancer.
Xi et al., 2013 [20]	Nrf2 has an inhibitory effect on the development of the CRC. Nrf2 diminishes the development of dysplasia. Nrf2 is overexpressed in tumour tissues.
Park et al., 2010 [21]	Rottlerin induces Nrf2 nuclear translocation.
Jang et al., 2016 [22]	Simvastatin induces Nrf2 expression and its nuclear translocation. Simvastatin induces Nrf2-related antioxidant expression through the ERK and PI3K/Akt pathways.
Trivedi et al., 2016 [23]	MEL increases the expression of Nrf2 and other target genes.
Xu et al., 2015[24]	PYDDT promotes ROS production, increases Nrf2 expression and decreases the CRC risk due to a reduction in genotoxicity and an increment of apoptosis.
Tan et al., 2015 [25]	Brewer's rice promotes Nrf2 activation.
Volonte et al., 2013 [26]	Calveolin inhibits Nrf2 translocation to the nucleus, thereby promoting premature senescence of CRC cells.
Kruse et al., 2016 [27]	IMC-co-cultured NCM460 or Colo320 cancer cells were less sensitive to TRAIL/etoposide-induced apoptosis due to Nrf2-induced proteasome activity. Immunostaining of IBD tissues confirmed Nrf2 activation within inflamed areas of the colonic epithelium, and greater proteasome protein expression.
Yang et al., 2014[28]	Low concentrations of digitoflavonoids are potential Nrf2/Are activators in colon tumour, liver and kidney cells. Digitoflavonoids stimulate the expression of antioxidant defence proteins, the expression of Nrf2, and its translocation to the nucleus. After treatment with digitoflavonoids, AKT, ERK1/2 and p38 AMPK phosphorylation increases. Inhibition of AKT and ERK1/2 phosphorylation does not influence Nrf2 activation. p38 phosphorylation however is essential for Nrf2 activation. Mice treated with digitoflavonoids exhibited a decrease in the number and size of tumours and crypts as a result of Nrf2 (and its target genes) activation.
Pandurangan et al., 2014 [29]	Nrf2 transactivation and translocation.
Wondrak et al., 2010 [30]	Cinnamaldehyde upregulates Nrf2 and its downstream target genes. Nrf2 provides protection against oxidative stress-induced genotoxicity.
Li et al., 2009 [31]	Exposing HTC116 cells to NO results in the nuclear translocation of Nrf2 as well as its transcriptional upregulation.
Jung et al., 2013 [32]	Silenced Keap1 activates Nrf2 signalling in colon cancer cell lines. Silencing Keap1 can upregulate the expression of AKRs and attenuate oxidative damage caused by stress.
Lu et al., 2016 [33]	CPUY192018 provides cytoprotection against oxidative damage caused by DSS in NCM460 cells. Pretreatment with CPUY192018 increases the survival rate by inhibiting apoptosis and by impeding the DSS-induced arrest of the S phase of the cell cycle. CPUY192018 induces an increase in Nrf2 protein levels in colonic NCM460 cells and its accumulation in the nucleus.
Stachel et al., 2014 [34]	IER3 overexpression inhibits Nrf2 activation. The absence of IER3 causes a reduction in ROS levels as a result of an increase in Nrf2 activity.
Sebens et al., 2011 [35]	Nrf2 is activated when cells are exposed to inflammatory macrophages. This increases proteosomal activity and the expression of proteosomal genes in a Nrf2-dependent manner.
Yokoo et al., 2016[36]	In Nrf2^-/-^ mice treated with KBrO3, atypical hyperplasia, adenoma and adenocarcinoma were all observed in the upper small intestine. These same mice displayed an increase in the number of aberrant crypts compared with their Nrf2^+/+^ counterpart.
Kang et al., 2014 [37]	Nrf2 promotes resistance to 5-FU treatment in CRC cells.
Zhao et al., 2015 [38]	Nrf2 expression is higher in CRC cells that are resistant to 5-FU treatment.
Hu et al., 2013 [39]	Nrf2 is higher expressed in tumour tissue than in normal tissue. Nrf2 overexpression is related to larger tumours with advanced stage and metastasis. Directional metastases may be associated with activation of Nrf2.
Ji et al., 2014 [40]	Nrf2 expression is higher in CRC tissue, and is positively correlated with Duke's stage and clinical prognosis.
Kang et al., 2016 [41]	Nrf2 expression is higher in those colon cells resistant to 5-FU. Demethylation upregulates Nrf2 in 5-FU resistant SNUC5 cells.
Ishaq et al., 2014[42]	Nrf2 is involved in the protective response of HT29 cancer cells. It induces apoptosis through activating caspase 3/7.
Arlt et al., 2009 [43]	There is a high activity of Nrf2 in the nucleus of CRC cells. Nrf2 overexpression increases proteasomal activity.
Li et al., 2008 [44]	Compared with WT mice, Nrf2 KO mice suffer from more severe ulcerative colitis, loss of crypts, an infiltration of inflammatory cells, and rectal bleeding.
Khor et al., 2006 [45]	Compared with WT mice, Nrf2 KO mice had a smaller average colon length. After exposure to DSS, Nrf2 KO mice showed a loss in crypts with severe inflammation.
Osburn et al., 2007 [46]	DSS exposure in Nrf2-deficient mice led to the formation of multiple aberrant crypts. In addition, treatment with AOM promoted colitis-associated tumorigenesis (due to inflammation of the colon).
Saw et al., 2011 [47]	Nrf2 KO mice suffer more severe colitis than WT mice after treatment with DSS. After adding AOM, Nrf2 KO mice have a greater tendency to develop CRC and dysplasia, and display increased prolapse and rectal bleeding. Nrf2 is required for protection against inflammation-associated CRC. Nrf2 KO mice have a greater susceptibility of developing aberrant crypts associated with inflammation.
Cheung et al., 2014 [48]	Nrf2 KO mice have an increased number and size of polyps and cell proliferation.
Chang et al., 2013 [49]	Proteins of the Nrf2 pathway are actively expressed in CRC.

### Heterogeneity

The characteristics of each report are shown in Fig 3.

### Building a concept map

**Fig 4:**
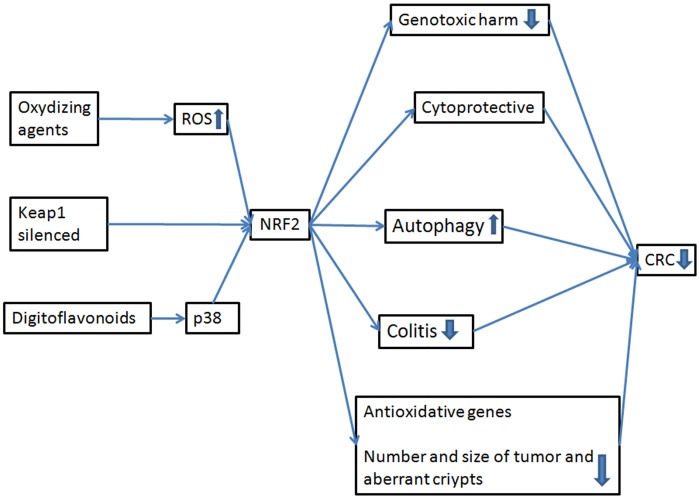
Standard expression of NRF2 and the relation with CRC. Oxidizing agents, the silencing of the Keap1 or the digitoflavonoids induce the expression of NRF2, decreasing the risk of CRC.

**Fig 5:**
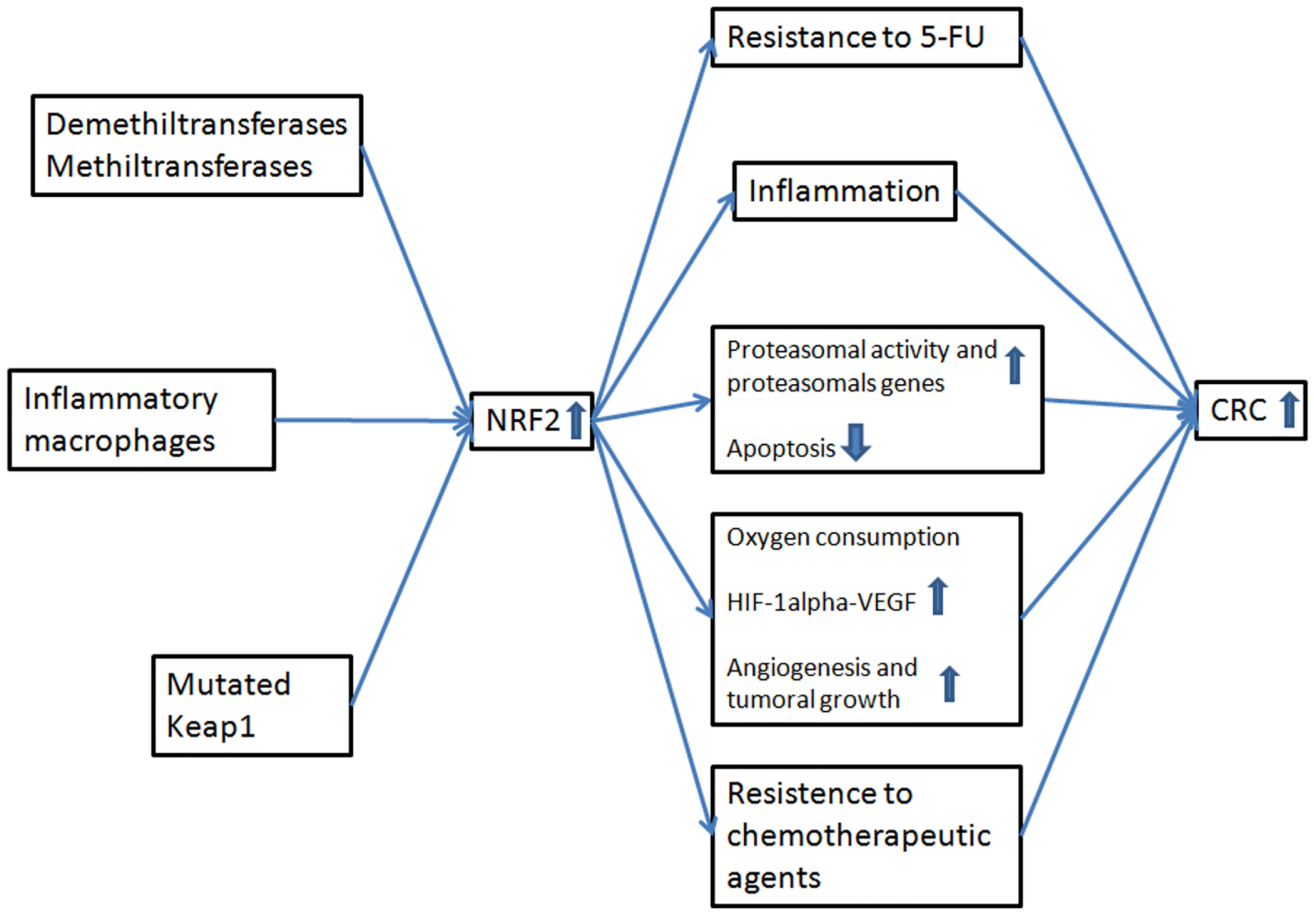
Overexpression of NRF2 and the relation with CRC. Constitutive activation of NRF2 promotes a serie of events that lead to an increased risk of CRC.

**Fig 6:**
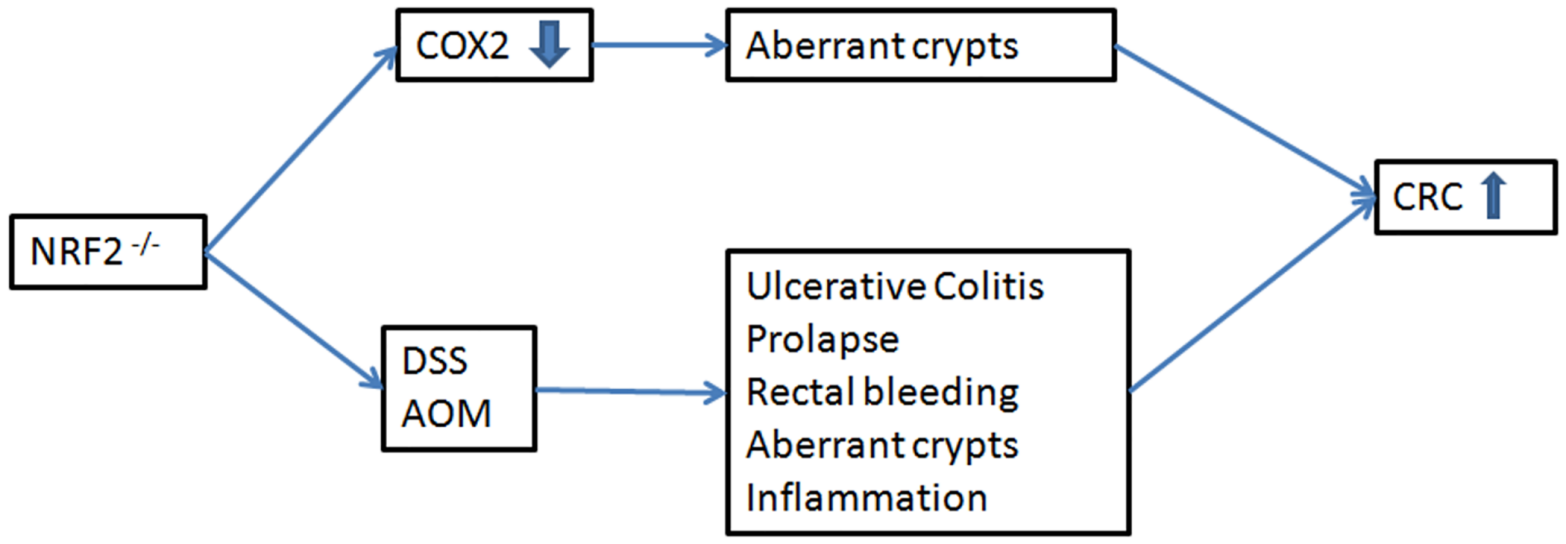
Inhibition of NRF2 and the relation with CRC. Inhibition or permanently silencing of NRF2 induce the formation of aberrant crypts and other typical processes related to the increased risk of CRC such as rectal bleeding or ulcerative colitis.

To interconnect the ideas gathered from the articles included in this review, we constructed three conceptual maps focused on the expression of NRF2 and its effect on colon cancer. The first conceptual map was constructed on the basis of standard NRF2 gene expression and how it is influenced by various mechanisms or external agents. The second conceptual map explains the various effects and mechanisms of NRF2 overexpression, and the third shows the effects of NRF2 silencing or inhibition. Analysing all three conceptual maps sheds light onto how NRF2 expression affects CRC. As shown in Fig 4, correct expression of NRF2 prevent CRC in humans, while altered expression of this gene promotes tumour genesis and progression (Figs 5 and 6).

## Discussion

The articles selected show that research on the role of NRF2 in CRC can be classified into three broad groups: i) those that analyse standard expression and observe a cytoprotective effect on CRC [11–17], and those that analyse variations in expression, either ii) overexpression or iii) inhibition, both leading to increased CRC risk. Below, we discuss the findings in each of these three lines of research.

The first concept map (Fig 4) shows the protective effect of induced expression of the NRF2 gene on CRC following a moderate accumulation of ROS due to specific external agents [18–22]. When NRF2 activates, it exerts a cytoprotective effect by decreasing genotoxic damage and promoting autophagy [23]. Sometimes, NRF2 expression leads to reduced cell proliferation and increased apoptosis of colon cancer cells [24–27]. In all cases, the authors observed the same final result, namely reduced risk of CRC. Another example of this behaviour was reported by Yang et al. (2014), in which digitoflavonoids were found to act upon p38 and to induce NRF2 target genes and antioxidants. They reported a reduction in both the number and size of tumours, as well as a decrease in the number of aberrant crypts, further pointing to a protective effect of NRF2 on CRC [28]. Therefore, expression of NRF2 and its target genes, such as glutathione synthase, likely play a crucial role in cytoprotective mechanisms against CRC [29–31].

In the studies mentioned up to this point, NRF2 expression is directly induced by the external agents. However, NRF2 expression can also be promoted indirectly by other mechanisms, via the repressor Keap1. For example, Jung and Kwak, 2013 silenced Keap1 and found that induction of NRF2 promoted the expression of AKR members, which are involved in the detoxification process, leading to reduced risk of CRC [32]. In another study, Lu et al., 2016 used an inhibitor of its interaction with NRF2, CPUY192018, and observed that activated NRF2 acted as a protective factor upon exposure to DSS, decreasing CRC risk [33].

In all of these cases, standard NRF2 expression levels function as a protective factor against CRC by providing cytoprotection and by activating antioxidant target genes. These results are corroborated by the review by Pandurangan and Esa, (2014) and Pandurangan et al (2014a, 2014b), who also support the idea that expression of NRF2 decreases the risk of CRC [12, 29, 50].

As shown in the second concept map (Fig 5), several studies have reported that NRF2 overexpression increases risk of CRC. This overexpression can occur for a number of reasons, including constitutive mutations in the Keap1 repressor gene or in the NRF2 gene itself. Stachel et al. (2014) found that excessive levels of ROS induced overexpression of NRF2, leading to inflammation of the colon tissue and promoting tumorigenesis [34]. In another study, Sebens et al. (2011) reported overexpression of NRF2 in tissues exposed to inflammatory macrophages, and a simultaneous increase in the activity of proteosomal genes, leading to reduced apoptosis and uncontrolled proliferation. This in turn results in greater risk of CRC [35]. Other authors have observed similar results when NRF2 is overexpressed as a consequence of silencing, or as a result of epigenetic changes in this gene [36–38, 51]. Another way to induce overexpression of NRF2 is using chemical compounds. Kim et al. (2011) found that T-BHQ-induced overexpression of NRF2 resulted in increased O_2_ consumption, which is correlated with higher HIF-1α and VEGF signalling, which in turn directly increases angiogenesis and tumour growth [6]. Therefore, as mentioned above, overexpression of the NRF2 gene is closely linked to increased risk of CRC [39, 40]. In this case, CRC risk can increase by promoting colonic inflammation, decreasing apoptosis, or promoting angiogenesis and uncontrolled cell proliferation.

Notably, NRF2 overexpression has also been linked to increased resistance to the chemotherapeutic agent 5-FU via the activity of demethylases and methyltransferases. In addition to the increase risk of CRC, this implies a poorer response to treatments based on this agent. [37,41].

The third concept map (Fig 6) shows that inhibited expression of NRF2 also has consequences for this type of cancer, since one of NRF2’s functions is to protect against damaging and carcinogenic substances [42,43]. Various studies highlight the role of NRF2 on CRC via its inhibition. Li et al. (2008) related Nrf2 inhibition with increased risk of CRC [44]. Yokoo et al. (2016) found that silencing Nrf2 resulted in decreased expression of the COX2 gene, leading to an increase in the number of aberrant crypts, resulting in the formation of adenoma, adenocarcinoma and ultimately CRC [36]. Other studies have compared the effects of silencing the Nrf2 gene in mice and exposing them to treatments that induce ulcerative colitis (DSS) or colon carcinogenesis (AOM) [45–46]. Nrf2 knockout mice with ulcerative colitis suffer prolapse, rectal bleeding, inflammation, and as in the previous case, show an increase in the number of aberrant crypts. Together, these symptoms contribute to the development of CRC. This information is corroborated by reviews from Saw and Kong in 2011 [47] and Cheung et al. in 2014 [48].

Analysing the behaviour of this gene, not at a single time point, but throughout the various stages, Chang et al. (2013) suggested that the differential expression profile of NRF2 in tumour versus normal tissue could be an interesting new target for CRC treatment [49]. Similarly, Arlt et al. (2009) observed that NRF2 expression is beneficial during early stages, but can contribute to tumorigenesis in the colon at later stages [43]. These data are also supported by Menegon et al (2016) and Sporn M and Liby K (2012), who described NRF2 not only as an oncogene but also as a tumour suppressor gene [52,53]. In addition, few studies have analysed the role of Single Nucleotide Polymorphisms (SNPs) in this gene and its promotor, although Yokoo et al. (2016) suggested that some SNP alleles in the NRF2 gene could increase CRC risk based on previous studies of these polymorphisms and the risk of ulcerative colitis [36].

Finally, another important factor is the type of cell used in the studies included in this review. Most of the articles focus on colon stem cells, which need more energy and therefore require greater expression of NRF2 to obtain the necessary ATP for proliferation, migration and differentiation, such that altered expression of NRF2 increases genetic instability leading to increased CRC risk. *In vivo*, these cells migrate to the apical part of the crypt and become mature epithelial cells, and altered expression of NRF2 during migration and differentiation, will lead to an accumulation of mutations that promote tumorigenesis due to absence of cytoprotection. Thus, NRF2 function is probably essential in both the stem cells of the colon and in mature epithelial cells [54].

Although we searched several databases using an exhaustive list of relevant terms, our study has some limitations. We only selected articles published in English, which could result in selection bias. In addition, the use as search terms such as oxidative phosphorylation, mitochondria or Warburg effect, could limit the selection of articles that relate NRF2 to colorectal cancer, although searches performed without these concepts in several databases did not return any additional relevant articles.

In conclusion, it is clear that the NRF2 transcription factor has a very complex role in the cell, and that its expression can be strongly affected by external agents. Any disruption of its standard expression, be it overexpression or silencing, can promote the genesis and progression of CRC. Further studies are needed to clarify the role of NRF2, particularly to analyse the role of various SNPs that could generate variability in the expression and function of the protein in humans, as well as to study the pathways regulated by this gene, and possible interactions with environmental and behavioural factors, although such studies require large sample sizes.

## Supporting information

S1 File Guidance on the Conduct of Narrative Synthesis in Systematic Reviews. (PDF)

S2 File Check list. (DOCX)

S1 Table Excluded reports which not keep the inclusion criteria. (DOCX)

S2 Table Tabulation of included reports. (DOCX)
