# Supplementary material for: The NRF2 transcription factor plays a dual role in colorectal cancer: A systematic review
Source: PLoS One. 2017 May 18;12(5):e0177549. doi: 10.1371/journal.pone.0177549 (PMC5436741; doi:10.1371/journal.pone.0177549)
Supplement: S1 File — (PDF) [file pone.0177549.s001.pdf]

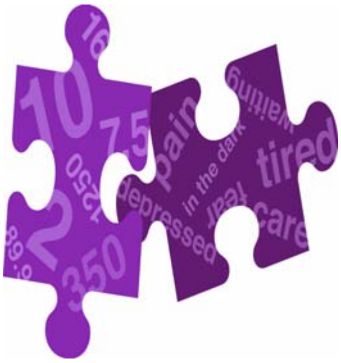

Guidance on the Conduct of Narrative Synthesis in Systematic Reviews

A Product from the ESRC Methods Programme

Jennie Popay[1]
Helen Roberts[2]
Amanda Sowden[3]
Mark Petticrew[4]
Lisa Arai[2]
Mark Rodgers[3]
Nicky Britten[5]
with
Katrina Roen and Steven Duffy

[1]Institute for Health Research, Lancaster University

[2]Child Health Research and Policy Unit, City University

[3]Centre for Reviews and Dissemination, University of York

[4]MRC Social and Public Health Sciences Unit, University of Glasgow: Mark Petticrew is funded by the Chief Scientist Office of the Scottish Executive Department of Health

[5]Peninsula Medical School, Universities of Exeter and Plymouth

Version 1: April 2006

TABLE OF CONTENTS

	Page
Acknowledgements…………………………………………………………………………..	4
Chapter 1 Introduction and the Purpose of the Guidance……………………………….	5
Chapter 2: The main elements in a systematic review process.................................	9
Chapter 3: Guidance on narrative synthesis – an overview……………………………..	11
Chapter 4: Applying the guidance 1: effectiveness studies……………………………..	25
Chapter 5: Applying the guidance 2: implementation studies…………………………..	51
Chapter 6: Implications for future research……………………………………………..	67
References……………………………………………………………………………....	69
Appendix 1: Methods used in the production of the guidance………………………....	73
Appendix 2: Bibliography of methodological texts used in the production of the guidance………………………………………………………………………………………	87

ACKNOWLEDGEMENTS

The research reported here was funded by the ESRC (Grant reference number H33250019) within the ESRC Methods Programme. There are many people who have contributed to this work along the way to whom we would like to extend our thanks. We are grateful to Professor Angela Dale, the Director of the programme for her support throughout the project. We are also grateful to the international panel of experts in research synthesis including in particular: Professor David DuBois, University of Illinois; Dr Jeanne Daly, Mother and Child Health Research, La Trobe University; Professor Mike Fisher, Social Care Institute for Excellence; Angela Harden, The EPPI Centre, Institute of Education, London; Professor Cindy Mulrow, University of Texas; Dr Pierre Pluye, McGill University; Professor Helen Thomas, McMaster University; Dr Carl Thompson, University of York; Dr Heather McIntosh, NHS Quality Improvement Scotland; and Ros Collins, Dr Catriona McDaid and Dr Nerys Woolacott at CRD University of York. The extensive and invaluable comments from panel members helped us to improve on earlier versions of this guidance but the responsibility for the final product is, of course, entirely our own. Finally, we would like to thank the individuals in our various administrative teams and research support offices who have supported the work in many ways. Thanks are due in particular to Erja Nikander, Yvonne Moorhouse, and Vicki Bell in Lancaster and Mel Bryan at City who helped at various points with producing documents, travel arrangements, organising meetings, managing budgets and to Lisa Stirk at CRD in York and Vicki Bell at Lancaster who developed and maintain our website.

We would also like to pay tribute to Professor Sally Baldwin who died as a result of an accident in October 2003. Sally developed the proposal for this work with us. There have been many tributes to her extraordinary qualities as a researcher, teacher, colleague and friend. We will not repeat these here – suffice it to say that her stimulating and good humoured contributions to our intellectual endeavours have been badly missed.

CHAPTER 1: ABOUT THE GUIDANCE

Do domestic smoke alarms save lives? Can young offenders be 'scared straight' through tough penal measures? What factors should be considered when designing and implementing a multi-sectoral injury prevention programme in a local area? Making sense of large bodies of evidence drawn from research using a range of methods is a challenge. Ensuring that the product of this synthesis process can be trusted is important for policy makers, for practitioners and for the people research is intended to benefit. There are a number of ways in which research evidence can be brought together to give an overall picture of current knowledge that can be used to inform policy and practice decisions. However, the trustworthiness of some of these methods remains problematic.

The guidance we set out here focuses on a particular approach - **narrative synthesis.** Variants of this approach are widely used in work on evidence synthesis, including Cochrane reviews, but there is currently no consensus on the constituent elements of narrative synthesis and the conditions for establishing trustworthiness – notably a systematic and transparent approach to the synthesis process with safeguards in place to avoid bias resulting from the undue emphasis on one study relative to another – are frequently absent. This guidance therefore aims to contribute to improving the quality of narrative approaches to evidence synthesis.

1.1 Telling stories – the nature of narrative synthesis

Narrative synthesis is sometimes viewed as a 'second best' approach for the synthesis of findings from multiple studies, only to be used when statistical meta-analysis or another specialist form of synthesis (such as meta-ethnography for qualitative studies) is not feasible. In fact, even when specialist methods are used to synthesise findings from multiple studies, those who want to increase the chances of a scientific synthesis being used in policy and practice are likely to find a narrative synthesis helpful in the initial stages of a review. Recognising this, the guidance on undertaking systematic reviews produced by The Centre for Reviews and Dissemination at the University of York suggests that reviewers should first undertake a narrative synthesis of the results of the included studies to help them decide what other methods are appropriate.[1]

Narrative synthesis is a form of story telling. We are part of a story telling culture, and bringing together evidence in a way that tells a convincing story of why something needs to be done, or needs to be stopped, or why we have no idea whether a long established policy or practice makes a positive difference is one of the ways in which the gap between research, policy and practice can start to be bridged. Telling a trustworthy story is at the heart of narrative synthesis.

1.2 Narrative synthesis, narrative reviews and evidence synthesis

'Narrative' synthesis' refers to an approach to the systematic review and synthesis of findings from multiple studies that relies primarily on the use of words and text to summarise and explain the findings of the synthesis. Whilst narrative synthesis can involve the manipulation of statistical data, the defining characteristic is that it adopts a textual approach to the process of synthesis to 'tell the story' of the findings from the included studies. As used here 'narrative synthesis' refers to a process of synthesis that can be used in systematic reviews focusing on a wide range of questions, not only those relating to the effectiveness of a particular intervention.

Narrative review is a phrase some commentators have used to describe more traditional literature reviews and they are typically not systematic or transparent in their approach to synthesis.[2] Narrative synthesis - the focus of this guidance - in contrast, is part of a larger review process that includes a systematic approach to searching for and quality appraising research based evidence as well as the synthesis of this evidence. A narrative review can also be another name for a description, and is used in fields as diverse as performance review of staff[3] to assessing familial patterns in colorectal cancer.[4] Narrative reviews in the sense of traditional literature reviews can be distinguished from narrative synthesis as the latter refers specifically to a specific approach to that part of a systematic review process concerned with combining the findings of multiple studies.

Evidence synthesis includes, but is not restricted to, systematic reviews. Findings from research using a wide range of designs including randomised controlled trials, observational studies, designs

that produce economic and qualitative data may all need to be combined to inform judgements on the effectiveness, cost-effectiveness, appropriateness and feasibility of a wide range of interventions and policies. Evidence syntheses may also addresses many other types of questions including, for example, questions about the current state of knowledge on the causes of particular health or social problems. They are also undertaken in diverse fields from health services research and sociology to engineering and urban planning.

1.3 Why this guidance has been produced?

The Cochrane Collaboration, established in 1993, is an international non-profit and independent organisation, dedicated to making up-to-date, accurate information about the effects of healthcare readily available worldwide. It produces and disseminates systematic reviews of healthcare interventions and promotes the search for evidence in the form of clinical trials and other studies of interventions.

Since its inception, there have been major developments in methods for the systematic review of research evidence which have increased the reliability of the evidence about ***effectiveness*** available to decision makers by combining findings from good quality studies which evaluate policies, specific interventions or professional practices. However, even in reviews focusing on effectiveness, meta-analysis is often an inappropriate approach to synthesis. Additionally, there has been increasing recognition of the need for review and synthesis of evidence to answer questions other than those focusing on effectiveness, particularly those relating to the local implementation of interventions shown to be effective in experimental contexts. Methods for the synthesis of evidence on effectiveness when meta-analysis is not appropriate or for the synthesis of more diverse evidence are, however, not well developed.

Unlike meta-analysis, ***narrative synthesis*** does not rest on an authoritative body of knowledge or on reliable and rigorous techniques developed and tested over time. In the absence of such a body of knowledge there is, as the Cochrane handbook argues[5]

‘a possibility that systematic reviews adopting a narrative approach to synthesis will be prone to bias, and may generate unsound conclusions leading to harmful decisions’

This problem is not confined to narrative synthesis - statistical techniques have produced misleading results in the past (and continue to do so from time to time). However, given the widespread use of narrative synthesis in systematic reviews there is a pressing need for the methodological foundation of this approach to be strengthened, if systematic reviews produced to inform the choice and implementation of interventions are to be credible. This is the aim of this guidance.

1.4 What the guidance is about

The guidance provides advice on the conduct of ***narrative synthesis*** in the context of systematic reviews of research evidence and describes some specific tools and techniques that can be used in the synthesis. The (synthesis) product, *at a minimum,* is a summary of the current state of knowledge in relation to a particular review question. This question might relate to effectiveness or cost effectiveness, to issues of efficacy, appropriateness (to need), feasibility of implementation, or to some or all of these.

We recognise that narrative synthesis can be utilised in reviews addressing a wide range of questions. However, for practical reasons, we have focused this guidance on the conduct of the narrative synthesis of ***research*** evidence in the context of two types of systematic review which have particular salience for those who want their work to inform policy and practice: those addressing questions concerned with the ***effects*** of interventions and those concerned with the ***implementation*** of interventions shown to be effective in experimental settings.

1.5 Who the guidance is for

The guidance is intended to be accessible to a range of people involved in systematic reviewing. However, whilst users of the guidance will not need to be systematic review experts, they will need a

reasonable level of research literacy and we would advise anybody without experience of systematic review work to collaborate with more experienced colleagues.

The phrase *evidence synthesis* can be used to mean many different things. At its most simple, synthesis will involve the juxtaposition of findings from multiple studies, perhaps with some analysis of common themes or findings across studies. More sophisticated approaches to synthesis involve the integration or interpretation of results from multiple studies, with the aim of producing new knowledge/findings. It has been suggested[2] that different types of evidence synthesis can be located along a continuum from quantitative approaches, which involve the pooling of findings from multiple studies (e.g. meta-analysis), to qualitative approaches, which involve an interpretative approach (e.g. meta-ethnography). The guidance provided here lies between these two. Narrative synthesis will always involve the 'simple' juxtaposition of findings from the studies that have been included in the review. However, it may also involve some element of integration and/or interpretation, depending on the type of evidence included. These methods necessarily require some familiarity with research processes if they are to be done well.

1.6 When might the guidance be used?

The process of evidence synthesis is not linear, so reviewers may use a number of different approaches to synthesis in an iterative way. Narrative synthesis might be used:

- Before undertaking a specialist synthesis approach such as statistical meta-analysis or meta-ethnography
- Instead of a specialist synthesis approach because the studies included are insufficiently similar to allow for this
- Where the review question dictates the inclusion of a wide range of research designs, producing qualitative and/or quantitative findings for which other approaches to synthesis are inappropriate.

1.7 Developing the guidance

The methods used in the development of the guidance are described in detail in the appendix and summarised here. The process began with a systematic search of the methodological literature in an attempt to identify existing guidance on the conduct of narrative synthesis and any specific tools and techniques that could potentially be used in the narrative synthesis process. The search process and results are shown in Figure 1.

The search included three elements: i) a database search, ii) a search of internet sites and iii) identification of relevant text by members of the research team. This generated 1,309 items. On the basis of an initial review of titles and, where available, abstracts by at least two members of the research team 264 of these items were retrieved and read in full by at least two members of the research team. This process resulted in 69 articles, reports and/or books being included in the methodological review. None specifically related to narrative synthesis although some elements of guidance on established methodologies such as meta-ethnography and 'case survey' method, for example, were judged relevant to the conduct of narrative synthesis.

Methodological guidance on the conduct of various different approaches to review and synthesis were used to identify common generic elements of an evidence synthesis process. Other text provided 'tips' on aspects of the evidence review process in general, such as how to structure results and/or present data and described a number of specific tools and techniques for the management, manipulation and presentation of quantitative and/or qualitative data. This material formed the basis of an initial draft of the guidance on narrative synthesis. The guidance was then applied to two 'demonstration' syntheses: one focusing on the effectiveness of intervention(s); the other on the implementation of intervention(s). These demonstration syntheses have been incorporated into the final version of the guidance to illustrate how the guidance may be used to inform decisions about which specific tools and techniques to use in the context of a particular review.

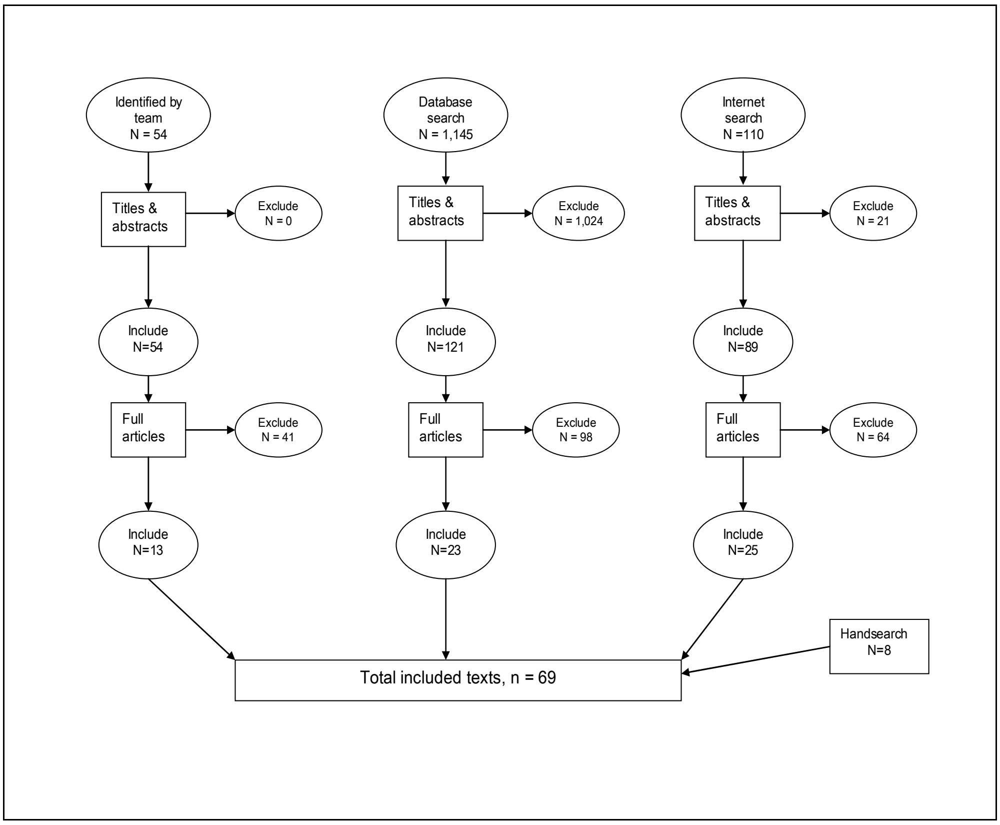

Figure 1. Search process and results

1.8 What the guidance does not do

The guidance does not describe a new approach to the synthesis of qualitative or mixed method research. Instead this guidance seeks to provide an over-arching framework to guide the conduct of a narrative synthesis and suggests ways in which current approaches to narrative synthesis may be further enhanced and developed. Similarly, the guidance is not intended as a source of detailed methodological advice on the systematic review process as a whole. Whilst there is some limited discussion, for example, of search strategies and study quality appraisal, the guidance does not provide details of specific methods for these. We include references to detailed methodological advice in these and other areas in Appendix 2.

CHAPTER 2: THE SYSTEMATIC REVIEW PROCESS – AN OVERVIEW

The process of undertaking a systematic review has been well documented and there is broad agreement about the main elements involved. Six main elements are identified here including the process of synthesis, the focus of this guidance. The other five elements of a systematic review are not described in detail. References to detailed methodological advice on systematic reviewing are included in Appendix 2. This chapter provides a framework to aid understanding of where the synthesis occurs in the systematic review process.

2.1 Identifying the review focus, searching for and mapping the available evidence

Getting the question(s) 'right' is critical to the success of the systematic review process overall. The review question has to be both ***relevant*** to potential users of the review and in theory at least ***answerable***. In some instances the question is clearly formulated at an early stage. More often, however, whilst an initial focus for the review is identified, a '***mapping***' of the available relevant evidence needs to be carried out before the specific question(s) for the review can be clearly specified.[6]

The mapping exercise can be used to assess the need for a systematic review and/or to guide and refine the scope of the review. It is especially useful in situations where a broad question is of interest, such as "how effective are interventions to prevent unintentional injuries?" By mapping the available literature addressing this topic it is possible to:

- Describe the types of interventions that have been evaluated
- Describe the sorts of study designs used in these evaluations and
- Assess the volume of potentially relevant literature.

Based on this initial mapping the scope of the review can be refined, so that the questions to be addressed are both answerable and relevant. The search for studies should be comprehensive and appropriate to the question posed so a mapping exercise may also help to refine a search strategy.

2.2 Specifying the review question

It will take time to get the review question right. In the context of reviews of the effectiveness of interventions, there is general agreement that a well-formulated question involves three key components: the people (or participants) who are the focus of the interventions, the interventions, and the outcomes. Sometimes a fourth component that relates to type of study design is also included. If the review intends to focus on the factors shaping the implementation of an intervention then the question will also have to include components related to this, such as aspects of the context in which the intervention was implemented.

2.3 Identifying studies to include in the review

Once the precise review question has been agreed, the key components of the question form the basis of specific selection criteria, each of which any given study must meet in order to be included in the review. It is usually necessary to elaborate on the key components of the review question so as to aid process of identifying studies to include in the review and make sure that decisions made are transparent to users of the review. These might include, for example, being more precise about the age groups of participants to be included in the review or about aspects of the intervention design.

2.4 Data extraction and study quality appraisal

Once studies are selected for inclusion a process of study quality appraisal and data extraction takes place. Decisions about which data should be extracted from individual studies should also be guided by the review question. In the context of a systematic review addressing a question about the effect of a particular intervention, for example, the data to be extracted should include details of: the

participants, the interventions, the outcomes and, where used, the study design. For reviews focusing on implementation, it would be important to extract detailed data on the design of the intervention, the context in which it was introduced and on the factors and/or processes identified as impacting on implementation. The specific data and/or information to be extracted and recorded are usually those which could affect the interpretation of the study results or which may be helpful in assessing how applicable the results are to different population groups or other settings. This may be referred to as applicability, generalisability or external validity.

Study appraisal - also called validity assessment, assessment of study quality and critical appraisal - usually refers to a process of assessing the methodological quality of individual studies. This is important as it may affect both the results of the individual studies and ultimately the conclusions reached from the body of studies - although ‘quality’ in general and validity in particular are defined differently in relation to different types of study designs. In the context of effectiveness reviews study quality is often used as a criterion on which to base decisions about including or excluding particular studies, although this does depend on the approach taken by the reviewers. Whatever the focus of the review, reviewers may choose to exclude studies from the synthesis on grounds of methodological quality; others may opt to include all studies, but in this case it is important to differentiate clearly between more and less robust studies. There are many different appraisal tools available for use in relation to both quantitative and qualitative study designs and details of how to get information about some of these are provided in Appendix 2.

2.5 The synthesis

The key element of a systematic review is the synthesis: that is the process that brings together the findings from the set of included studies in order to draw conclusions based on the body of evidence. The two main approaches are quantitative (statistical pooling) and narrative, and sometimes both approaches are used to synthesise the same set of data. One approach - narrative synthesis - is the focus of detailed attention in this guidance.

2.6 Reporting the results of the review and dissemination

Once the review is complete the findings need to be disseminated to potential users, although communication needs to be considered from the start often with the involvement of policy, practice and end point users and throughout the review process. We have included some useful references to the ‘art’ of dissemination - an often neglected component of the systematic review process in Appendix 2.

CHAPTER 3: GUIDANCE ON NARRATIVE SYNTHESIS – AN OVERVIEW

As we have noted this guidance focuses on the conduct of narrative synthesis in systematic reviews of research-based evidence on:

- The ***effects*** of interventions and/or
- The factors shaping the ***implementation*** of interventions.

Although we have restricted our focus in this way, the guidance may also be helpful for people focusing on other types of review questions, for example, about the needs and/or preferences of particular population groups or the causes of particular social and/or health problems.

Our aim is to provide broad guidance on ways in which the process of narrative synthesis can be made more systematic and transparent and on how bias introduced by the evidence itself (as a result of methodological shortcomings in the included studies) and/or by decisions made by reviewers (for example, through the process of inclusion and exclusion) can be minimised. The guidance does not provide a set of definitive prescriptive rules on the conduct of narrative synthesis. In our experience the most appropriate approach and the selection of specific tools and techniques for data management and manipulation depends on the nature of the particular review being conducted.

In this chapter we describe a generic framework that identifies four elements of the narrative synthesis process and various tools and techniques that can be used to manage data, manipulate and synthesise findings from multiple studies and present the results of the synthesis. In the following two chapters we describe in detail the practical application of the guidance and particular tools and techniques to the synthesis of two bodies of research-based evidence: one concerned with the effects of an intervention the other concerned with factors influencing the implementation of an intervention.

3.1 A general framework for narrative synthesis

For the purpose of this guidance we have identified four main elements to a narrative synthesis process:

- Developing a theory of how the intervention works, why and for whom
- Developing a preliminary synthesis of findings of included studies
- Exploring relationships in the data
- Assessing the robustness of the synthesis

Figure 2 describes the purpose of each of these four elements of a synthesis in relation to a systematic review focusing on (1) the effects and (2) the factors impacting on the implementation of an intervention/programme.

We are not suggesting that narrative synthesis should proceed in a linear fashion with these elements being undertaken sequentially. In practice, reviewers will move in an iterative manner among the activities we have suggested make up these four elements. We have separated them out and presented them sequentially simply to provide a structure to the guidance. In the following sections we focus on these elements in turn in order to explain the aims of each in more detail. We then provide brief descriptions of tools and/or techniques that may be utilised in the conduct of a narrative synthesis before moving on in the subsequent chapters to demonstrate the practical application of the narrative synthesis framework and the specific tools and techniques.

Main elements of synthesis	Effectiveness Reviews	Implementation Reviews
**1. Developing a theoretical model of how the interventions work, why and for whom**	***Purpose:***<br>• To inform decisions about the review question and what types of studies to review<br>• To contribute to the interpretation of the review's findings<br>• To assess how widely applicable those findings may be	***Purpose:***<br>• To inform decisions about the review question and what types of studies to review<br>• To contribute to the interpretation of the review's findings<br>• To assess how widely applicable those findings may be
**2.Developing a preliminary synthesis**	***Purpose:***<br>• To organise findings from included studies to describe patterns across the studies in terms of:<br>o The direction of effects[1]<br>o The size of effects	***Purpose*:**<br>• To organise findings from included studies in order to:<br>o Identify and list the facilitators and barriers to implementation reported<br>o Explore the relationship between reported facilitators and barriers
**3. Exploring relationships in the data**	***Purpose:***<br>• To consider the factors that might explain any differences in direction and size of effect across the included studies	***Purpose:***<br>• To consider the factors that might explain any differences in the facilitators and/or barriers to successful implementation across included studies<br>• To understand how and why interventions have an effect
**4. Assessing the robustness of the synthesis product**	***Purpose:***<br>• To provide an assessment of the strength of the evidence for:<br>o Drawing conclusions about the likely size and direction of effect<br>o Generalising conclusions on effect size to different population groups and/or contexts	***Purpose:***<br>• To provide an assessment of the strength of the evidence for drawing conclusions about the facilitators and/or barriers to implementation identified in the synthesis. Generalising the product of the synthesis to different population groups and/or contexts

Figure 2. The main elements in a narrative synthesis

Element 1: The role of theory in evidence synthesis

Although not all reviewers may choose to do this, it can be useful to develop a model of what Weiss refers to as an intervention's "theory of change" to inform a systematic review. The "theory of change" describes "*the chain of causal assumption that link programme resources, activities, intermediate outcomes and ultimate goals".*[7] It is concerned with how the intervention works, why, and for whom. Reviewers would normally develop their theory of change at an early stage of a review before the synthesis proper begins. If done early enough an understanding of the theory behind the intervention can inform decisions about the review question and the types of studies to include. In terms of the narrative synthesis, a "theory of change" can contribute to the interpretation of the review's findings and will be valuable in assessing how widely applicable those findings may be. Information on programme theory may come from explicit statements in study reports on the goals of the intervention (who it is intended to affect, in what way and how) and from other reviews. The theory can be presented in narrative form or as a diagram like the one reproduced below in Figure 3.

Theory building and theory testing is a neglected aspect of systematic reviews. Shadish (1996) has pointed out that meta-analysis for example has focused too much on descriptive causation (simply describing the size of an effect) and too little on the development of explanatory theories.[8] Yet systematic reviews - whether of qualitative or quantitative research - are likely to be much more powerful than single studies for these purposes. In turn systematic reviews can contribute to developing and testing the limits of theories, by examining how contextual or temporal variables moderate outcomes. Theories themselves can also be the subject of systematic reviews.[9-13]

[1] The notion of 'effects' should not be taken for granted. In some reviews the synthesis process will involve the reviewers in a process intended to help to understand what the effects of a particular interventions or programme are. This is particularly the case when the effects are presented in narrative form rather than in numerical form or derived from structured questionnaires/indicators.

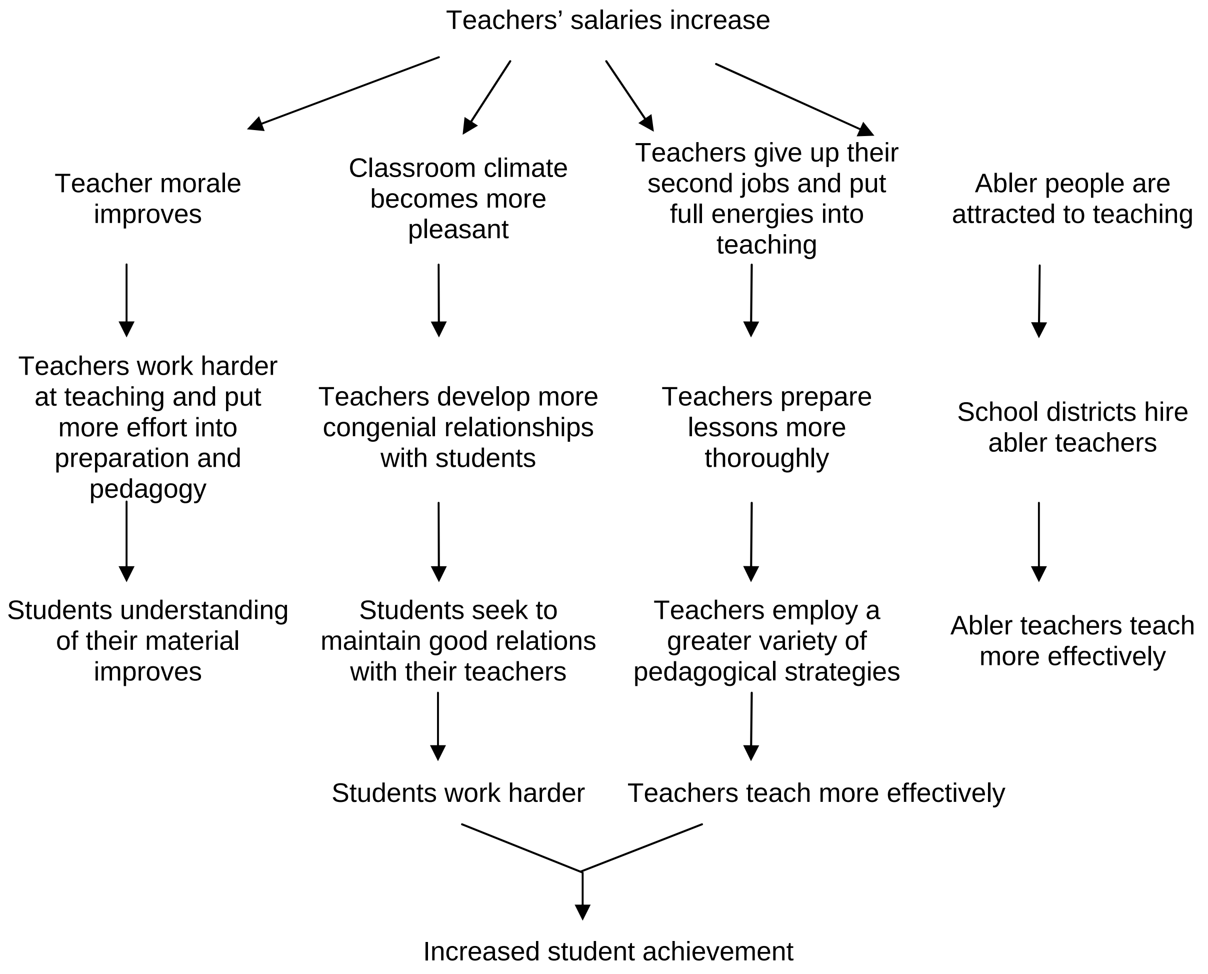

Figure 3. Example of a Programme Theory model: mechanisms by which higher teachers' pay may be linked to increased student achievement (from Weiss, 1998)

Element 2: Developing a preliminary synthesis

Whatever the focus of the review, the purpose of the preliminary synthesis is to develop an initial description of the results of included studies. It is important to remember that the product of this initial process will only be ***preliminary,*** rather than an end in itself. It will always be necessary to interrogate the preliminary synthesis to identify factors that have influenced the results reported in included studies i.e. to begin to construct an explanation of how and why a particular intervention had the effects reported; of how and why particular factors/processes impinged on implementation, and to test the robustness of the results of the synthesis. This is the purpose of other elements of the synthesis process described below.

During the preliminary synthesis, reviewers focusing on the effects of an intervention will need to organise the results of the included studies so they are able to describe patterns across them in terms of both the direction and size of the effects reported. In relation to a review on implementation, the studies need to be organised so that patterns in the factors/processes that are reported as impacting in some way on the implementation of an intervention can be identified across the studies. Assuming that study quality appraisal has been carried out at the same time as data extraction these details will be available during the whole of the synthesis process although quality was not examined in our demonstration synthesis reported later until near the end of the synthesis.

Element 3: Exploring relationships within and between studies

As patterns across study results begin to emerge from preliminary attempts at a synthesis reviewers should begin to subject these to rigorous interrogation in order to:

- Identify any factors that might explain differences in direction and size of effect across the included studies or in the type of facilitators and/or barriers to successful implementation
- To understand how and why interventions have or do not have an effect or why particular barriers and/or enablers to implementation operate

At this point in the synthesis the reviewers move beyond identifying, listing, tabulating and/or counting results to exploring relationships within and across the included studies. The relationships of interest are of two broad types:

- Those between characteristics of individual studies and their reported findings
- Those between the findings of different studies

Some of the studies included in a review may have reported information about relationships between study characteristics and reported findings, in which case the job of reviewers is to compare and contrast the ways in which the relationships have been identified and analysed across the studies. In other cases little attention may have been paid to these relationships. The practical work involves using data previously extracted from primary studies to look at the relationships between study results and key aspects of the primary studies, and comparing and contrasting these relationships across the studies. This element of a narrative synthesis can be very time consuming but it is critical to the quality of the process as a whole.

Exploring the influence of heterogeneity is important at this stage of the synthesis process. We have already noted that a primary reason for choosing a narrative approach to synthesis in a systematic review about the effects of an intervention is because there is considerable heterogeneity in the included studies in terms of methods, participants, interventions and via other unknown sources. There are also likely to be differences between studies in terms of their findings – whether quantitative or qualitative. This too may be due to known differences between the studies, including methodological differences, and differences in the baseline characteristics of populations being studied.[13] Narrative methods have long been recognised as useful for investigating heterogeneity across primary studies and developing an understanding of which aspects of an intervention may be responsible for its success[14] or investigating the possibility that study variation is attributable to theoretical variables.[15]

Many social or behavioural interventions are complex because of the characteristics of the interventions, study population/s, outcomes, or other methodological issues relating to the conduct of the primary studies.[16] Further complexity is introduced because any effects of the interventions may be modified by context, and the intervention itself may vary when it is being implemented.[17-19] Because of these variations, reviewers of complex interventions may expect considerable heterogeneity across studies and need to consider this when synthesising results.

"Social" heterogeneity may incorporate not only socio-demographic and individual differences, but also historical, cultural, spatial and other differences that may affect both the delivery and impact of the interventions being reviewed. Some of the main sources of variability that reviewers need to consider when 'testing' the robustness of the patterns emerging from the included studies are outlined below (adapted from guidance produced by the Cochrane Health Promotion and Public Health Field).[20]

Variability in outcomes

In systematic reviews of clinical interventions variation in outcomes is termed clinical heterogeneity. Variation also exists in social research, however, given the longer causal chains for many social interventions (including public health interventions), proximal/immediate, intermediate, and distal/long term outcomes may be reported. Whilst the synthesis would ideally seek to address all these outcomes in practice it is often not feasible to do this.

Variability in study designs

Methodological diversity is common in systematic reviews of social interventions. Where the main potential sources of variation are known, heterogeneity between effects can be explored by means of subgroup analysis, based for example on theories about how the intervention works, and for which

groups. For many social and public health interventions, theories about mechanisms and interactions may be under-developed and the exploration and interpretation of heterogeneity complex. It may therefore be difficult to anticipate the main sources of heterogeneity *a priori*.

Variability in study populations, interventions and settings
The content of complex social interventions may vary between specific settings or populations. Some of the variability may be intentional as interventions are tailored to local needs (including characteristics which may influence the outcomes of interest such as race, gender, and socio-economic position).

As noted earlier an understanding of the interventions 'theory of change' will be particularly valuable when exploring the influence of heterogeneity especially when interpreting differences between subgroups of studies (post-hoc sub group analyses). The findings of individual studies will vary with study characteristics such as intervention type, quality and extent of implementation, and the study setting, and may vary between different subgroups of participants. Developing plausible explanations for these differences (some of which will be due to chance) is difficult but sub-group findings that are supported by an *a priori* rationale (that is, which have been described in the programme theory) are more plausible than those which are not.

The extent to which reviewers are able to consider the impact of context in systematic reviews evaluating the effects of interventions or factors impacting on implementation will depend on the availability of relevant information in the included studies. Typically, reviews focusing on effects do not consider the context in which an intervention is implemented in great depth. Given that implementation studies are focusing specifically on how dimensions of context (alongside other factors) impinge on implementation, the data available in these studies should be much richer. However, research has suggested that there may be a particular problem with inadequate reporting of research methods in these studies.[21] The dimensions of context which might be relevant to exploring differences in the reported results of included studies will depend on the nature of the intervention with which the review is concerned.

Other factors to be considered in this exploration of factors mediating the impact of an intervention, or explanations of how or why it has a particular impact, may not be able to be extracted from studies as 'data'. These include information about the general approach taken by the researchers both in terms of theory and methods.

Element 4: Assessing the robustness of the synthesis

The notion of robustness in relation to evidence synthesis is complex. Most straightforwardly robustness can be used to refer to the methodological quality of the primary studies included in the review and/or the trustworthiness of the product of the synthesis process. Obviously, these are related. The trustworthiness of a synthesis will depend on both the quality and the quantity of the evidence base it is built on. If primary studies of poor methodological quality are included in the review in an uncritical manner then this will affect the trustworthiness of the synthesis.

The trustworthiness of the synthesis will also depend on the methods used in the synthesis. This will depend on the measures taken to minimize bias, ensuring, for example, that studies judged to be of equal technical quality are given equal weight or if not providing a sound justification for not doing so. Another less straightforward aspect of robustness that can impact on the trustworthiness of the synthesis is the extent to which reviewers have enough information to judge that individual studies meet the criteria for inclusion. This can be a significant problem with reviews of complex interventions. Authors of primary studies often fail to provide adequate information on the intervention they are focusing on and there can be inconsistency between studies in the definition of what constitutes a particular intervention. It is particularly important that reviewers give detailed information about the interventions they plan to include and exclude from a review: for example, stating that 'psychological interventions are eligible' is unlikely to be adequate.

Towards the end of the synthesis process, therefore, the analysis of relationships within and between studies described above should lead into an overall assessment of the strength of the evidence available for drawing conclusions on the basis of a narrative synthesis. This should include systematic attention to all three elements of robustness discussed above.

It is particularly important that the results of any appraisal of the methodological quality of included studies be considered in a systematic manner. Whilst there are well-established methods for assessing the quality of intervention studies, this is not the case in relation to studies of implementation processes, qualitative research or mixed methods research in general so there are no approaches to quality assessment that can be recommended in these situations. Additionally, the results of the appraisal process may or may not have been used to exclude some studies on methodological grounds. Whatever approach to quality appraisal is adopted, (probably at an earlier stage of the review process) this information should inform the assessment of the strength or weight of the evidence available to support conclusions drawn on the basis of the synthesis process.[6]

3.2 Tools and techniques for narrative synthesis

In this section we provide brief descriptions of the tools and techniques we have identified which can be used in the process of narrative synthesis. We have divided these into those which appear to be most appropriate for use in each of the three analytical elements of the synthesis.

At the beginning of each sub-section below the main tools and techniques are listed in a table. As we have noted, decisions about which of these are appropriately used in a specific synthesis will be determined by the nature of the evidence being synthesised as will be illustrated in the practical applications of the guidance.

Before describing the tools and techniques a general comment about the visual representation of data from included studies is warranted. Many of the specific tools and techniques described involve visual representation and this can be invaluable at all stages of a synthesis. However, it is important to recognise that visual representation of data is not sufficient in itself as a synthesis. As Evans[22] has argued, for example, tabulation and other visual representations of data tend to reduce studies to their key characteristics neglecting aspects that could be important in understanding the patterns revealed. He draws a distinction between ‘descriptive synthesis’ and ‘interpretive synthesis’ and is critical of the heavy reliance placed by some reviewers on synthesis by tabulation. For commentators such as Evans, the relationship between the visual representation of data (the descriptive synthesis) and the narrative elaboration of the patterns identified (the interpretative synthesis) is critical to the quality of a narrative synthesis.

Element 1: Tools and techniques for developing a theory of change

We have not identified specific tools or techniques for use in the development of a theory of change although some of those described for use at other points in the synthesis process may also inform theory development and elaboration - as highlighted in the practical application of the guidance in chapters four and five.

Element 2: Tools and techniques for developing a preliminary synthesis

1.	Textual descriptions of studies
2.	Groupings and clusters
3.	Tabulation
4.	Transforming data into a common rubric
5.	Vote counting as a descriptive tool
6.	Translating data; thematic analysis
7.	Translating data: content analysis

Textual descriptions

A simple starting point in a preliminary synthesis is to produce a descriptive paragraph on each included study - it may also be useful for recording purposes to do this for all excluded studies as well. In many reviews this will have been completed at an early stage in the review process and it can be done for any type of study. It is important that these narrative descriptions are produced in a systematic way, including the same information for all studies if possible and in the same order. Some reviewers have suggested that studies considered more important in terms of what they offer the review may be discussed at greater length, while briefer discussion may be afforded to less central or informative studies.[23] In theory this is a way of giving more weight to higher quality or larger studies within a narrative synthesis. However, it is difficult to determine how much “weight” in terms of description/discussion should be allotted to individual studies and how this should vary with methodological quality, for example. Additionally, if textual descriptions are produced at an early

stage of the review process it will not be possible to give more weight to one study over another and hence a fuller description because methodological quality and other aspects of relevance will not yet have been assessed. Whilst textual descriptions are a useful way for reviewers to become familiar with the included studies and to begin to compare and contrast findings across studies, it can be very difficult to discern patterns across studies from these textual descriptions, particularly when there are a large number of studies.

Groupings and clusters
There can be considerable variation in the number of studies included in systematic reviews. Some Cochrane reviews, for example, conduct the synthesis on a very small number of studies, often because of very tightly defined inclusion/exclusion criteria and/or to a paucity of research addressing the question of interest. Other reviews include large numbers of studies in the pool to be synthesised. In most cases the number of studies included will be determined by the size and quality of the existing literature. Whilst including findings from large numbers of studies can be labour intensive, the analytical process involved in statistical meta-analyses can readily manage large numbers. This is not the case with narrative synthesis. Usually therefore, a process of narrative synthesis will involve organising the included studies into smaller groups to make the process more manageable. Although the reviewers may start to group the included studies at an early stage of the review, it may be necessary to refine these initial groups as the synthesis develops.
Organising the included studies into groups can also be a useful way of aiding the process of description and analysis and looking for patterns within and across these groups. It is important to use the review question(s) to inform decisions about how to group the included studies. Studies can be grouped according to one or a combination of the following: the type of intervention being studied; the setting or context for the intervention (school or community based interventions for example); the group at whom it is being directed (different age groups, for example); the study design; and/or the nature of the results being reported (different outcome measures for example, or different types of factors impacting on implementation).

Tabulation
Tabulation is a common approach used in all types of systematic review to represent both quantitative and/or qualitative data visually - indeed many of the examples of approaches to description included in this guidance are presented in tabular form. Tabulation can be useful at any stage of the preliminary synthesis process according to the preference of reviewers. It can be particularly useful in helping to develop an initial description of the included studies and to begin to identify patterns across studies. They are typically used to provide details of study design, results of study quality assessment, outcome measures and other results. These data may be presented in different columns in the same table or in different tables. Used thoughtfully, tabulation can be a valuable tool in the preliminary synthesis of results across studies and can provide important building blocks for future elements of the synthesis process.[22, 24-26]

Some authors stress the need to take care with the layout of tables, arguing that the way in which data are tabulated may affect readers' impression of the relationships between studies. For example, 'placing a results column adjacent to any of the characteristics or quality columns could invite speculation about correlation and association'.[24] These notes of caution point to the importance of the reviewers' attempting some narrative interpretation of tabulated data.[22, 24]

Transforming data: Constructing a common rubric across quantitative studies
The results of studies included in a review may take different numerical and/or statistical forms.[2] In these situations reviewers need to transform results into a common numerical/statistical rubric if possible. When extracting data from quantitative studies, it is standard practice to extract the raw or summary data from included studies wherever possible, so a common statistic can be calculated for each study, e.g. converting dichotomous data into odds ratios or relative risks and continuous data (if from different measurement scales) into standardised mean differences (SMD). In a review of effectiveness which incorporates a statistical meta-analysis these results would be pooled to provide a single estimate of effect. In a narrative synthesis study results will not be pooled statistically, so the process cannot provide a new single estimate of effect. However, transforming study results into a

[2] The distinction being made here, between numerical and statistical, relates to the possibility that figures provided as percentages, for example, would not accurately be described as statistics.

common rubric will allow reviewers to develop a meaningful summary of study results and a more robust assessment of the range of effects that would be anticipated from a particular intervention.

Vote-counting as a descriptive tool
Although some commentators[27] have argued strongly against 'vote counting' calculating the frequency of different types of results across included studies can be a useful way of producing an initial description of patterns across the included studies.[28] Indeed, it could be argued to be an intrinsic element of the preliminary stages of any narrative synthesis. In the case of reviews evaluating the effects of an intervention, a simple approach to vote-counting might involve the tabulation of statistically significant and non-significant findings. Some reviewers have developed more complex approaches to vote counting, both in terms of the categories used and by assigning different weights or scores to different categories.

The interpretation of the results of any vote counting exercise is a complex task. According to some methodologists writing about vote counting, the category with the most studies "wins".[29] Similarly in the context of reviews of effects, some commentators argue that the statistical significance category 'containing the largest number of studies represents the direction of the true relationship'.[30] However, it has also been argued that, this approach to synthesis "tends to give equal weight to studies with different sample sizes and effect sizes at varying significance levels, resulting in misleading conclusions".[31] There are examples where vote counting has been compared with other methods of synthesis and major differences in findings have been reported.[32-34] So, whilst vote counting can be a useful step in a preliminary synthesis the interpretation of the results must be approached with caution and these should be subjected to further exploration of relationships between data/findings within and across the included studies.

Translating data: thematic and content analysis
Where results are presented in the form of themes or concepts, as is the case in qualitative research or some surveys, studies focusing on similar topics may have conceptual overlaps, even if these are not apparent from the way the results are reported. Alternatively, apparently similar concepts in different studies may actually be referring to different phenomena. In this context a process of 'translation' of primary themes or concepts reported across studies can be used to explore similarities and/or differences between different studies.[35] Where studies involve both qualitative and quantitative data reviewers may decide to construct a common rubric for the synthesis – this could involve transforming qualitative findings into quantitative form or vice versa. Both thematic analysis and content analysis can help in this process of 'translation' or 'interpretation' as it is sometimes referred to.

Thematic analysis
Thematic analysis, a common technique used in the analysis of qualitative data in primary research, can be used to identify systematically the main, recurrent and/or most important (based on the review question) themes and/or concepts across multiple studies. Although usually used with qualitative data some people have argued that it could be used with studies involving quantitative data or data from mixed method studies. For example the variable labels included in survey research may be extracted as 'themes' in the same way as conceptual themes are extracted from qualitative research reports.[36] Thematic analysis provides a means of organising and summarising the findings from large, diverse bodies of research. The analysis would typically, but not invariably, be developed in an inductive manner; i.e. without a complete set of a priori themes to guide data extraction and analysis from the outset. Thematic analysis tends to work with, and reflect directly, the main ideas and conclusions across studies, rather than developing new knowledge although this is possible.

There are problems with thematic analysis from the perspective of a systematic review. The process can, for example, be associated with a lack of transparency – it can be difficult to understand how and at what stage themes were identified. The results of the synthesis might look very different if an entirely a priori, theoretically-driven approach had been used as against an inductive approach. In this context it is important that reviewers give as much detail as possible about how a thematic analysis was conducted.

Content analysis
Content analysis was developed as an analytical approach for primary research, but it is readily applied to the synthesis of findings from multiple studies. Content analysis has been defined as 'a

systematic, replicable technique for compressing many words of text into fewer content categories based on explicit rules of coding.'[37] Unlike thematic analysis, it is essentially a quantitative method, since all the data are eventually converted into frequencies, though qualitative skills and knowledge of underlying theory may be needed to identify and characterise the categories into which findings are to be fitted.

Element 3: Tools and techniques for exploring relationships

1.	Graphs, frequency distributions, funnel plots, forest plots and L'Abbe plots
2.	Moderator variables and sub-group analyses
3.	Idea webbing and conceptual mapping
4.	Translation : reciprocal and refutational
5.	Qualitative case descriptions
6.	Investigator/methodological triangulation
7.	Conceptual triangulation

Graphs, frequency distributions, funnel plots, forest plots and L'Abbe plots.
There are several visual or graphical tools that can help reviewers explore relationships within and between studies, although these are typically only useful in the context of quantitative data. These include:

- presenting results in graphical form
- plotting findings (e.g. effect size or factors impacting on implementation) against study quality
- plotting confidence intervals; and/or plotting outcome measures

Frequency distributions, funnel plots, forest plots, and L'Abbé plots are other possibilities. These tools do not provide any overall interpretative synthesis of the data presented in the plot. There may be good reasons for reviewers not to provide an overall interpretative synthesis of the data presented graphically but it is normally good practice to do so and if not done then it is important that reviewers 'explain' their reasons for not presenting an overall narrative synthesis of these types of representations of data.

Moderator variables and subgroup analyses
There is a growing consensus that when evaluating the impacts of interventions the important questions are "what works, for whom, and in what circumstances". One approach to answering these questions when findings are quantitative is by means of analysing moderator variables – variables which can be expected to moderate the main effects being examined by the review. This can be done at the study level, by examining characteristics that vary between studies (such as study quality, study design or study setting) or by analysing characteristics of the sample (such as groups of outcomes, or participants), based on some underlying theory as to the effects of those variables on outcomes. An analysis of moderator variables can be guided by questions such as:

- What are the moderators that the authors of the primary studies identify?
- What are the contributing factors that appear to recur across the studies even if they have not been explicitly identified by authors as moderators?
- How much difference do the likely moderators appear to make to the study results?
- What possible relationships are there among the moderators?

One approach currently used to explore moderators is to examine the effects of interventions across different social groups. Systematic reviewers have argued for some years for the importance of exploring moderator effects in systematic reviews.[8, 38, 39] Methodological groups working within the Cochrane Collaboration have also contributed extensive empirical and other work on these issues. For example the Cochrane Methods Group in Subgroup Analysis has demonstrated some of the methodological and epistemological pitfalls. A new Joint Cochrane Campbell Methods Group has also been formed focusing on equity issues in systematic reviews and exploring the effects of socio-economic moderators will be an important focus for this group. Explorations of effects in subgroups can also play an important role in testing and developing theory in systematic reviews. They can be an important tool for assessing the strength of relationships, for testing the limits of theoretical concepts and explanations, and can contribute to the development of new theories.[10 13]

Developing conceptual models

There are a number of approaches to exploring relationships within and across the studies included in a systematic review that can be broadly described as conceptual models. The basic idea underpinning these approaches is (i) to group findings that reviewers decide are empirically and/or conceptually similar and (ii) to identify (again on the basis of empirical evidence and/or conceptual/theoretical arguments) relationships between these groupings. The approaches often involve visual methods to help to construct groupings and relationships and to represent the final product of this process. Three specific approaches were identified in the methodological literature review conducted to support the production of this guidance: idea webbing, conceptual mapping and conceptual triangulation. Although we describe them separately below they are very similar as we discuss in the demonstration syntheses reported in chapter 4 and 5. It is perhaps worth noting that these tools can also be used to develop review questions and to begin to identify moderator variables to be explored in more detail before the synthesis begins but we do not discuss these uses in this guidance.

Ideas webbing

Ideas webbing suggested by Clinkenbeard,[29] as a method for conceptualising and exploring connections among the findings reported by the studies included in a review. This approach uses spider diagrams to develop a visual picture of possible relationships across study results.

Concept mapping

Mulrow, Langhorne & Grimshaw[40] describe a similar process which we refer to as concept mapping. Their approach involves linking multiple pieces of evidence extracted from across individual studies included in a review to construct a model highlighting key concepts or issues relevant to the review question and representing the relationships between these. This approach uses diagrams and flow charts to visually represent the relationships being explored. The notion of c*onceptual* triangulation described by Foster appears to be very similar in that it is concerned to explore relationships between data drawn from within and between studies.[41] Foster argues that this approach alleviates ‘concerns about combining numbers and text because both qualitative and quantitative results can be portrayed conceptually”. The approach relies heavily on tables to facilitate the analysis and produces a number of possible *models* through which the phenomenon of interest may be better understood on the basis of the diverse sources of evidence synthesised.

Translation as an approach to exploring relationships

Translation as a process for synthesis is typically associated with the work of Noblit & Hare on meta-ethnography.[35] It is a way of using qualitative research techniques to synthesise findings from multiple studies. The term ‘meta’ in this context refers to the *translation* of studies into one another. Although developed for use with qualitative research, the approach could be used with a mixture of qualitative and quantitative evidence. Translation focuses on seeking a common rubric for salient categories of meaning, rather than the literal translation of words or phrases. Noblit and Hare identify two different types of ‘translation’:

1. *Reciprocal translation* (accounts are directly comparable)
2. *Refutational translation* (the accounts are oppositional)

In practice there are few examples of refutational translation in the literature. Having translated the studies into one another, they suggest that reviewers should develop a ‘line of argument’ drawing inferences from the results of the translation. The line of argument is developed by examining similarities and differences between cases to integrate them in a new interpretation that ‘fits’ all the studies. Meta-ethnography is a specialist approach to synthesis (akin to statistical meta-analysis with quantitative studies) and not therefore an approach to be utilised in full in the context of a narrative synthesis. However, the translational process may be of value as a way of exploring relationships across studies. The inductive nature of the process means it is emergent, the initial question or area of interest may be adapted or redirected, and there are numerous judgement calls along the way. Of course the same can be argued for other types of synthesis.

Qualitative case descriptions

As Light and Pillemer note, formal statistical procedures may be able to detect subtle differences in effectiveness but they do not necessarily explain them.[14] These authors argue that ‘qualitative case descriptions’ are particularly valuable in helping with the interpretation of statistical findings. However,

they give relatively little practical advice about how one would go about doing this type of case description. In general terms qualitative case description would seem to include any process in which descriptive data from studies included in a systematic review are used to try to explain differences in statistical findings, such as why one intervention outperforms another (ostensibly similar) intervention or why some studies are statistical outliers. As an example of this process they suggest that in a review of the effectiveness of educational programmes the reviewers might use a range of information from the included studies to seek to answer questions such as:

- What are the characteristics of successful implementations?
- How were the teachers trained?
- How were parents involved?
- What were the details of the educational programme?

This kind of descriptive information may or may not be reported in the original study reports. The textual descriptions of studies described earlier would be a potential resource for this type of work.

Investigator triangulation and methodological triangulation
Approaches to triangulation focus on the methodological and theoretical approaches adopted by the researchers undertaking the primary studies included in a systematic review. Consideration of how these differ across the included studies may be helpful in exploring the nature and impact of moderators in quantitative research or broader relationships in qualitative research. Some authors argue that by working with a number of different triangulation approaches reviewers can develop a better understanding of how the various factors involved in the intervention and its evaluation may have impacted on the results reported in included studies.[42]

Investigator triangulation was developed by Begley to explore the extent to which heterogeneity in study results may be attributable to the diverse approaches taken by different researchers.[42] The approach involves analysing the data in relation to the context in which they were produced, notably the disciplinary perspectives and expertise of the researchers producing the data.[42] Begley is focusing on primary research but this approach could be valuable for evidence synthesis too. It works from the understanding that each disciplinary approach may have produced different kinds of findings. Considering what kinds of evidence and what kinds of outcomes emerge from studies conducted by researchers from particular disciplinary and epistemological positions is potentially an illuminating way to think about possible sources of heterogeneity. This approach will be easier if the review is being undertaken by a multidisciplinary research team “allowing data to be subjected to a range of disciplinary gazes”.[43]

Methodological triangulation was developed by Maggs-Rapport and offers a broadly similar approach.[44] Both of these approaches serve as a reminder that the evidence being synthesised in a systematic review does not offer a series of discrete ‘answers’ to a specific question. Rather, each ‘piece’ of evidence offers a partial picture of the phenomenon of interest. The product of the systematic review, particularly in the case of narrative synthesis, may not be a ‘meta-answer’ to the review question, but a theoretical insight and/or a new model that informs understanding about the mechanisms underlying the results reported.

Element 4: Tools and techniques for assessing robustness of the synthesis

1.	Weight of Evidence – e.g. the EPPI approach
2.	Best Evidence Synthesis
3.	Use of validity assessment – e.g. the CDC approach
4.	Reflecting critically on the synthesis process
5.	Checking the synthesis with authors of primary studies

Weight of Evidence – the EPPI approach
The Weight of Evidence approach developed by staff of the EPPI-Centre is used in many EPPI-Centre reviews.[45] In the EPPI approach relevance criteria are set for a particular review and studies are then assessed for relevance using these. Those that are judged to be relevant are then assessed for methodological quality.

Best Evidence Synthesis (BES)
BES deals with the robustness in terms of the methodological quality of included studies though the application of inclusion criteria. This is based on an approach described by the educational researcher Robert Slavin.[46, 47] In BES, only studies that meet minimal standards of methodological adequacy and relevance to the review are included, and information is extracted in a common standard format from each study, with a systematic approach to the assessment of study quality and study relevance. This approach is not prescriptive about the study designs which can to be included in a review – this can vary, depending on the review question. BES aims to identify and synthesise sources of evidence no matter how diverse. It has been suggested however that BES is simply an example of good systematic review practice albeit with some problems. Suri, for example, suggests that in extracting data from the primary studies BES tends towards calculating the median effect size, rather than calculating a weighted mean effect size, as is standard meta-analytic practice.[31]

Although BES accounts cover the whole review process the approach focuses in particular on the selection of studies into a systematic review rather than focusing on the synthesis, thus emphasising that decisions about study quality should be taken early in the review process to ensure that the review is based on robust evidence. The decision about "strength of evidence" is therefore made early in the review process, and its practical application can be seen in the inclusion and exclusion criteria. For this reason the demonstrations of the application of the narrative synthesis guidance reported in the next two chapters were not able to utilise the approach to check the robustness of the synthesis findings.

Use of validity assessment – Centre for Disease Control (CDC) approach
Other approaches to assessing the strength of evidence included in evidence synthesis have been developed. For example, specific rules may be used to define explicitly what is meant by "weak", "moderate" or "good" evidence. There are numerous examples of this form of synthesis but few from the social sciences. One recent example from healthcare comes from the CDC Community Guide to Preventive Services. [48] In this approach, the reasons for determining that the evidence is insufficient are: A. Insufficient designs or executions, B. Too few studies, C. Inconsistent, D. Effect size too small, E. Expert opinion not used. The categories are not mutually exclusive. While the criteria can be debated, the grounds on which the decision about strength of evidence is made are at least explicit. Many other healthcare evidence grading systems use a similar approach.

Reflecting critically on the synthesis process
Busse et al[49] recommend that in reporting the results of a systematic review a summary discussion section should be provided including the following:

- Methodology of the synthesis used (especially focusing on its limitations and their influence on the results)
- Evidence used (quality, validity, generalisability) – with emphasis on the possible sources of bias from the sources of evidence used and their potential influence on results of the synthesis
- Assumptions made
- Discrepancies and uncertainties identified (the way that any discrepancies in findings between included evidence were dealt with in the synthesis should be discussed and wherever the evidence is weak or non-existent, areas where future research is needed can be highlighted)
- Expected changes in technology or evidence (e.g. identified ongoing studies)
- Aspects that may have an influence on the implementation of the technology and its effectiveness in real settings
- Such a summary would enable the analysis of robustness to temper the synthesis of evidence as well as indicating how generalisable the synthesis might be.

Checking the synthesis with authors of primary studies
In the context of their meta-ethnography of qualitative research Britten et al suggest consulting the authors of included primary studies in order to test the validity of the interpretations developed during the synthesis and the extent to which they are supported by the primary data.[50] This is most likely to be useful where the number of primary studies is small but the authors of the primary studies may have useful insights into the possible accuracy and generalisability of the synthesis.

3.3 Conclusion

In this chapter we have provided an overview of the four main elements of the narrative synthesis process that we have identified and briefly described various tools and techniques that can be used at different points in the synthesis process. In the next two chapters we describe in detail the practical application of the guidance, including the use of particular tools and techniques, to the synthesis of two bodies of research evidence. Chapter four focuses on a narrative synthesis of the findings of the 11 RCTs included in the Cochrane systematic review of interventions for promoting smoke alarm ownership and function.[51] The original Cochrane review involved a meta-analysis which means we are able to compare the results/conclusions of the two approaches to synthesis. Chapter five focuses on the narrative synthesis of studies of the implementation of domestic smoke alarm promotion interventions. This is linked to an earlier pilot review and some comparisons with the outcomes of this earlier work are made.[21, 52]

CHAPTER 4: APPLYING THE GUIDANCE 1: A NARRATIVE SYNTHESIS OF STUDIES OF THE EFFECTIVENESS OF INTERVENTIONS FOR PROMOTING SMOKE ALARM OWNERSHIP AND FUNCTION

4.1 Introduction

The aims of this chapter are to:

- Illustrate in practical terms the decision making processes involved in the application of the guidance to a specific narrative synthesis
- Identify factors that should inform choices about the use of particular tools and techniques in the context of a specific synthesis
- Provide examples of how particular tools and techniques can be used in the synthesis of evidence on effectiveness
- Demonstrate the type of outcomes achieved by a narrative synthesis
- Compare the outcomes of a narrative synthesis of the effect of an intervention with those produced by meta-analysis.

The review selected for comparison was a Cochrane review investigating the effects of interventions for promoting smoke alarm ownership and function.[51] This review was selected because it was methodologically sound, had incorporated a meta-analysis, had analysed a 'manageable' number of studies (11 RCTs) and because it complemented the systematic review of the implementation of smoke alarm promotion interventions that was also being resynthesised in a concurrent 'testing' of the guidance.

- Developing a theory of how the intervention works, why and for whom
- Developing a preliminary synthesis
- Exploring relationships within and between studies
- Assessing the robustness of the synthesis

Within each of these sections, the guidance presents a number of related tools and techniques that can be used to complete the various stages of the synthesis. To apply this guidance to the narrative synthesis, each of the sections was read through in sequential order, and for each element the tools and/or techniques that appeared to be useful and relevant to the synthesis at hand were selected. The reasons for selecting or rejecting a tool or technique are given within each section. Where possible, the tools and techniques employed were used to derive conclusions about the effects of interventions for promoting smoke alarm ownership and function. Where tools or techniques proved to be less useful, this is discussed. A flow chart summarising the synthesis process is presented in figure 4.

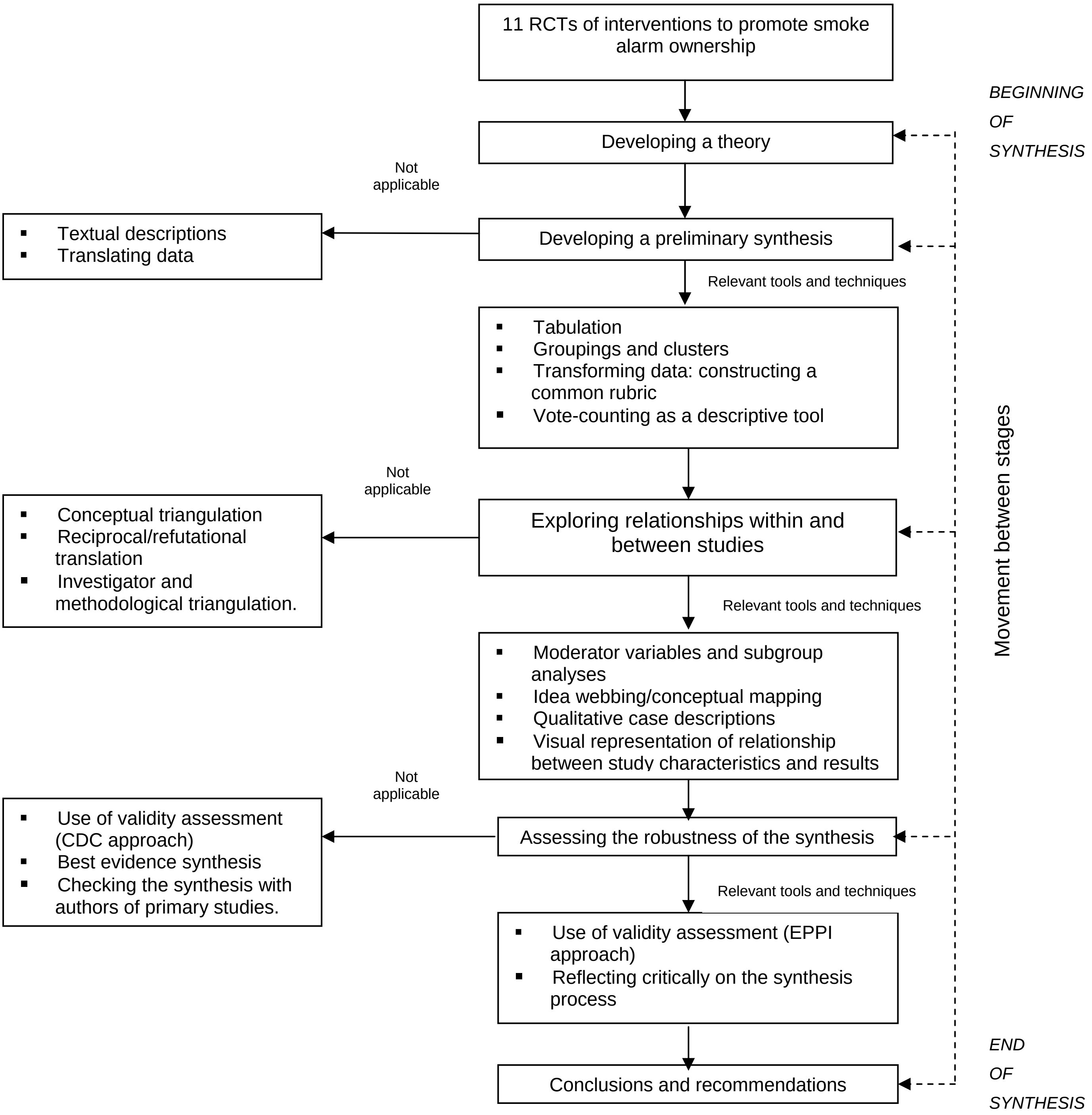

Figure 4: Synthesis process

4.2 Developing a theory

The majority of studies aimed to increase smoke alarm ownership and function through the use of educational interventions with or without the addition of free or discounted smoke alarms for participants. The primary studies did not clearly describe the theoretical basis of the evaluated interventions, but the implicit theory underlying most educational interventions was that education can increase knowledge of potential fire/burns risks, change risk perceptions and lead to behaviour change (i.e. acquisition of smoke alarms). The use of discounted or free smoke alarms as an intervention to increase ownership and function (usually in lower income families) suggests that authors consider cost to be a barrier to smoke alarm acquisition.

4.3 Developing a preliminary synthesis

It is stated in the guidance that "how a reviewer approaches the preliminary synthesis... will depend in part on whether the evidence to be synthesised is quantitative, qualitative or both". In the case of this example, the data to be synthesised were anticipated to be predominantly quantitative and, more specifically, derived entirely from randomised controlled trials. With this in mind each of the tools and techniques presented in the 'preliminary synthesis' section of chapter three were evaluated as to whether they would be relevant for the synthesis at hand (see table 1 below).

Table 1: Selection of tools and techniques in developing a preliminary synthesis

Name of tool/technique	Thoughts/ideas/comments in relation to current synthesis	Should this tool/technique be applied here?
Textual descriptions	Need to determine which aspects of each study will be drawn from the reports. These might be the same as the table headings	Possibly, but not necessarily as a first step
Groupings and clusters	If possible, organise studies by intervention type, context, target population, study design, outcomes. Maybe have 'primary clusters' e.g. (intervention type, population) and have 'secondary clusters' (e.g. study design, context) within these	Yes
Transforming data: constructing a common rubric	Odds ratios or relative risks for dichotomous data, weighted or standardised mean difference for continuous data	Yes
Translating data	Inappropriate given predominantly quantitative data and the effectiveness focus of this review	No
Tabulation	Describe study characteristics and results. Will quality be assessed here? How? Predefined categories or just voicing methodological concerns that occur when reading the studies? Present these in text, tables, or both? Perhaps use the text descriptions to highlight any important aspects about *individual* studies that might not be apparent from the tables (issues across studies are more likely to fit into the next section on 'exploring relationships')	Yes
Vote-counting as a descriptive tool	Would be possible here if all data had been converted to odds ratios/relative risks/mean differences	Yes

Consequently, five of the six tools/techniques described in the guidance were applied to the synthesis and were carried out in the order described below.

Tabulating the data

It was decided that extracting data from the primary studies in tabular form might be the most natural starting point for the synthesis. This was done by using the same format as the Cochrane review's 'characteristics of included studies' table (participants, interventions, outcomes, notes) and adding further information, including country of origin, duration and provider of the intervention, number of participants in each group, context in which intervention was delivered, and results (see Table 4).

Study validity/quality is not addressed in detail in this section of the guidance. However, the Cochrane review did report some aspects of study validity (e.g. concealment of allocation) in the data extraction tables. It seemed sensible at this stage of the narrative synthesis (where the papers were being read in detail and some broad judgements about their content are starting to be made) to consider study quality. Consequently, a column including data on methods/quality was included in the table and structured comments were included regarding individual papers, based on Jadad et al's scale for evaluating RCTs.[53]

It became apparent that there were some discrepancies between the outcomes extracted for tabulation in the narrative synthesis and those in the Cochrane review. However, upon contacting the Cochrane review's authors, all these discrepancies could be explained by the inclusion of unpublished data or statistical adjustments for clustering. In these cases, to ensure comparability, data from the Cochrane review were used in the narrative synthesis.

At this stage, it became clear that the majority of studies were concerned with child safety, and that most included some measure of smoke alarm ownership/function as a main outcome. Only two studies[54, 55] looked at injuries as an outcome, but neither of these presented separate data on fire/smoke/burn related injuries.

Textual descriptions

It was not entirely clear what these might add to the preliminary synthesis over and above the information presented in the data extraction tables. Immediately after having constructed the data extraction tables, this seemed like an unnecessary duplication of effort, though it was considered that 'textual descriptions' might actually be useful for describing the interventions in more depth than can be usefully given in the tables. Consequently, the use of this technique was delayed until a later stage of the synthesis process.

Groupings and clusters

The presence of natural groups or clusters of studies was investigated, primarily to determine whether studies could be clustered according to the characteristics in the data-extraction tables (such as intervention, participants, setting, outcomes etc). The most obvious difference between studies in terms of the populations included is that all the studies deal with children and/or their families, with the exception of the Ploeg study that includes only participants aged 65+ years.[56] This study was therefore excluded from later comparisons. Secondly, studies could be clearly be grouped according to which of the four smoke alarm/ownership outcomes (specified a priori in the Cochrane review) they reported.

Developing a common rubric

As mentioned previously, data were only available for the four smoke alarm ownership/function outcomes. As these data were dichotomous, odds ratios and relative risks were calculated. Absolute risks differences and percentage smoke alarm ownership in the control group were also calculated for each smoke alarm ownership outcome and tabulated (an example for the 'final smoke alarm ownership' outcome is shown in table 5).

These tables showed that the effects of most interventions were generally fairly small for most smoke alarm ownership and function outcomes (absolute differences ranged from 0% to 12.4%). However, they generally favoured intervention over control (only two of the 10 studies that measured final smoke alarm ownership were negative for this outcome and one of the four studies reported a very small negative finding (absolute difference –0.1%) for 'smoke alarms acquired'.

Smoke alarm ownership in the control groups of each study was generally quite high, with one clear exception (Kelly et al),[54] 11%). As might be expected, there was greater range of odds ratios than corresponding relative risks for each outcome, as odds ratios are frequently more extreme (i.e. further from 1) than relative risks.

This approach proved a useful first step in comparing the effects observed across the included studies.

Table 2: Characteristics of included studies

Reference	Intervention	Participants	Setting/context	Outcomes	Results	Methods/quality	Other notes
Barone (1988)<br><br>USA	*Content:*<br>I: Usual safety education, plus slides and handouts on burn prevention, motor vehicle safety education and video; bath water thermometer; hot water gauge. (n=41)<br>C: Usual safety education (n= 38)<br><br>*Duration:*<br>4 x 2h weekly meetings.<br><br>*Delivered by:*<br>Unclear	Couples or individuals attending "Parenting the toddler" classes	Classes conducted at suburban hospital, family homes	Home inspection 6 months after class<br><br>1) Final smoke alarm ownership<br>2) Final functioning smoke alarms	1) Final smoke alarm ownership<br>I = 32/34<br>C = 26/29<br><br>2) Final functioning smoke alarms:<br>I = 39/41<br>C = 34/38<br><br>I = 32/34<br>C = 26/29<br><br>No significant difference between groups	Allocation by coin toss within paired classes<br><br>Outcome assessment not blinded<br><br>Withdrawals:<br>27% of parents attending randomised classes did not enrol in trial	
Clamp (1998)<br><br>UK	*Content:*<br>I: Safety advice, leaflets, discount safety devices for low income families (n=83 families)<br>C: Routine child health surveillance and routine consultations without intervention (n=82 families)<br><br>*Duration:*<br>Unclear<br><br>*Delivered by:*<br>Health visitors/practice nurses	Families of children <5 yrs on GP list	Delivered during child health surveillance consultations, opportunistically during other consultations, or the family was asked to make an appointment specifically for the intervention	Telephone/ mail survey 6 weeks after visit:<br>1) Smoke alarms acquired<br>2) Functioning smoke alarms acquired<br>3) Final smoke alarm ownership<br>4) Final functioning smoke alarms	1) Smoke alarms acquired:<br>I = 8/83<br>C = 0/82<br><br>2) Functioning smoke alarms acquired<br>I = 7/83<br>C = 4/82<br><br>3) Final smoke alarm ownership:<br>I: 82/83<br>C: 71/82<br><br>4) Final functioning smoke alarms:<br>I: 80/83, C: 71/82	Allocation by random numbers table numbered 1-165, the first 83 numbers on the list were allocated to the intervention group. Allocation was done by a researcher blinded to the number given to each family at the time of allocation<br><br>Outcome assessment not blinded<br><br>Withdrawals:<br>None	

Reference	Intervention	Participants	Setting/context	Outcomes	Results	Methods/quality	Other notes
Davis (1987)<br><br>USA	*Content:*<br>I: Fire safety lessons with workbook, demonstrations, teacher training, materials, take home materials for parents (n=439)<br>C: Usual lessons (n=418)<br><br>*Duration:*<br>6 x 1-hour lessons<br><br>*Delivered by:*<br>Teacher	Children in grade 4-6 classes	School	In school survey, immediately after class:<br><br>1) Final smoke alarm ownership	Final smoke alarm ownership:<br><br>I = 221/314<br>C = 195/299<br><br>I = 309/439<br>C = 272/418	Method of random allocation unclear<br><br>Outcome assessment not blinded<br><br>Withdrawals:<br>I = 1%<br>C = 0%	The study uses repeated hypothesis testing
Jenkins (1996)<br><br>Canada	*Content:*<br>I: Discharge teaching book about burn care and prevention; routine discharge teaching (n=62 families)<br>C: Routine discharge teaching (n=61 families)<br><br>*Duration:*<br>One session<br><br>*Delivered by:*<br>Physical therapist, occupational therapist or nurse	Families of children <17 years in burn unit	Delivered at discharge from burn unit	Interview in clinic at first follow-up visit (time since intervention unclear):<br><br>Final smoke alarm ownership	Final smoke alarm ownership:<br><br>I = 45/62<br>C = 46/61	Allocation by random numbers table read by independent person<br><br>Outcome assessment blinded<br><br>Withdrawals:<br>13% overall (unclear for each group)	48% of children in the study were of Native American Indian origin. Families were less likely to have safety devices, and less likely to speak English as a first language

Reference	Intervention	Participants	Setting/context	Outcomes	Results	Methods/quality	Other notes
Kelly (1987)<br><br>USA	*Content:*<br>I: Developmentally oriented child safety education, hazard assessment and handout at 6, 9 and 12-month well child visits. (n=55 families)<br>C: Usual 6, 9 and 12-month well child visits (n=54 families)<br><br>*Duration:*<br>Each visit approx 15 mins<br><br>*Delivered by:*<br>I = Principal investigator<br>C = primary caretaker (paediatric resident, fellow, faculty member, or nurse practitioner	Families of children aged 6 months seen for well child care	Family home	1) Final smoke alarm ownership (from home inspection, 1 month after 12-month visit)<br><br>2) Accidents and/or hospitalisations (from hospital record review)	1) Final smoke alarm ownership:<br>I = 8/55<br>C = 6/54<br>No significant difference between groups<br><br>2) ER/primary care visits for accidents:<br>I = 15/55<br>C = 11/54<br><br>Accidents requiring treatment:<br>I = 3/55<br>C = 4/54<br>Hospitalisations for accidents:<br>I = 1/55<br>C = 1/54	Method of random allocation unclear<br><br>Outcome assessment blinded.<br><br>Withdrawals:<br>I = 35%<br>C = 37%	
Kendrick (1999)<br><br>UK	*Content:*<br>I: Age specific advice, cheap safety equipment for low income families, home safety checks, first aid training. Checklists, information sheets and literature provided throughout (18 centres randomised, n=1124)<br>C: Usual care (no further description) (18 centres randomised, n=1028)<br><br>*Duration:*<br>Unclear.<br><br>*Delivered by:*<br>Health visitors and practice nurses	Children aged 3-12 months	Community	a) Record review of injuries<br><br>b) Postal survey of safety practices at 25 month follow-up:<br>1) Smoke alarms acquired<br>2) Functioning smoke alarms acquired<br>3) Final smoke alarm ownership<br>4) Final functioning smoke alarms	1) Smoke alarms acquired:<br>I = 15/274<br>C = 11/277<br><br>2) Functioning smoke alarms acquired:<br>I = 20/274<br>C = 14/277<br><br>3) Final smoke alarm ownership:<br>I = 254/274<br>C = 248/277<br><br>4) Final functioning smoke alarms:<br>I = 243/274<br>C = 241/277	Allocation by random numbers table by investigator blind to the identity of the practices<br><br>Outcomes assessment blinded<br><br>Withdrawals:<br>I = 67%<br>C = 64%	Not all participants received all aspects of the intervention

Reference	Intervention	Participants	Setting/context	Outcomes	Results	Methods/quality	Other notes
King (2001)<br><br>Canada	*Content:*<br>I: Home safety inspection and tailored education, safety device coupons; reinforcement (by telephone) at 4 and 8 months, plus a letter from the local project director (n=482 families)<br>C: Home safety inspection and general safety pamphlet only (n=469 families)<br><br>*Duration:*<br>Unclear<br><br>*Delivered by:*<br>“Home visitor”	Families of children aged <8 years hospitalised for injuries	Family home	Home inspection at 1 year follow-up:<br><br>1) Smoke alarms acquired<br>2) Functioning smoke alarms acquired<br>3) Final smoke alarm ownership<br>4) Final functioning smoke alarms	1) Smoke alarms acquired:<br>I = 14/476<br>C = 14/464<br><br>2) Functioning smoke alarms acquired:<br>I = 44/440<br>C = 36/435<br><br>3) Final smoke alarm ownership:<br>I = 460/479<br>C = 454/465<br><br>1.45 (0.94, 2.22), p=0.05.<br><br>4) Final functioning smoke alarms:<br>I = 412/459<br>C = 401/447<br><br>1.01 (0.79, 1.30)	Allocation by opening sealed, serially numbered, opaque envelopes<br><br>Outcome assessment blinded.<br><br>Withdrawals:<br>I = 20%<br>C = 18%	Though generally not given feedback after home safety inspection, control group families *were* informed if non-functioning smoke alarms were discovered

Reference	Intervention	Participants	Setting/context	Outcomes	Results	Methods/quality	Other notes
Mathews (1988)<br><br>USA	*Content:*<br>I: Home safety inspection, video, handouts, modelling re: safety and managing dangerous child behaviour; hot water thermometers; choke tube. (n=12 families)<br>C: Home visit with video, handouts, modelling on language simulation (n=12 families)<br><br>*Duration:*<br>Home visits 1.5 – 2 hours, intervention 45-60 mins<br><br>*Delivered by:*<br>Psychologist	Mothers of toddlers (12-14 months at first contact) from clinics, day care centres	Family home	Home inspection 2 weeks after home visit:<br><br>1) Smoke alarms acquired<br>2) Functioning smoke alarms acquired<br>3) Final smoke alarm ownership<br>4) Final functioning smoke alarms	1) Smoke alarms acquired:<br>I = 0/12<br>C = 0/12<br><br>2) Functioning smoke alarms acquired:<br>I = 0/12<br>C = 0/12<br><br>3) Final smoke alarm ownership:<br>Pre-test:<br>I = 10/12<br>C = 9/12<br><br>4) Final functioning smoke alarm ownership:<br>I = 6/12<br>C = 6/12<br><br>There were no significant differences between groups or trials on these outcomes	First eight participants allocated in odd-even manner, remainder using open random numbers table<br><br>Blinding unclear<br><br>Withdrawals:<br>8% in total	

Reference	Intervention	Participants	Setting/context	Outcomes	Results	Methods/quality	Other notes
Ploeg (1994)<br><br>Canada	*Content:*<br>I: Safety behaviour promotion A safety checklist developed from the injury prevention literature, used with clients to discuss personal, home and community safety and to address strategies to improve safety. (n=148)<br>C: Influenza immunisation promotion (n=211)<br><br>*Duration:*<br>One visit Duration unclear<br><br>*Delivered by:*<br>Public health nurses	English speaking public health clients aged 65 or over<br><br>Mean age 77.2 years, 67% female	Delivered during a visit to the client's home	Telephone survey after 2-3 months:<br><br>Smoke alarms acquired	Smoke alarms acquired:<br>I = 3/146<br>C = 1/197	Allocation by random numbers table read by independent person<br><br>Outcome assessment blinded<br><br>Withdrawals:<br>I = 1%<br>C = 7%	
Thomas (1984)<br><br>USA	*Content:*<br>I: Well-baby classes with standard safety information plus burn prevention education lecture, pamphlet, handouts and discount coupon for smoke alarm purchase (9 classes: n=29)<br>C: Well-baby classes with standard safety information (6 classes: n=26)<br><br>*Duration:*<br>I/C: 1 x 90min session<br><br>*Delivered by:*<br>Paediatric nurse practitioners	Volunteer parents of infants enrolled with a single HMO<br><br>No further information provided	Hospital? (conference room)	Home inspection 4-6 weeks after class:<br><br>Final smoke alarm ownership	Final smoke alarm ownership:<br>I = 27/28<br>C = 21/25	Randomised using coin toss.<br><br>Blinding unclear<br><br>No withdrawals mentioned	Smoke alarm ownership was very high in both groups (actual numbers not given for C group)

Reference	Intervention	Participants	Setting/context	Outcomes	Results	Methods/quality	Other notes
Williams (1988)<br><br>USA	*Content:*<br>I: Usual safety education plus 1 hour lecture, handouts on burn prevention; motor vehicle safety education and video (n=40).<br>C: Usual safety education plus 1 hour lecture, handouts on infant stimulation and feeding (n=35)<br><br>*Duration:*<br>Unclear<br><br>*Delivered by:*<br>"Trainer"	New mothers identified while attending prenatal classes	Unclear	Home inspection 4-70 weeks after delivery:<br><br>1) Final smoke alarm ownership	Outcome data not available.<br><br>The authors state that there was no difference between I and C groups, with both groups showing usage rates for smoke alarms of over 77%	Allocation by random numbers table by independent statistician<br><br>Outcome assessment not blinded<br><br>Withdrawals:<br>55% of women attending randomised classes did not enrol in trial	

I = Intervention group
C = Control group

Table 3: Final smoke alarm ownership (common rubric and vote count)

Reference	Absolute difference (%)	Relative risk (95% CI)	Odds ratio (95% CI)	Vote count RR	Vote count OR	% smoke alarm ownership in control group
Barone (1988)	4.5	1.05 (0.90, 1.22)	1.85 (0.29, 11.89)			90
Clamp (1998)	12.2	1.14 (1.04, 1.25)	12.7 (1.6, 100.85)	✓	✓	87
Davis (1987)	5.2	1.08 (0.97, 1.20)	1.27 (0.9, 1.78)			65
Jenkins (1996)	-2.8	0.96 (0.78, 1.19)	0.86 (0.39, 1.93)			75
Kelly (1987)	3.4	1.31 (0.49, 3.52)	1.36 (0.44, 4.23)			11
Kendrick (1999)	3.2	1.04 (0.98, 1.09)	1.49 (0.82, 2.7)			90
King (2001)	-1.6	0.98 (0.96, 1.01)	0.59 (0.28, 1.25)			98
Mathews (1988)	8.3	1.11 (0.74, 1.68)	1.67 (0.22, 12.35)			75
Thomas (1984)	12.4	1.15 (0.95, 1.38)	5.14 (0.53, 49.5)			84
Williams (1988)	No stats	No stats	No stats	No stats	No stats	>77

Key to table colour coding

- Significantly favours intervention
- Trend towards intervention
- No difference
- Trend towards control
- Significantly favours control

Vote counting as a descriptive tool

Tables showing two approaches to vote counting were developed: (i) only using ticks where the effect of the intervention was positive and statistically significant; (ii) using colours (superimposed on the rows of the table) to grade both the direction and statistical significance of each outcome (see table 5 for an example showing the 'final smoke alarm ownership' outcome).

In terms of the vote-count there were no differences between the relative risks and odds ratios calculated previously. The study by Williams[57] reported that there was no statistically significant difference between the experimental and control groups but did not provide data to calculate the measures in this table. For the subsequent steps, the relative risk and the more "informative" (colour coded) vote count were both used.

The vote-count supported the observations previously made by looking across the absolute risk values. Where several studies report the same outcome, the majority of these studies show a tendency to favour the intervention over control, though the relative risk is usually small. Only one study reported any statistically significant differences between intervention and control groups (Clamp reported statistically significant positive effects of intervention on final smoke alarm ownership and final functioning smoke alarms).[58]

In this case, the colour-coded descriptive vote-count allows the reader to see the outcome data as either a simple vote-count or as a statistical value, depending upon the 'focus' they adopt when examining the outcome table.

4.4 Exploring relationships within and between studies

Tools/techniques described in this section of the guidance are described in the table below.

Table 4: Selection of tools and techniques for exploring relationships between studies

**Name of tool/technique**	**Thoughts/ideas/comments in relation to current synthesis**	**Should this tool/technique be applied here?**
Moderator variables and subgroup analyses	Most likely sources of potential moderator variables are likely to be variations in intervention, population or possibly setting	Yes
Idea webbing/conceptual mapping	This may help structure the investigation of moderator variables	Yes
Conceptual triangulation	This approach would be more appropriate to a synthesis of implementation studies, in which more qualitative information is likely to be available and there is greater scope for model development	No
Reciprocal/refutational translation	Insufficient qualitative evidence in this review	No
Qualitative case descriptions	This appears to be essentially the same as the 'textual descriptions' described earlier. However, here the approach is presented in the context of investigating differences between, rather than simply describing, the studies. Might be worthwhile to revisit the studies and extract detailed data from them, with an eye to any potential moderator variables	Yes
Visual representation of relationship between study characteristics and results	This is possible given the quantitative data available for each study	Yes
Investigator and methodological triangulation	More applicable to qualitative studies. As all studies here were RCTs, there should not be any systematic difference in results between authors from different disciplines (if there was, bias would be a very serious concern). Data on the disciplinary perspective/expertise of investigators was not available for all studies	No

The four main tools and techniques for exploring relationships within and between studies were conducted in the order described below.

Moderator variables and subgroup analyses

It would be useful to know any variables that might moderate the main effects being examined by the review. Two further types of table were drawn up to help investigate whether there were any clear moderators of effect. The first table was constructed to show the various components that make up the intervention for each study and the overlap between the different interventions in terms of these components (table 5).

The table indicates that there is little overlap between the studies in terms of the specific components employed within the interventions they evaluate. Seven of the ten studies concerned with children and/or their families used handouts and four used 'burn education', money-off coupons or discounted devices and home safety inspections. However, this lack of overlap is possibly due to the fact that studies were, on the whole, very poorly described. Even when sufficient information was reported allow extraction, there was still variation in the terms and definitions used by different authors, making direct comparisons even more difficult.

The second set of tables is an adaptation of the outcomes/vote count table, with further information taken from the data extraction table and the intervention components table described above (table 6 gives an example for the 'final smoke alarm ownership' outcome). Intervention, population and setting columns were included to identify potential subgroups/moderators. These are described as briefly as possible (1-5 words) to allow visual comparison across the table. The description of the intervention is broken into 3 separate cells to facilitate such visual comparisons for the complex interventions.

Looking at the outcome of 'final smoke alarm ownership' (for which the majority of studies provide data), four studies stand out from the majority of positive but statistically non-significant findings: Williams (no difference),[57] Clamp (significantly positive),[58] Jenkins and King (both non-significantly negative).[59, 60] Williams reports that "there were no differences between experimental and control groups",[57] though whether this means there was truly no difference between the groups or that any observed differences were not statistically significant is unclear. Either way, it is difficult to determine why the studied intervention had little or no effect based on this one study alone. The intervention studied by Clamp included safety advice, discounted safety devices and handouts and resulted in a significant increase in final smoke alarm ownership and function.[58] However, these particular intervention components were common to other studies that differed from Clamp's study in terms of both magnitude and statistical significance of effect. The two negative studies on the ownership outcome (Jenkins and King) evaluate two different interventional approaches.[59, 60] However, these studies do share a common characteristic that is not present in the 'positive' studies: the intervention was delivered to the families of children that had been previously hospitalised for an injury.

Qualitative case reports/textual descriptions

The two 'tools' 'textual descriptions' and 'qualitative case descriptions' would seem to be very similar. It was decided that writing a short summary of each study at this stage of the synthesis (i.e. having already organised, described and examined them) would provide an opportunity to check the previous stages for accuracy, and allow the reviewer to draw out in detail any aspects of individual studies that may not have seemed relevant at the start of the synthesis, but have become of interest during the subsequent stages of describing and exploring the study data. These summaries were structured such that they provided details of the setting, participants, intervention, comparison, and outcomes, along with any other factors of interest (for an example of one such description see Box 1).

Table 5: Table showing various components of the evaluated interventions

	Burn education	Slides	Handouts	Safety advice	Discount devices /coupons	First aid training	Home safety inspection	Tailored education	Reinforcement	Video	Modelling	Free thermometer /choke tube	School fire safety lessons	Child safety education
Barone (1988)	✓	✓	✓											
Clamp (1998)			✓	✓	✓									
Davis (1987)			✓										✓	
Jenkins (1996)	✓													
Kelly (1987)			✓				✓							✓
Kendrick (1999)				✓	✓	✓	✓							
King (2001)					✓		✓	✓	✓					
Mathews (1988)			✓				✓			✓	✓	✓		
Thomas (1984)	✓		✓		✓									
Williams (1988)	✓		✓	✓						✓				

*Above studies relate to children/families. Ploeg (included only ptps aged >65yrs)

Table 6: Final smoke alarm ownership (potential moderator variables)

Reference	Intervention			Population	Setting	Absolute difference (%)	% smoke alarm ownership in control group
Barone (1988)	Burn education	Slides	Handouts	Parents of toddlers	Hospital, family home	4.5	90
Clamp (1998)	Safety advice	Discount devices	Handouts	Parents of children <5yrs	Family home, other	12.2	87
Davis (1987)	Fire safety lessons		Take home material for parents.	Children	School	5.2	65
Jenkins (1996)	Discharge teaching book on burn care/prevention			Children <17yrs	Hospital burn unit	-2.8	75
Kelly (1987)	Child safety education		Home safety inspection<br>Handouts	Families of babies <6mths	Family home	3.4	11
Kendrick (1999)	Safety advice<br>First aid training	Discount devices	Home safety inspection	Families of babies 3-12mths	Community	3.2	90
King (2001)	Tailored education<br>Reinforcement	Discount coupons	Home safety inspection	Families of hospitalized children <8yrs	Family home	-1.6	98
Mathews (1988)	Video<br>Modeling re safety	Free thermometers and choke tube.	Home safety inspection<br>Handouts	Mothers of toddlers (12-18mths)	Family home	8.3	75
Thomas (1984)	Well-baby classes plus burn prevention education lecture.	Discount smoke alarm coupon	Handouts<br>Pamphlet	Parents of infants	Hospital(?)	12.4	84
Williams (1988)	Burn prevention lecture		Handouts	Pregnant women (last trimester)	Unclear	No stats	>77

Key to colour coding:

- Significantly favours intervention
- Trend towards intervention
- No difference
- Trend towards control
- Significantly favours control

A number of questions arose from the process of writing these summaries:

- Does the immediate on-site availability of smoke alarms in the intervention setting increase uptake?
- Are lower income families more likely than higher income families to respond to interventions incorporating discounted smoke alarms?
- Does having experienced a child injury prior to intervention increase uptake of the recommendations given in the intervention?
- Do interventions that focus on burn injuries/fire prevention have different effects to interventions that relate to safety more generally?
- Does advice being age-specific alter outcomes? Would advice regarding fire safety always be the same, independent of child age?
- Does attrition have an effect?
- Is length of follow-up an important factor?
- Is sample size important? Studies may be powered to detect differences on other outcomes.
- Several studies attribute any lack of effect to the fact that an active effort is required to install smoke alarms. Is there a relationship between intervention effectiveness and amount of active effort required?

Box 1 – Example of textual descriptions/qualitative case descriptions of included studies

Barone (1988)

Setting: US suburban hospital.

Participants: Individuals or couples attending a continuing-education series on “Parenting the Toddler”. Couples were predominantly of middle- and upper-middle class socioeconomic status and generally well educated.

Intervention: Parenting information, with specific information and materials on burn prevention and child restraints. Included a slide presentation on falls, strangulations, drownings, poisonings, and fire hazards, plus additional slides on the hazard of hot tap water, use of smoke detectors and the advantages of child car seats. 4 weekly sessions, each of 2 hours duration. 41 participants.

Comparison: Parenting information, with general child safety information. Included a slide presentation on falls, strangulations, drownings, poisonings, and fire hazards. 38 participants.

Outcomes: A researcher inspecting participants’ homes looked for and tested any smoke alarms, 6 months after the classes.

Other:

- The protocol for this intervention is very similar to that described by Williams (both are from the same University in the same year).
- The author suggests that the very high rate of smoke alarm ownership might be due to previous health promotion efforts.
- The author also suggests that it would have been possible for participants in the control group to be ‘warned’ in advance what the researchers were looking for and testing during home inspections by other participants whose homes had already been inspected.

This suggests that the production of summaries can be a helpful prelude to identifying and assessing impact of moderator variables, building on data extraction and developing conceptual models.

Developing conceptual models/idea webbing/concept mapping

These three tools/techniques are also very similar although the implementation narrative synthesis illustrates differences between them. The aim of using these techniques in this example was to make transparent the logic behind the subgroup analyses/investigation of moderator variables (see figure 5). In working through this process it became apparent that it also incorporates aspects of grouping and clustering. The resulting figure is also in part a way to link the previously described processes and the resulting issues/ideas together in order to structure the synthesis. It represents the product of

a process whereby variables or patterns are identified in one of the previously described tables or documents and then re-examined from the viewpoint of the remaining tables/documents. For example, the characteristic most fully explored in the figure is that of the included study population, as described in the table of potential moderator variables and in the textual descriptions. Studies of children/families were grouped by age of the included children according to the moderator tables. Within these groups, further participant variables such as socioeconomic status were identified from the textual descriptions.

The ‘outcomes’ and ‘quality’ nodes are connected to one another via ‘loss to follow-up’. The withdrawal rates vary substantially across this group of studies, from 0% to 67%. Where high dropout rates are discussed in these studies, it is attributed non-attendance over time or unavailability of participants at final follow-up.

Though identified as potential moderators, no clear or consistent effect on smoke alarm ownership could be seen across studies for intervention variables such as the use of home inspections or free/discounted devices, or for fire/burn-specific education alone versus general safety information that incorporates fire/burn material.

Initially idea webbing was very useful approach to guiding the synthesis. However, the ad hoc use of the approach led to a natural impulse to seek out any association, no matter how spurious. Given this it may be better to use these types of approaches early on in the synthesis (or even protocol development) process to identify *a priori* the characteristics to be investigated and to structure the synthesis before seeing the data itself.

Alternatively, it might be useful to employ this approach at both points in the review process (protocol development and exploring relationships in the final synthesis), placing more “weight” on investigations from the *a priori* idea web (i.e. using it to help develop conclusions about effects and moderators), and using the idea web constructed after interrogating the data purely for suggesting areas in which further research might be worthwhile.

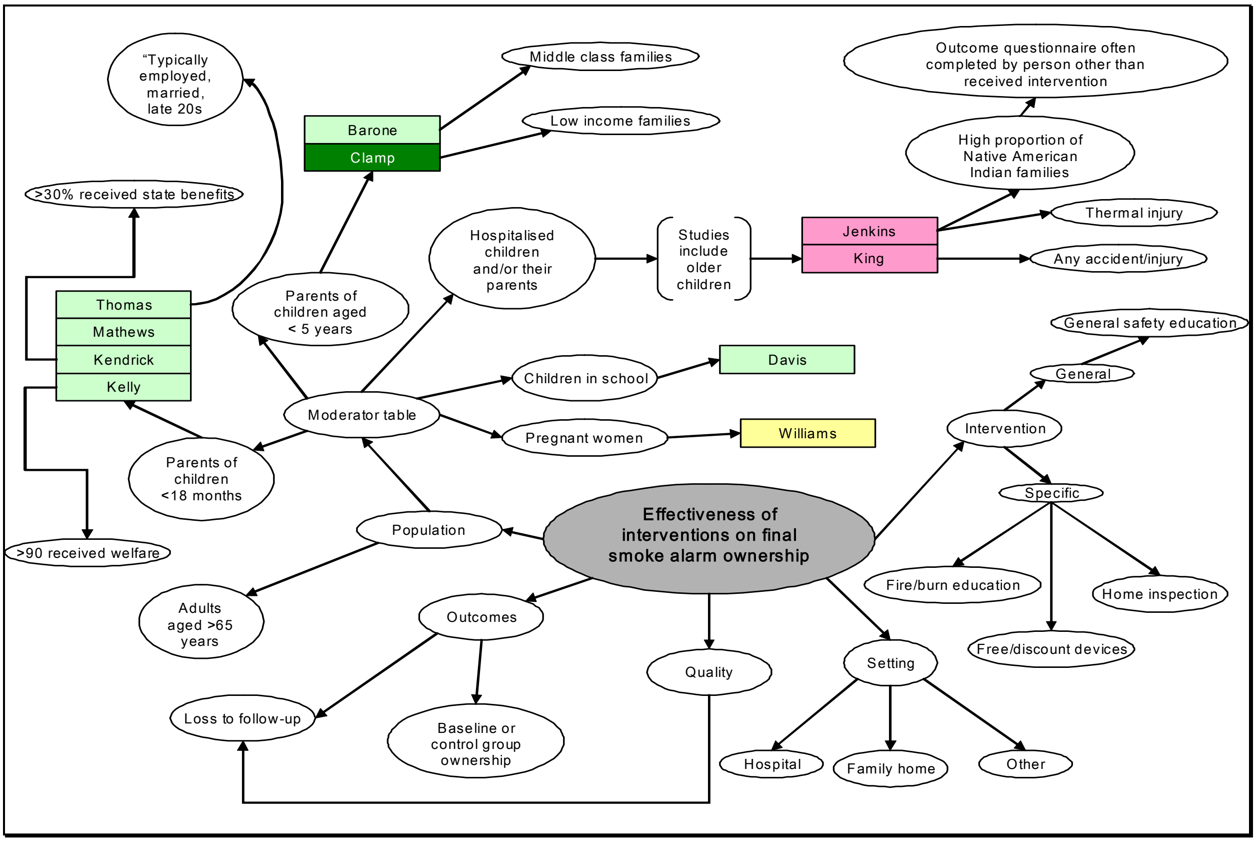

Figure 5: Conceptual mapping/idea webbing

Visual representation of relationships between study characteristics and results

Funnel plots to examine the relationship between study sample size/variance and effect size were constructed by plotting relative risk against standard error (see figure 6). Due to the small number of studies reporting data on the outcomes of interest, these proved to be largely uninformative. The plot for 'final smoke alarm ownership' shows that the study with the lowest precision is that with the most strongly positive effect, but this alone does not provide strong evidence for publication bias.

These proved unhelpful but may be more useful in larger reviews where enough quantitative data are reported to allow a visual display. However this may not be the case for many systematic reviews of social interventions.

Forest plots showing the point estimates and 95% confidence intervals for each study for each of the main outcomes (but without a pooled estimate) were also drawn, as suggested in the guidance (figure 7). These provide a clear visual representation of the relative risks and associated 95% confidence intervals previously presented as in table 5.

Figure 6: Funnel plots showing standard error versus relative risk for each outcome

a)

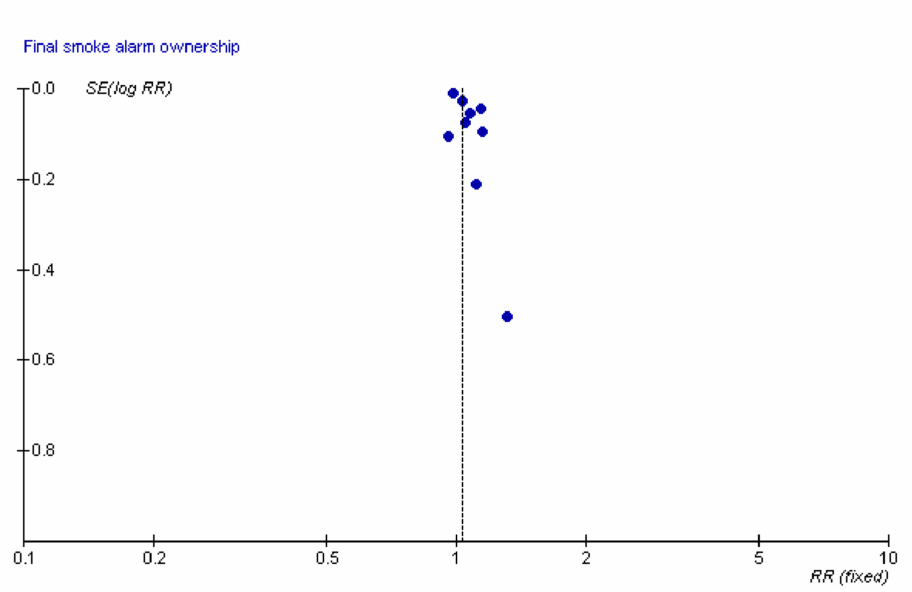

b)

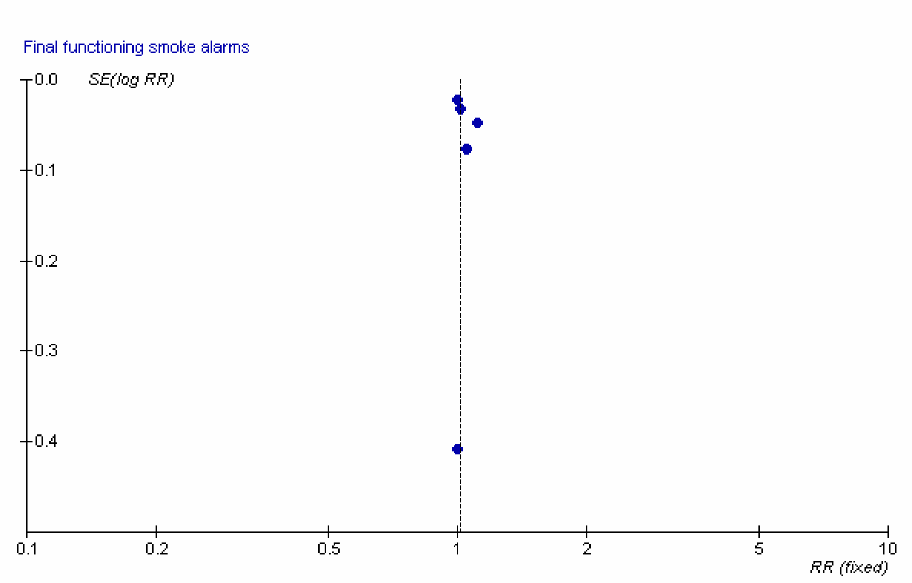

c)

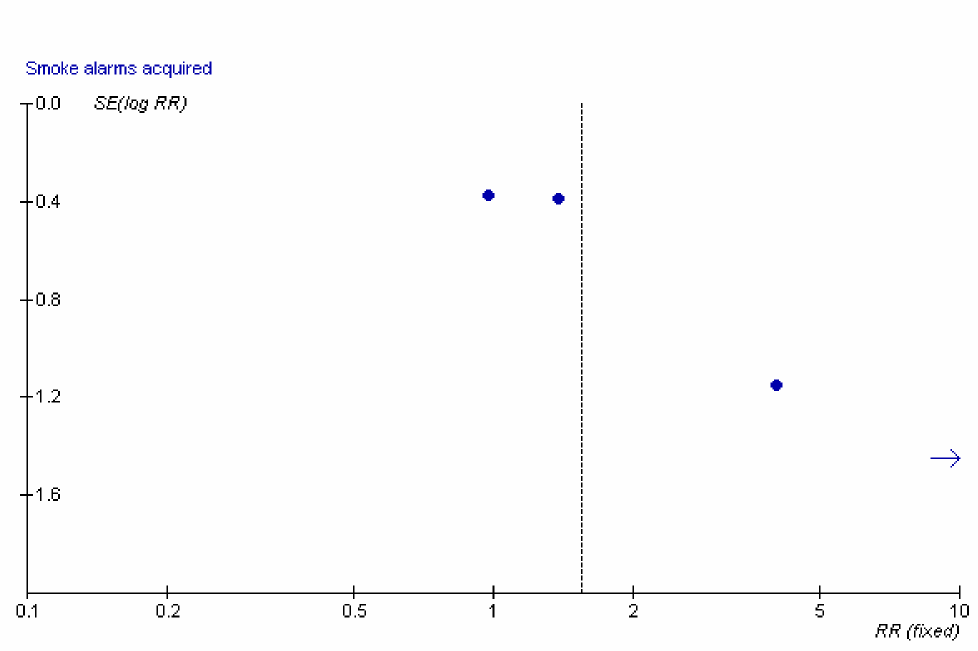
Smoke alarms acquired
0.0
SE(log RR)
0.4
0.8
1.2
1.6
0.1
0.2
0.5
1
2
5
10
RR (fixed)

d)

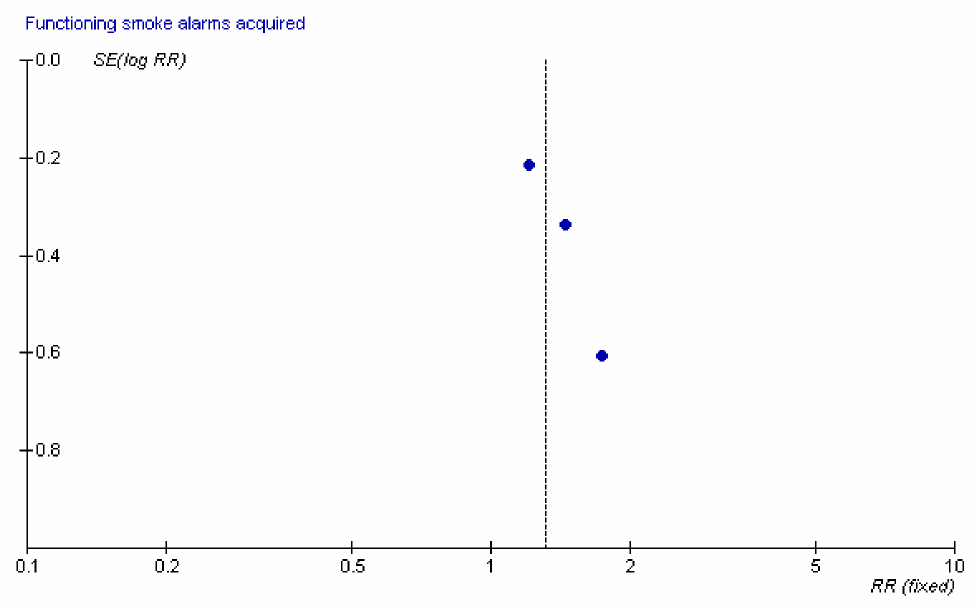
Functioning smoke alarms acquired
0.0
SE(log RR)
0.2
0.4
0.6
0.8
0.1
0.2
0.5
1
2
5
10
RR (fixed)

Figure 7: Forest plots (without pooled data) for each outcome

a)

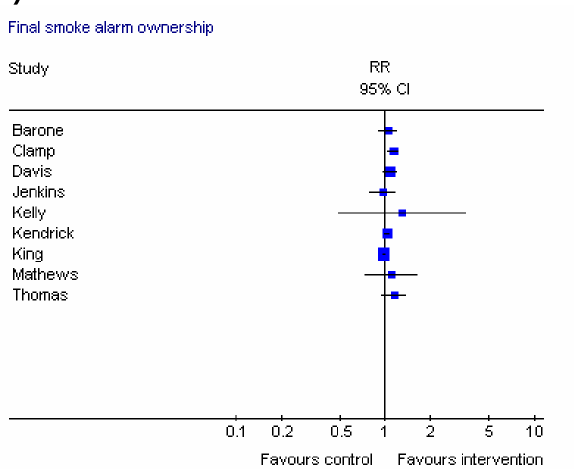

b)

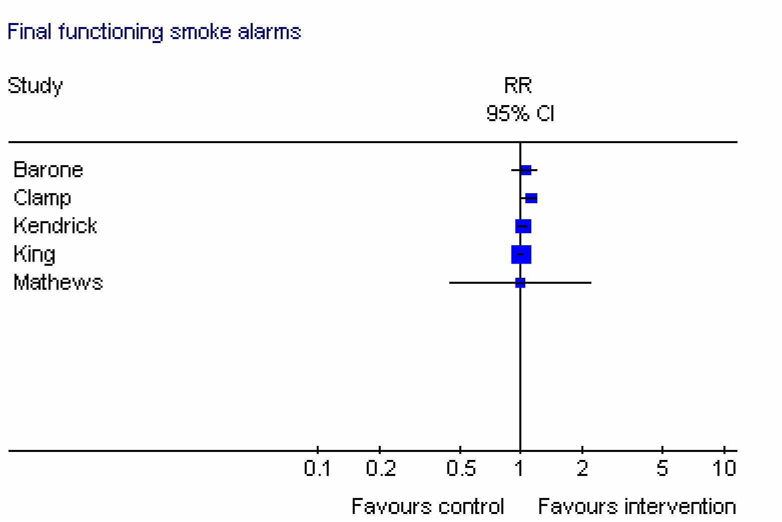

c)

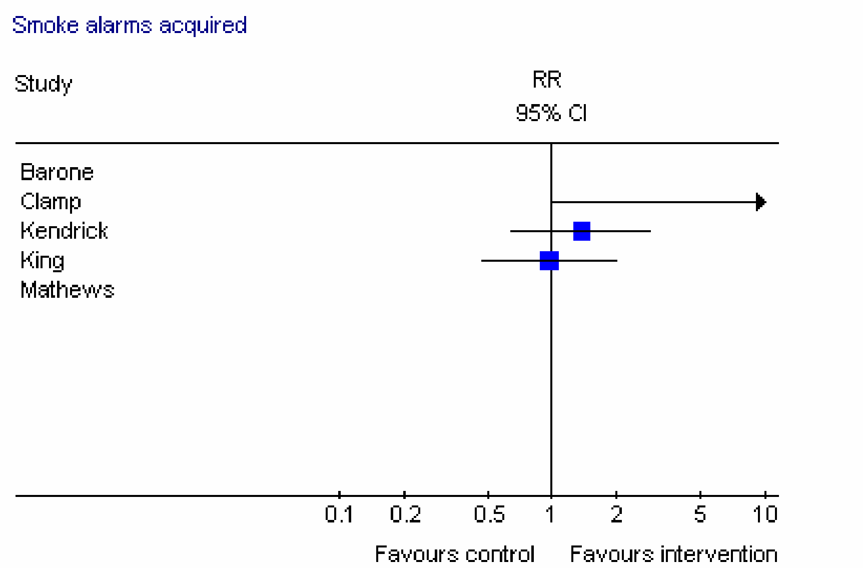
Smoke alarms acquired
Study
RR
95% CI
Barone
Clamp
Kendrick
King
Mathews
0.1
0.2
0.5
1
2
5
10
Favours control
Favours intervention

d)

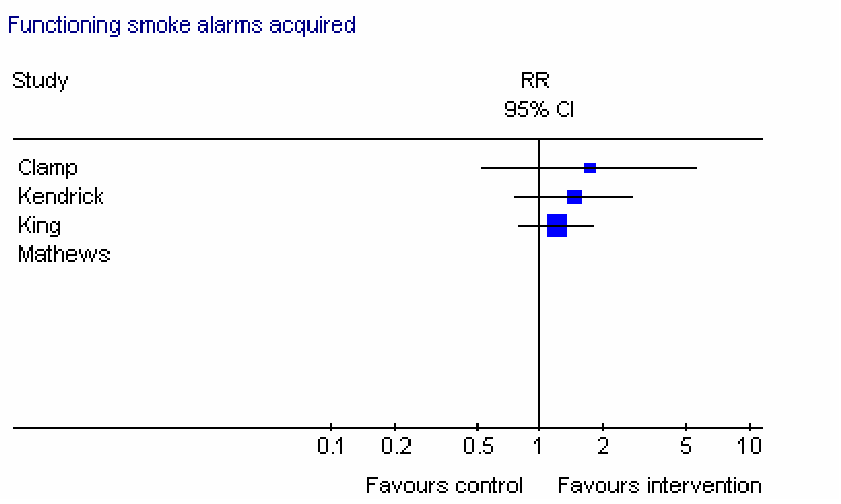
Functioning smoke alarms acquired
Study
RR
95% CI
Clamp
Kendrick
King
Mathews
0.1
0.2
0.5
1
2
5
10
Favours control
Favours intervention

4.5 Assessing the robustness of the synthesis

Tools and techniques described in this section are presented in the table below.

Table 7. Selection of tools and techniques to assess the robustness of the synthesis

Name of tool/technique	Thoughts/ideas/comments in relation to current synthesis	Should this tool/technique be applied here?
Best evidence synthesis	Not really appropriate since this technique is primarily concerned with the selection of studies, and all studies in this synthesis are RCTs	No.
Use of validity assessment (EPPI-centre approach, CDC approach)	EPPI approach may be possible, using internal validity data presented in the summary tables. CDC approach needs further clarification before it could be applied (e.g. what is a "sufficient" effect size?)	Yes (EPPI).<br><br>No (CDC).
Checking the synthesis with authors of primary studies.	Not possible given the time available for this synthesis.	No.
Reflecting critically on the synthesis process	Although partly done throughout this process, it might be useful to have a dedicated section discussing issues that arose from the synthesis.	Yes.

Strength of evidence (EPPI approach)

The guidance states that "four criteria are used to appraise each study: (1) the study's methodological soundness, (2) the appropriateness of the study design to answering the review question, (3) the study relevance, and (4) an assessment of the overall weight of evidence which the study provides. The first three criteria contribute to the assessment of (4) study "weight". These are described elsewhere by EPPI review authors as (1) Trustworthiness, (2) Appropriateness, (3) Relevance, and (4) Overall weight. An attempt was made to tabulate these characteristics for the studies included here (table 8), with criterion (1) based upon the validity evaluations in the first data extraction table (as these are derived from the Jadad scale, scores of 3-5 are considered 'high' quality. In this example, a score of 2 was described as 'medium' and a score of 0 or 1 as 'low').

Of the ten studies of children or their families, three received an overall 'high' weight, five were classified as 'medium' and two were given an overall weight of 'low'. These 'overall weights' corresponded exactly to the 'trustworthiness' scores that relate to internal validity. This is because there was little to distinguish between the studies in terms of appropriateness (all were RCTs – a design appropriate to this kind of evaluative research) and relevance (studies were selected for relevance early in the review by the application of inclusion criteria). The only study that was not considered 'highly' relevant in its focus was by Davis, as this was delivered to exclusively to schoolchildren, whereas other studies involved parents in the intervention.

It is possible that these 'overall weights' overemphasise the differences between the included studies. All of the studies scored 1, 2 or 3 on the Jadad scale and were consequently labelled 'low', 'medium' and 'high' respectively. All of the studies described themselves as RCTs, and (partly because of the nature of the intervention) none were double-blind or used an indistinguishable control intervention. Therefore, overall study weighting was dictated solely by whether the studies included descriptions of allocation concealment and/or withdrawals.

Three studies received an overall weighting of "high" (Clamp, Kendrick and King).[55, 58, 60] However, these were conducted in different settings and, for final smoke alarm ownership, reported differing results from one another. Consequently, this quality assessment approach does not greatly impact on the current synthesis, though could prove more useful in syntheses where there is greater variation in the quality of the studies being synthesised.

Table 8: Weighting of studies by quality, according to four criteria

Study	A Trustworthiness	B Appropriateness	C Relevance	D Overall weight
Barone (1988)	Medium	High	High	Medium
Clamp (1998)	High	High	High	High
Davis (1987)	Medium	High	Medium	Medium
Jenkins (1996)	Medium	High	High	Medium
Kelly (1987)	Medium	High	High	Medium
Kendrick (1999)	High	High	High	High
King (2001)	High	High	High	High
Mathews (1988)	Low	High	High	Low
Thomas (1984)	Low	High	High	Low
Williams (1988)	Medium	High	High	Medium

4.6 Reflecting critically on the synthesis process

Methodology of the synthesis used

There were some limitations to the approach taken in this synthesis, relating to the potential for bias. For example, the selection and arrangement of intervention components included in the moderator table was to some extent subjective. Similarly, the themes emerging from the textual descriptions that seemed most important were chosen at least partly subjectively. This is another argument for 'down-weighting' conclusions based on moderators only identified through extensive examination of the primary studies.

In the case of this particular synthesis, only RCTs were included. Subsequently, there was less methodological heterogeneity than in many narrative syntheses. This precluded the use of several techniques (although it is unlikely that any synthesis would be able to make use of *all* the tools and techniques described in the guidance). As all the studies were RCTs, the techniques that were appropriate were often variations on those used when undertaking a meta-analysis. It also meant that the variation in quality between studies was relatively small and difficult to incorporate usefully into the synthesis.

4.7 Conclusions

Interventions that provide safety information directly to families of young children appear in general to have a small beneficial effect on smoke alarm ownership and function. No conclusions can be made about the effect of such interventions in terms of fire-related injury or burn prevention, as these outcomes were not reported separately. It is unclear from the synthesis of RCTs presented here how specific fire-related safety education compares with general safety advice. Neither is there a clear relationship between the incorporation of home inspections or discount devices/coupons and the effect of interventions on smoke alarm ownership/function.

However, examination of the studies indicated several implications for the conduct of research in this area:

Implications for research on smoke alarms

- Future RCTs of similar interventions should measure relevant fire-related injury and burn outcomes after an appropriately long follow-up, preferably from hospital record review or similar method that reduces the potential for bias and attrition inherent in the questionnaire methods employed in several of the currently published RCTs.
- Any future studies should provide full and detailed descriptions of the intervention being evaluated and each of its components.
- Theory should be incorporated into the design and evaluation of any such intervention. Those designing evaluations of this type of intervention should consider the causal pathways between providing the intervention and the outcomes, and the barriers to its

adoption, and ensure that data is provided on each of the steps (or events) in the pathway.

- Randomised studies should take into account confounding due to concurrent community-wide initiatives and legislation to increase fire injury awareness and smoke alarm ownership.
- The rates of smoke alarm ownership at baseline might be investigated as a potential variable that influences intervention effectiveness within the target population.
- The only studies with negative findings in this synthesis were those in which participants were children, or the families of children that had been hospitalised for an injury. Whether this was a chance finding or indicative of a true lack of effect for these interventions in families of previously injured children may be of interest.

Comparison of narrative synthesis and meta-analysis

The Cochrane review,[51] based on the meta-analysis of the same group of RCTs, reached very similar broad conclusions to the narrative synthesis. The Cochrane authors reported that fire-related injury outcomes were not available and the main meta-analyses of RCTs showed that "smoke alarm ownership at follow-up appeared somewhat more likely in the intervention group (OR = 1.26; 95% C.I., 0.87 to 1.82). Similarly modest positive, statistically non significant effects on functioning smoke alarms, and on new acquisitions of smoke alarms and functioning smoke alarms, were found". They summarised that there were "only modest potential benefits from education to promote smoke alarms".

As in the narrative synthesis, the apparent lack of effect of intervention in the two trials involving families of injured children was noted in the Cochrane review. The Cochrane authors state that "exclusion of these trials from the meta-analyses results in a stronger, statistically significant intervention effect on alarm ownership (OR = 1.43; 95% C.I., 1.07 to 1.90) and other alarm outcomes". In the narrative synthesis, this was considered an area of potential interest for future research. The Cochrane authors suggest in the discussion section of their review, "Having an injured child may lead to safety behaviour changes so large that they obscure any safety education effects", but they do not mention this as one of their implications for future research.

In addition, the Cochrane review concluded that smoke alarms delivered as part of child health surveillance may be more effective. The effects on final smoke alarm ownership were statistically significant (OR = 1.96; 95% C.I., 1.03 to 3.72), with strong, non-significant effects on the other ownership and function outcomes. The authors state that these subgroup analyses were based on few trials and were heavily influenced by a single trial.(Kendrick)[55]

The relationship between offering discount devices/coupons and the effect of interventions on smoke alarm ownership/function was not obvious in the narrative synthesis. The results of a subgroup meta-analysis suggested that offering discounted alarms had a modestly stronger effect on smoke alarm ownership (OR = 1.83; 95% C.I., 0.63 to 5.28) than did education alone, but the trial results were significantly heterogeneous (p=0.015). Another subgroup meta-analysis indicated that the removal of the one study in which a research assistant delivered the intervention[60] resulted in a stronger positive effect of intervention on three of the reported outcomes.

The Cochrane authors concluded that the quality of the available evidence is limited, with sensitivity analyses showing that pooled trials with blinded outcomes assessment indicated little apparent effect on ownership or function, whereas unblended studies indicated strong effects.

Recommendations derived from the meta-analyses were similar to those in the narrative synthesis: "Further trials to evaluate the effect of smoke alarm promotion as part of child health surveillance in primary care… should assess their impact on fire-related injuries, using adequate allocation concealment and blinded outcomes assessment". These recommendations did not stretch to improvements in outcome measurement, description of interventions, use of theory in designing interventions, or adjusting for potential confounding from concurrent fire safety initiatives/policies, as they did in the narrative synthesis.

On the whole, the findings of the narrative synthesis and the meta-analyses were very similar. However, the differences mentioned above appear to be attributable to two main factors: the

possibility of undertaking sensitivity and subgroup pooled analyses in the meta-analyses and the close scrutiny of studies undertaken in the narrative synthesis. Consequently, conclusions about the impact of moderators on effect appeared to be ‘firmer’ when derived from the meta-analysis than from narrative synthesis, whereas implications for future research appeared to be more extensive and detailed when derived from the narrative synthesis. However, the Cochrane review authors mention caveats in relation to some of the ‘additional’ findings derived from subgroup analyses (e.g. that the apparent increase in effect attributable to offering discounted alarms was based on a meta-analysis of highly heterogeneous studies). Meta-analysis allowed the authors of the Cochrane review to observe the impact of specific aspects of study validity (allocation of concealment and blinded outcome measurement) on results. In the narrative synthesis, validity was considered more broadly and showed no obvious correlation with study results. Although the differences in the conclusions of the two syntheses were relatively minor, it is unclear whether it would be possible to eradicate them altogether, considering that by definition narrative synthesis precludes statistical pooling.

The process of comparing these two syntheses has highlighted the important contribution that the guidance makes to increasing the transparency of the narrative synthesis approach. As a reader, it was possible to check the conclusions derived from the narrative synthesis by examining the synthesis itself and the associated tables and figures, much as it is possible to examine and interpret data presented in a series of forest plots.

CHAPTER 5: *APPLYING THE GUIDANCE 2:* A NARRATIVE SYNTHESIS OF STUDIES INFORMING THE IMPLEMENTATION OF DOMESTIC SMOKE ALARM PROMOTION INTERVENTIONS.

5.1 Introduction

This chapter, like the previous one, provides a practical example of a narrative synthesis. In this case, however, the focus is on the synthesis of evidence on factors influencing the ***implementation of interventions*** rather than effectiveness. The specific aims of the chapter are to:

- Illustrate in practical terms the decision making processes involved in the application of the guidance to a specific narrative synthesis
- Identify factors that should inform choices about the use of particular tools and techniques in the context of a specific synthesis
- Provide examples of how particular tools and techniques can be used in the synthesis of evidence on the implementation of specific interventions
- Demonstrate the type of outcomes achieved by a narrative synthesis
- Comment on the way in which the present synthesis compares with the earlier work on which we drew

The evidence on implementation synthesised in this chapter is drawn from an earlier, exploratory review of evidence on the implementation of interventions aiming to promote the use and functioning of domestic smoke alarms and broader community based injury prevention interventions.[61] The focus of the narrative synthesis reported here has been restricted to domestic smoke alarm interventions. A new search was conducted to update the studies identified for the original review and a purposive sample of seven papers judged by the review team to provide relatively rich data on factors influencing the implementation of the interventions was selected for inclusion in the narrative synthesis. Details of the seven papers, and the interventions reported on, are shown below in Table 9.

Table 9. The seven papers

Paper/report authors	Location of trial/initiative	Type of trial/ initiative	Type of intervention
1. Campbell DeLong Resources Inc. (2003)[62]	Oregon, USA	Non RCT	Fire safety awareness campaign (aimed primarily at landlords)
2. Camit, M. (1998)[63]	New South Wales, Australia	Non RCT	Provision of discounted smoke alarms, plus written fire safety information and alarm demonstration
3. Camit, M. (2002)[64]	New South Wales, Australia	Non RCT	Provision of discounted smoke alarms, plus written fire safety information and alarm demonstration
4. DiGuiseppi, C., Slater, S., Roberts, I., Adams, L., Sculpher, M., Wade, A. and McCarthy, M. (1999)[65]	London, UK	RCT	Provision and installation of free smoke alarms, plus written fire safety information
5. McConnell, C.F., Dwyer, W.O. and Leeming, F.C. (1996)[66]	Memphis, USA	Non RCT	Fire safety education (including use of video, written and other material)

6.	Roberts, H., Curtis, K., Liabo, K., Rowland, D., DiGuiseppi, C. and Roberts, I. (2004)[67]	London, UK	RCT	Provision and installation of smoke alarms, plus written fire safety information
7.	Young, M., Camit, M. and Mihajlovic, M. (1999)[68]	New South Wales, Australia	Non RCT	Provision of discounted smoke alarms, plus written fire safety information and alarm demonstration

Before describing the NS work undertaken, we first consider the nature of the research evidence likely to be included in reviews of factors shaping the successful implementation of particular interventions.

5.2 A note on extracting data on implementation.

There are at least four issues that reviewers have to deal with when extracting data on implementation from reports of interventions. These are:

- Locating the data in the text
- Establishing the nature and type of implementation data
- Ascertaining their provenance and reliability
- Extracting the data in preparation for analysis

In relation to the first, it should be noted that reports of interventions tend not to contain a great deal of detail on process issues - implementation is rarely the focus of reports of interventions. This may be because interventions are often reported in journal papers, and there is not the space to describe the factors affecting implementation. If there are no additional documents available describing the implementation of an initiative some of these data may be found interspersed throughout the text, but are more likely to be located predominantly in the discussion section of papers, where authors usually attempt to provide an explanation for the effectiveness or otherwise of an intervention.

Once data have been identified the reviewers need to establish the nature and type of implementation data found in the text. Most data of this kind consist of simple narrative observations made by authors of the factors affecting the campaign or initiative. It is relatively rare for primary data (direct quotations from users, for instance) to be found to support these observations.

Ascertaining the provenance and reliability of data on implementation can be the most difficult and frustrating part of the process of extracting data on implementation. It is rare to find details of where these data (either in the form of narrative author observations or quotations) come from, or what they are based on. Are authors drawing on fieldworkers' observations? Or unreported focus group findings? Are these just 'hunches'? Where data are 'thicker' (perhaps where qualitative data have been presented) additional maybe unused and/or unreported data on implementation may be available.

Finally, how should those attempting a synthesis extract the data in preparation for analysis? Where authors have made simple observations about implementation, these can be taken whole from the text (and the presentation/reporting of this will depend on the number and complexity of such observations). Quotations or other qualitative data can also be taken directly from the text and the data entered into tables with appropriate headings (for example: author/paper; location of implementation data in the report; type and nature of data).

5.3 The narrative synthesis

The guidance is structured around four elements in the synthesis process:

- Developing a theory of how the intervention works, why and for whom
- Developing a preliminary synthesis
- Exploring relationships within and between studies
- Assessing the robustness of the synthesis

In the NS reported here no *prima facie* attempt was made to develop a theoretical basis for the work so this element of the guidance was not applied. The NS was carried out separately by two different members of the study team, each using the draft guidance to test its usefulness and clarity. One reviewer worked with all seven papers, and the other worked with only three papers.

The guidance presents a number of related tools and techniques that can be used to complete the various elements of the synthesis. To apply the guidance to the narrative synthesis of implementation evidence, each of the sections was read through in sequential order, and for each element the tools and/or techniques that appeared to be useful and relevant to the synthesis at hand were selected. The results of this exercise are shown in Table 10. The tools and techniques were either used in the worked examples (Yes), not used and probably not relevant (No), or not used but potentially relevant (Potentially yes). The reasons for selecting or rejecting a tool or technique are discussed in more detail within each section. Where tools or techniques proved to be less useful, this is also discussed.

Table 10. Tools and techniques for the implementation syntheses

**Tools and techniques**	**Useful for NS of implementation data?**
**Preliminary Synthesis**	
Textual descriptions	Yes
Tabulation	Yes
Groupings and clusterings	Yes
Constructing a common rubric	No
Thematic analysis	Yes
Content analysis	No
Vote counting	No
**Exploring relationships**	
Variability in outcomes	Potentially yes
Variability in study design	Potentially yes
Variability in study population	Potentially yes
Moderator variables and subgroup analyses	No
Idea webbing and subgroup analyses	Yes
Conceptual triangulation	Potentially yes
Translation	Yes
Case descriptions	Potentially yes
Visual representation	No
Investigator triangulation and methodological triangulation	Potentially yes
**Assessing robustness**	
Weight of evidence	Potentially yes
Best evidence synthesis	Potentially yes
Checking with authors	Potentially yes
Critical reflection	Yes

In the following sections, we illustrate the practical application of the various tools and techniques discussed in earlier chapters providing a worked example of a narrative synthesis of data on the

factors affecting the implementation of interventions aiming to increase the uptake of domestic smoke alarms. A flow chart summarising the synthesis process as a whole is presented in figure 8 below. Some of the worked examples have been limited by the relatively small size of the evidence base as several of the tools and techniques are only relevant when synthesising a large body of literature.

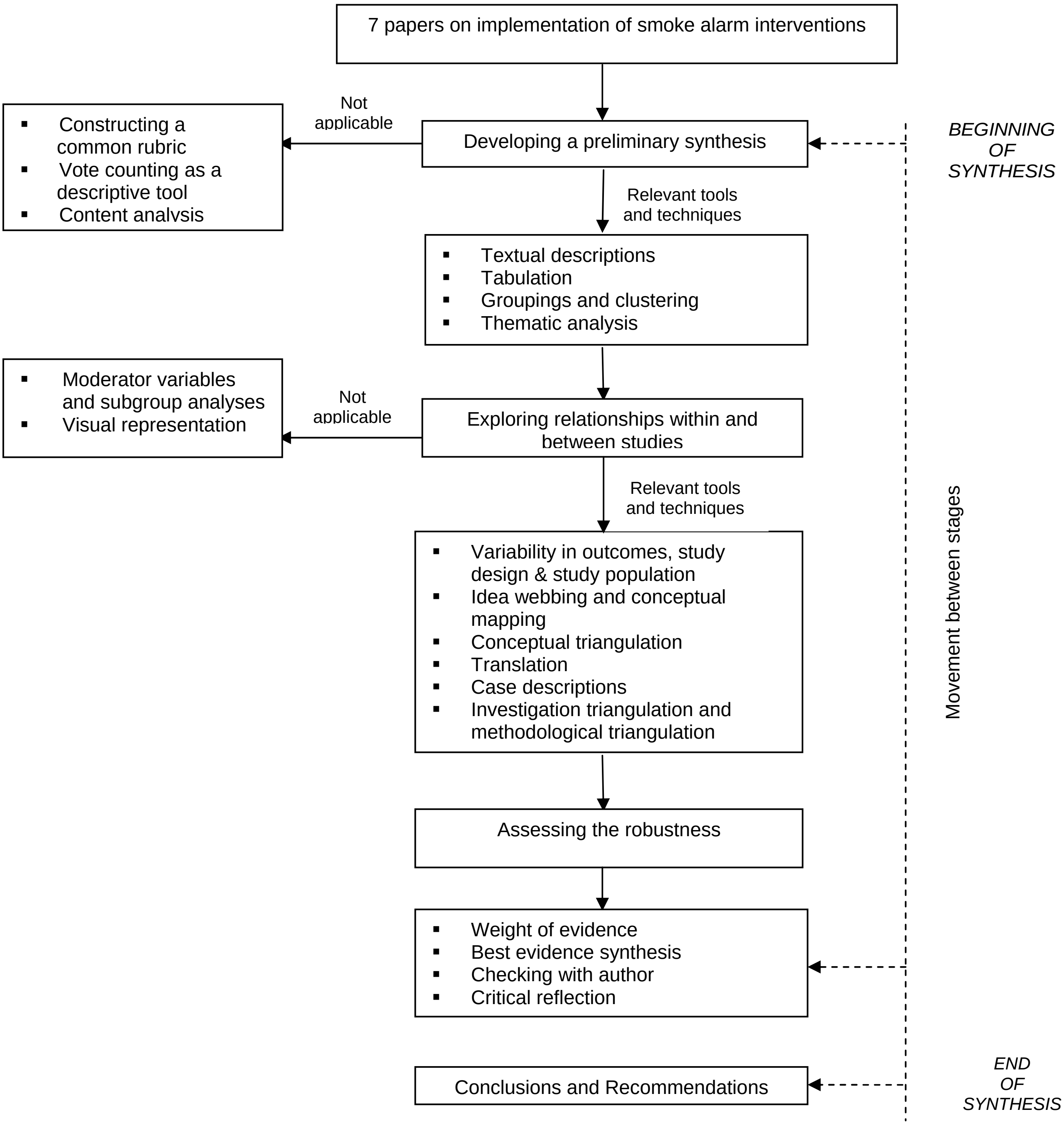

Figure 8: Synthesis process

Developing a preliminary synthesis

Textual description

This was used by both reviewers at an early stage, and was found useful as a way of summarising the papers and beginning to extract information in a systematic way. Textual descriptions offer the potential to include more details than, for example, tabulations.

Examples of textual description:

Example 1

> In McConnell et al,[66] the **target population** was new heads of households in public housing residences of the Memphis Housing Authority (MHA), USA and they were predominantly female Afro Americans living with children. The MHA policy is to ensure that a functioning smoke detector is in every unit when rented, but a spot check of 325 units in 1992 found that less than 8% had a working smoke detector. The 35 minute **intervention** (delivered during mandatory orientation sessions for new MHA heads of household) consisted of the following components: a pre test; videotape accompanied by lecturettes delivered by one of 36 uniformed fire fighters, one MHA supervisor or one civilian educator; behavioural contract, post test, and fire-safety reminder card. The **outcomes** were fire incidence data (after possibly 15 months, timescale not clear); residents' evaluations of the programme; changes in their fire safety knowledge; and their commitments to fire safety behaviours. The method of **evaluation** was an uncontrolled comparison between trained and untrained residents, using contemporary and historical comparison groups. The evaluation **data** were all quantitative. The **results** showed a lower incidence of fires in trained residents compared with untrained residents (1 fire for every 4312 renter months in trained residents compared with 1 fire for every 780 renter months in untrained residents; a relative risk of 5.5). Comparing trained residents with untrained residents over the 9 year baseline period gave a relative risk of 4.8. Comparisons between newer and older residents from the MHA records suggested that newer residents were more likely to experience fires, thus countering the suggestion that the results can be explained by the fact that the trained residents were also new residents. No data were provided on the proportion of working smoke detectors post intervention.

Example 2

> Young et al (1999),[68] Camit (2002)[64] and Camit (1998)[63] report on the effectiveness and implementation of a smoke alarm promotion campaign in NSW Australia oriented to the needs of Arabic, Chinese and Vietnamese communities. Qualitative data were collected in focus groups and interviews. Survey data were also collected. Their main observations in relation to implementation are that among the target community there was a lack of awareness of the need for smoke alarms. Living in rented property where the landlord was thought to be unsympathetic to the need for a smoke alarm also created barriers to the installation of smoke alarms.

Tabulation

Both reviewers felt that tabulation and textual descriptions were very similar, possibly using the same headings but laid out differently. In a table, however, it was easier to compare data across different studies.

Table 11. Example of tabulation

Author & year	Location & setting	Target population	Method	Main findings
Roberts et al (2004)[67]	London, UK<br>Urban	All residents on estate (n=40000 households)	Focus groups and interviews	Problems with smoke alarms (sensitivity, false alarming) identified as major barriers to implementation
Camit (2002)[64]<br>Young et al (1999[68])<br>Camit (1998)[63]	NSW, Australia<br>Mixed	Chinese, Vietnamese, Arabic-speaking (not given)	Focus groups	Implementation successful using multi-faceted, language appropriate approach
Campbell De Long Resources (2003)[62]	Oregon, USA<br>Mixed	All residents, but focus on Latino-speaking<br>(sample population varied according to element of intervention)	Interviews	Successful implementation heavily dependent on landlords' attitudes

Groupings and clusters

This technique is more useful when there are larger numbers of papers. The type of groups identified is likely to depend on the reviewers categorisations, but may also depend on the document type (is it describing a trial or a campaign, for example). The result is similar to tabulation, except that the relationships (or differences) between groups of studies can be made more explicit when they are clustered in this way.

Table 12. Example of grouping

**Grouping according to:**		
Location	***Focus of report***	***Population***
UK (DiGuiseppi et al 1999; Roberts et al 2004)	Broad, general factors affecting programme (DiGuiseppi et al 1999; Camit 1998; Camit 2002)	Ethnically mixed (Camit, 1998; Camit, 2002; Young et al, 1999)
USA: (Campbell De Long Inc 2003; McConnell et al, 1996)	Individual-factors affecting programme (Young et al, 1999; McConnell et al 1996; Roberts et al, 2004; Campbell de Long Inc 2003)	Ethnically mixed and low income (Campbell de Long Inc, 2003; McConnell et al 1996; DiGuiseppi et al 1999; Roberts et al 2004
Australia: Camit 1998; Camit 2002; Young et al 1999)		

Transforming the data: constructing a common rubric

This technique was not found useful by the reviewers, as there was no unit of measurement that could form the basis of a common rubric. The data from the intervention studies in this review (installation rates of purchased smoke alarms, fire incidence data, and percentages of working smoke alarms) did not lend themselves to transformation in this way, though it may be feasible for other reviews where the outcomes are more homogeneous.

Translating data: thematic analysis

Different reviewers, and the same reviewer at different times, identified different themes. These themes were identified on an inductive basis by reading and re-reading the papers. They fell into two categories: those aspects of the interventions that seemed to act as barriers or facilitators, in the view of the author or the reviewer; and themes identified by the authors from their qualitative data.

Example of thematic analysis

Example 1

- The smoke alarm
- The individual and
- The community

Table 13: Example 2

	McConnell[66]	Young[68]	Roberts[67]
**Facilitators**	**Aspects of intervention**:<br>Mandatory orientation sessions<br>Formal written commitment<br>Involvement of fire service<br>Tailored intervention; involvement of residents in its design<br>Reminder card<br>Penalties for arson and disabling smoke detectors explained<br>Testing of knowledge<br>Smoke detectors already installed<br>Commitment of landlord (MHA)		Early warning in the event of fire and increased sense of security<br>Feeling at high risk for fire<br>Fitting of the smoke alarms by installers
**Barriers**		**Lack of awareness**<br>Lack of awareness of the need for smoke alarms<br>Unsympathetic landlords<br>Tendency to overestimate installation costs<br>Underestimation of fire risk<br>Lack of awareness of danger of smoke<br>Perception that fire is a hazard only for wooden homes<br>Not relating potential benefits to their own circumstances<br>**Rented accommodation**<br>Landlords holding residents liable for installation damage<br>Frequent moving<br>Having to leave alarms behind when moving<br>Landlords withholding permission<br>Difficulty of applying to estate agents<br>**Installation**<br>Lack of time	Concerns about strangers entering their home<br>Suspicion of an intervention provided free<br>Feeling oneself "too old to be worth spending money on"<br>Alarms as a source of stress<br>Problems with maintenance<br>Alarm sensitivity<br>Alarms as nuisance<br>Alarms as a threat to immediate well-being

Table14: Example 3

- Barriers/levers to the acquisition of smoke alarms
- Barriers/levers to the installation of smoke alarms
- Barriers/levers to the continued use of smoke alarms

**1) Barriers/levers to acquisition of smoke alarms**		
General		
	Barriers	*Levers*
	Problems accessing communities/gatekeepers	Gaining trust of key community ‘players’ and leaders
	Suspicion of ‘authority’ or local government	Emphasising separation from distrusted authority/alliance with trusted partners
Specific to smoke alarm campaigns		
	Lack of awareness of benefits of smoke alarms	Running well-coordinated, culturally appropriate awareness campaign
	Perceived cost of smoke alarms	Giveaway or availability of reduced price alarms
	Perception that household is not at risk of fire (due to type of house or characteristics of household members)	Awareness campaign
**2) Barriers/levers to installation of smoke alarms**		
General		
	Barriers	*Levers*
	Anxiety about damage to property	Landlord approval/permission for installation, or landlord example of installation
Specific to smoke alarm campaigns		
	Inability/unwillingness to install alarm	Installation of alarm by project worker
**3) Barriers/levers to continued use of smoke alarms**		
	Barriers	*Levers*
	False alarms	Education about triggers for false alarms/re-installation of alarm
	Problems with maintenance	Project workers offer to maintain alarms/education about maintenance

The differences over time or between reviewers do not suggest that the synthesis is flawed but rather draws attention to different ways of interpreting the same data. Both reviewers identified a typology including facilitators and barriers as did some of the study authors. Whether the data are seen ecologically or in stages, the idea of barriers and facilitators are common to both. In a final synthesis, specific factors that act as barriers/levers, the notion of stages (temporality) and the organisation of these factors within domains at different levels (ecological perspective) could be brought together.

Translating the data: Content analysis

This technique was not found useful by either reviewer as the data did not lend themselves to conversion into frequencies.

Vote counting as a descriptive tool

Similarly, this technique was not found useful by either reviewer. The description of vote counting in the guidance focuses primarily on effectiveness reviews. Although in theory it could be used to 'count' up facilitators and barriers it would probably only be appropriate with a larger number of studies included in the review and reviewers would need to be aware of the disadvantages of using this technique inappropriately as discussed earlier in this guidance.

Exploring relationships within and between studies

Exploring the influence of heterogeneity[69]

Only one reviewer attempted to use this technique, which focused attention on the characteristics of the different studies and their potential relationships to the findings. The other reviewer did not find this technique useful.

Example of variability in outcomes

> The quantitative outcomes of the three studies relate to three different stages of the trajectory of a domestic fire: installation of a smoke alarm; continued working of a smoke alarm; incidence of domestic fires. In the Young paper, installation rates varied from 65% to 35% after 10-15 days (with some suggestion that these rates rose after 2 months).[68] Given that the installation rate in the Roberts paper[67] was 100% for all respondents, but the proportion of working alarms at 15 months was only 51%, this suggests that the proportion of working alarms in the Young study would have been even lower. It seems unlikely that these proportions of working alarms would have produced the relative risk results of the McConnell paper (assuming of course that their relative risk figures are robust). Given that the McConnell paper[66] did not measure the proportion of working alarms in their trained and untrained groups, this is speculative; it may be the case that in such environments, low proportions of working alarms can deliver measurable improvements in the incidence of domestic fires. This is supported by McConnell's data on pledges made about fire safety behaviours: only 24% of the pledges made by trained residents were commitments to keep smoke detectors in working order. However one of the five points on the reminder card in the McConnell paper was "Keep your smoke detector working and check it often". I would have to look beyond these papers to try and assess the comparability of the outcomes of these papers. My own working assumption is that the intervention evaluated in the McConnell study was more successful, perhaps because it showed an improvement in the target (fire incidence), as well as proxy (residents' fire safety knowledge), outcomes.

Example of variability in interventions

> The interventions were different: an educational intervention for residents with already installed smoke alarms (McConnell); the sale of smoke alarms (Young); and the free installation of smoke alarms (Roberts). However one aspect of the interventions is potentially explanatory. The intervention in the McConnell study was drawn up following focus groups and individual residents in which they were asked about the best approaches to be used; the Roberts paper concluded that "It may well be that a more appropriate design would have started with qualitative work, improving take up and maintenance instructions". The Young study was not drawn up in consultation with the local community. Thus the involvement of the local population and the development of a

tailored intervention may well have influenced the success of the McConnell study. The 5.5 minute videotape in the McConnell study “depicted actual MHA residents, apartments and structures” and was described by MHA residents who previewed it as “extremely powerful and likely to make a lasting impression”. The McConnell intervention was underpinned by a model of community psychology, the goal of which is “to optimise the well being of communities and individuals with innovative and alternative interventions designed in collaboration with affected community members and with other related disciplines inside and outside of psychology”. Other components of the McConnell intervention seem likely to have influenced its success: the mandatory orientation sessions which meant that all new residents received the intervention whether they wanted to or not; the formal written commitments obtained at the end of the educational session; the involvement of the fire service who delivered some of the interventions themselves; and the reminder card.

Moderator variables and subgroup analyses
These were not found useful by either reviewer, as they are primarily helpful in effectiveness syntheses.

Idea webbing and conceptual mapping
We found it impossible to distinguish between these two techniques and so have combined them. The use of figures/diagrams was found to be helpful for exploring issues on implementation. One reviewer does not usually use these kinds of visual methods and only did so to test the guidance; but then felt that it had helped her to think about the relationships between the themes rather more than she would have done otherwise.

Much depends on how complex and detailed the figures are, and how easy they are to ‘read’. There may be too much information in them (lots of bubbles or balloons making it hard to see what the message is). The structure of the figures tended to replicate themes identified by the thematic analysis. In a sense these figures represented an early product of the synthesis. It was clear that at this stage they were specific to smoke alarms and could not necessarily be applied to other types of intervention.

Figure 9. Examples of idea webbing

a)

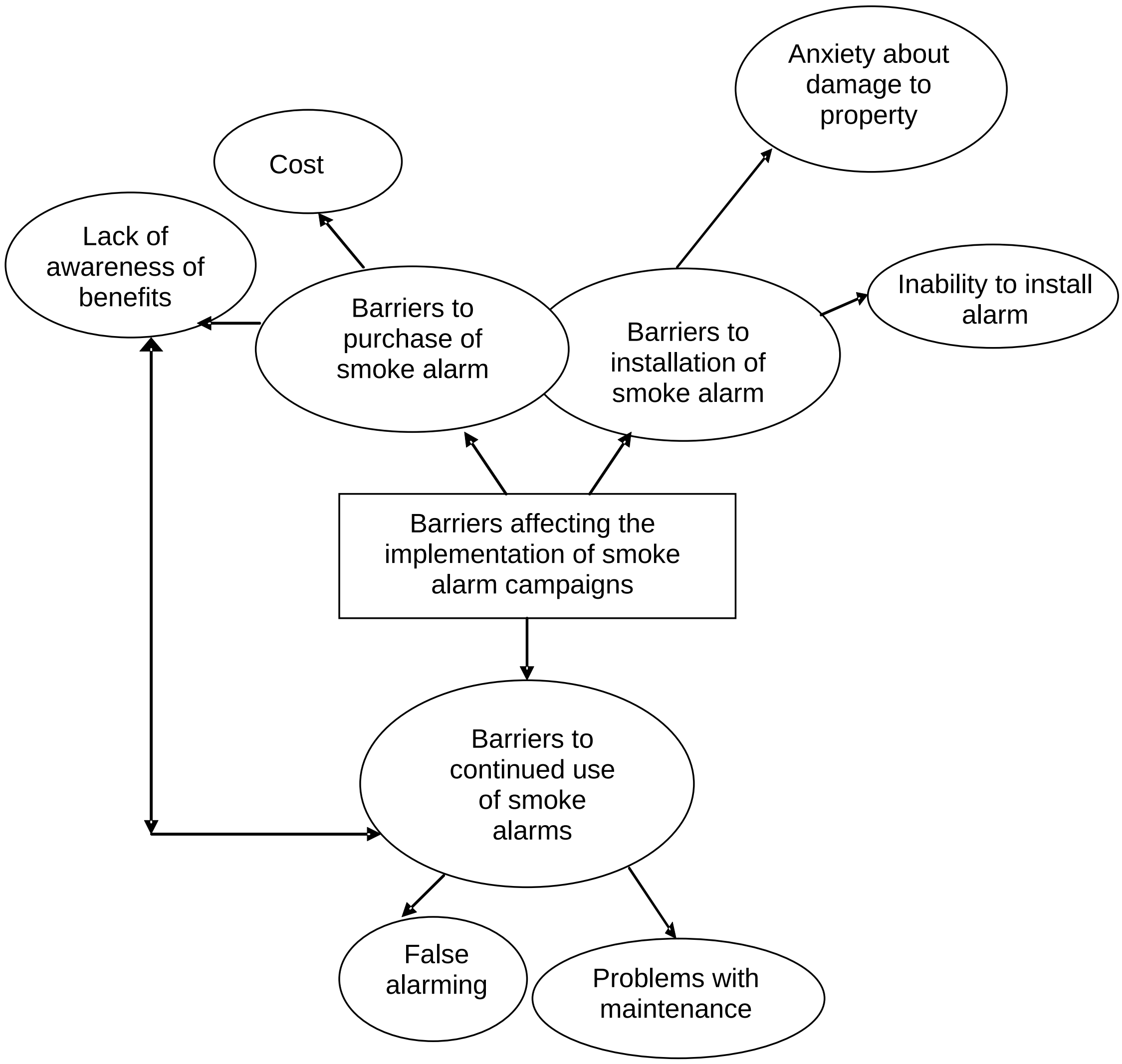

b)

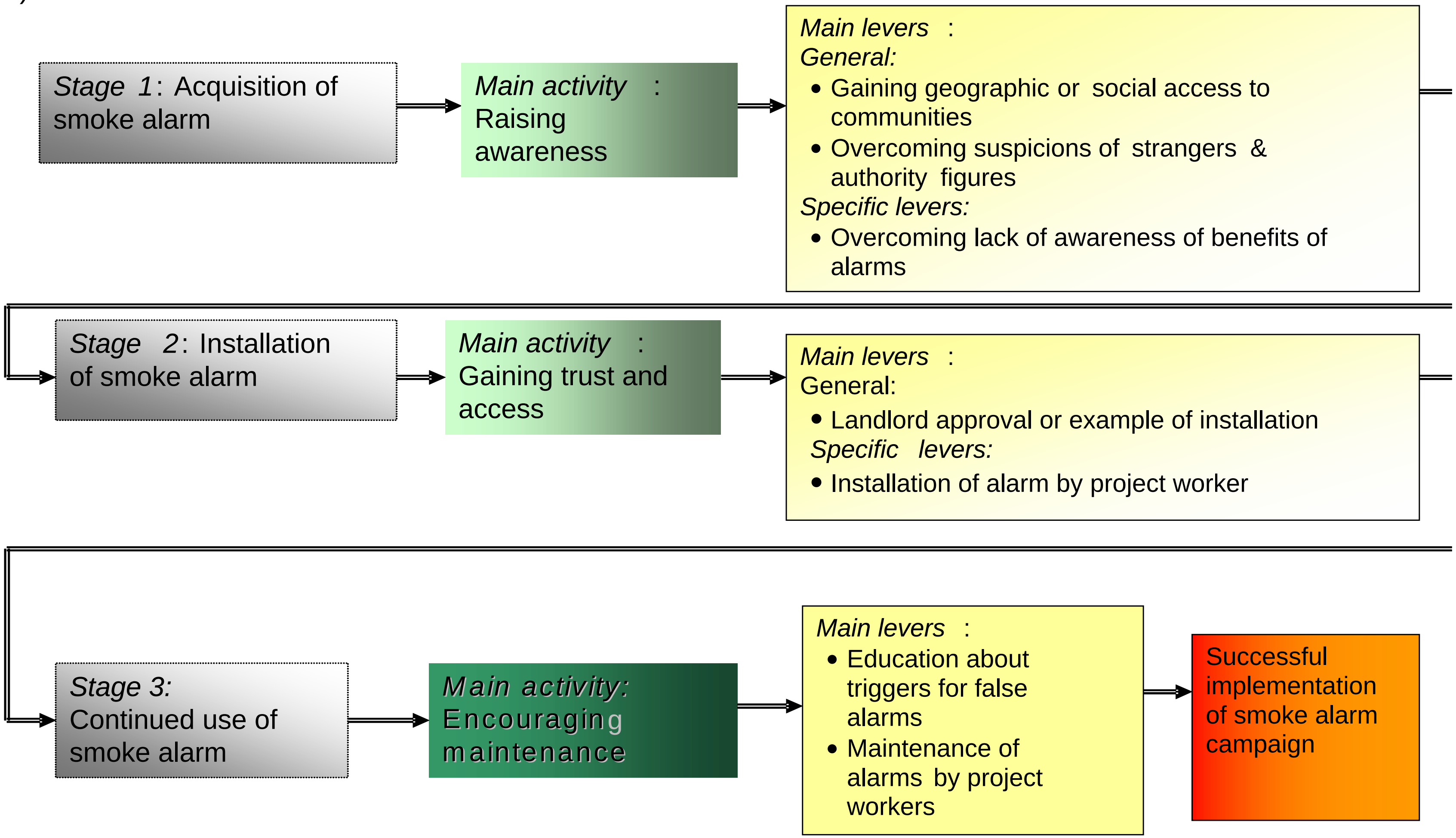
Stage 1: Acquisition of smoke alarm
Main activity: Raising awareness
Main levers:
General:
• Gaining geographic or social access to communities
• Overcoming suspicions of strangers & authority figures
Specific levers:
• Overcoming lack of awareness of benefits of alarms
Stage 2: Installation of smoke alarm
Main activity: Gaining trust and access
Main levers:
General:
• Landlord approval or example of installation
Specific levers:
• Installation of alarm by project worker
Stage 3: Continued use of smoke alarm
Main activity: Encouraging maintenance
Main levers:
• Education about triggers for false alarms
• Maintenance of alarms by project workers
Successful implementation of smoke alarm campaign

c)

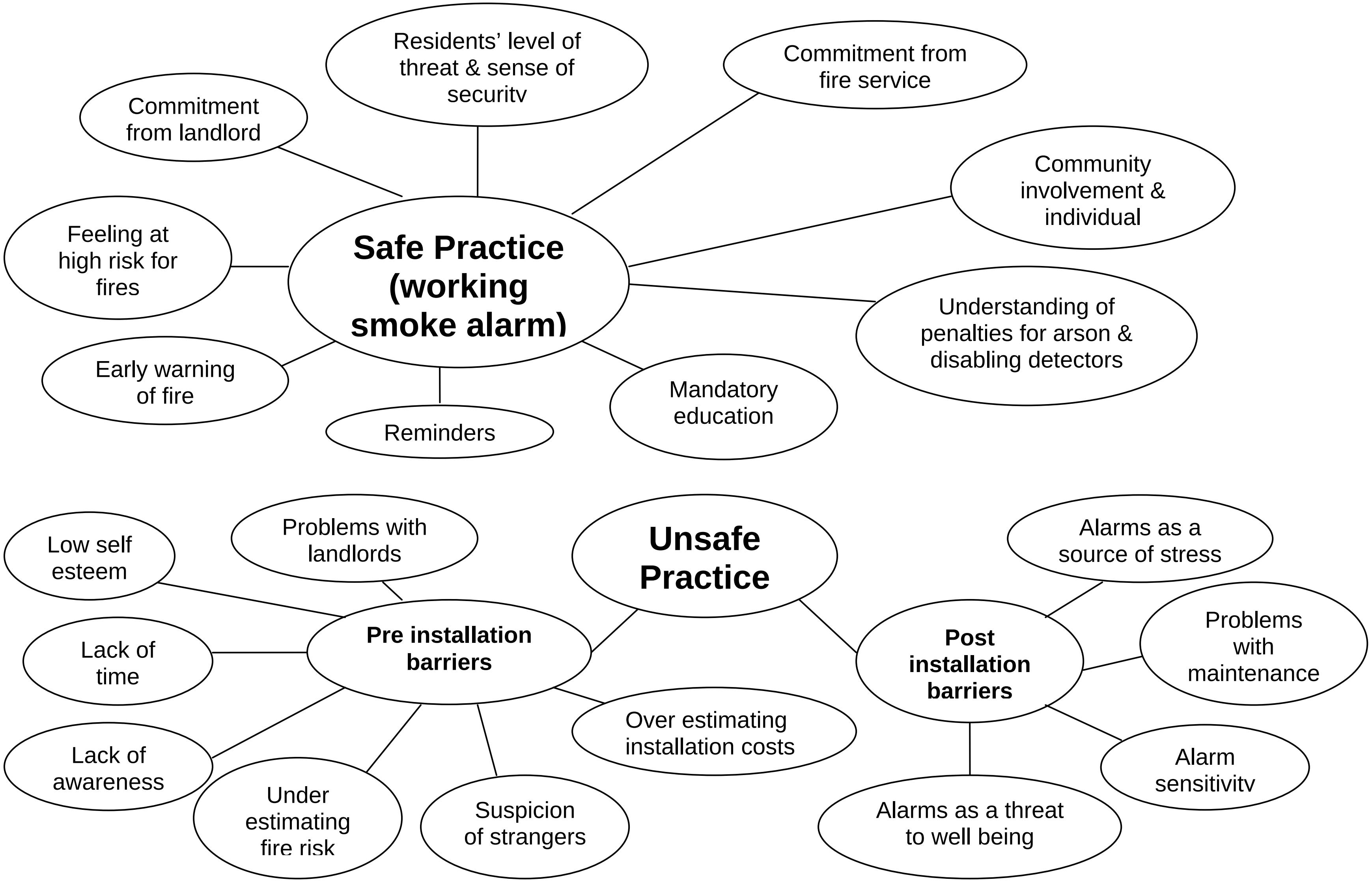

Residents' level of threat & sense of securitv
Commitment from fire service
Commitment from landlord
Community involvement & individual
Feeling at high risk for fires
Safe Practice (working smoke alarm)
Understanding of penalties for arson & disabling detectors
Early warning of fire
Mandatory education
Reminders
Unsafe Practice
Low self esteem
Problems with landlords
Alarms as a source of stress
Lack of time
Pre installation barriers
Post installation barriers
Problems with maintenance
Over estimating installation costs
Lack of awareness
Under estimating fire risk
Suspicion of strangers
Alarms as a threat to well being
Alarm sensitivitv

Conceptual triangulation
Neither reviewer found this technique useful for this exercise but felt that it would be useful with a larger number of studies.

Translation as an approach to exploring relationships
One reviewer with no previous experience of meta-ethnography attempted unsuccessfully to use it. The other reviewer, who does have previous experience of meta-ethnography, felt that translation was the most useful technique at this stage.

Example of reciprocal translation

> There are three concepts that seem to offer themselves for translation across studies. The **first** is the one of landlord commitment/lack of commitment. The difficulties with landlords discussed in the Young paper seem to be the exact opposite of the commitment demonstrated by the MHA in the McConnell paper, but not explicitly commented on by the authors. The **second** concept is risk perception: feeling oneself at high risk (Roberts), or underestimating the risk of fire (Young). The McConnell intervention presumably increased residents' estimates of their own risk but no information is provided about this. The **third** and less robust concept is residents' level of trust (or something like that). In the McConnell paper, residents were involved in the development of the intervention and presumably this generated a certain amount of trust (even though the education was mandatory). In the Roberts paper, the fact that some residents were uncomfortable with strangers coming into their homes to fit smoke alarms, and suspicious of anything offered for free, suggests a lack of trust. These three concepts may just represent the potential for translation between the three studies. However they may also start to characterise the elements necessary for a successful intervention: landlord commitment, risk perception, and residents' level of trust.

This kind of translation could eventually lead to an explanation or theory, which might form one output of the synthesis and which might itself inform future interventions.

Qualitative case descriptions
Neither reviewer found this useful, partly because there was little practical advice about how to use it and partly because it appeared similar to the earlier technique of textual description. It could be useful in building up some kind of composite picture of successful interventions and providing the kind of detail that could be useful for those wanting to design interventions themselves.

Visual representation of relationships between study characteristics and results
These techniques seemed to be appropriate for statistical data rather than the kinds of data in the implementation studies.

Investigator triangulation and methodological triangulation
Neither reviewer used these techniques due to lack of sufficient data in the primary studies, but both felt that they could be useful if there were more data available.

Assessing the robustness of the synthesis
Comparison with earlier review
The results achieved using the guidance were compared to the earlier review referred to at the beginning of this chapter.[61] This involved a simple thematic analysis of the data, with no attempt at synthesis using other tools or techniques. The conclusions identified features present in papers containing a 'thicker' description of implementation processes. The authors concluded that where interventions had been successfully implemented, the programmes were likely to have the following features:

- A relatively detailed description of the intervention, its strengths and weaknesses and its suitability for the targeted population:
    - The type of intervention used, and its appropriateness for the target population, affects the outcome of trials and other initiatives.
- Some consideration of the context within which the trials take place.

    - This is frequently limited to a discussion of the problems encountered by those implementing the programme rather than those receiving it and rarely substantiated by reference to data collected during the trial (or, indeed, any other data). From a methodological perspective, this constrains the use of these insights.

- Some recognition of the discrepancy between the design and orientation of an intervention and its implementation in an everyday setting.
- Some exploration of the reasons for anomalous results and findings.
- Some description of the factors that affect implementation.
    - Includes: the importance of understanding the people and the community receiving the intervention; the need to consider the role of community leaders and other key local figures to programme success; recognition that the characteristics of the community affect programme success.

These are useful though rather general insights into factors affecting implementation. In fact, this is less a synthesis than a list of insights about implementation drawn from the papers/reports.

Weight of evidence
This was not used as no assessment was made of the quality of individual studies. This technique could be useful if there were more primary studies available for review.

Best evidence synthesis
Similarly, this technique was not used for the worked example but could be useful if there were more primary studies.

Checking with the authors of primary studies
This is potentially useful if time allows, but depends on the accessibility and generosity of authors in providing further information. (Those we identified in the original study were helpful.) Given the general paucity of information relating to implementation of interventions in the demonstration studies, it would have been very useful to gain more details about the interventions and the contexts in which they were implemented.

Critical reflection
The synthesis of the implementation studies was based on a small number of studies, better suited for an exercise like this one where we are considering the processes of narrative synthesis than for providing a definitive synthesis product or "answer" (though it should be remembered that at present, many, perhaps most reviews are based on similarly small numbers of studies with similarly scant data on implementation).

By comparing the work of two reviewers we can examine inter-researcher differences. They used the different tools and techniques in parallel ways, not necessarily identifying precisely the same themes or concepts. Indeed, the reviewer working with all seven studies carried out two thematic analyses at different times, and identified different themes in her later reflections. The product of the synthesis will reflect the experience of the reviewers as well as their familiarity with tools and techniques. The reviewer with previous experience of meta-ethnography for example, found it easy to use the technique of translation to compare the concepts of different studies. No attempt was made in the demonstration project to achieve consensus between the reviewers, as we were interested in documenting their separate attempts and describing differences.

In comparison with the earlier exploratory review, the guidance enabled a more systematic overview of the different papers and a more nuanced appreciation of the evidence. As a justification for narrative synthesis is based in part on its claim to address the potential for bias, this demonstration review has shown that the use of specific tools and techniques can provide transparency of process. A more robust product is likely to be achieved if at least two reviewers work independently and then compare their findings to produce a mutually agreed (or a transparently divergent) final version.

CHAPTER 6: THE NEXT STEPS

Narrative approaches to synthesis are widespread in systematic reviews yet as we have noted these approaches do not rest on an authoritative body of knowledge. The guidance presented here has been developed on the basis of an extensive review of methodological literature and it has been applied to two contrasting bodies of evidence – one focusing on the effects of interventions to promote the use of domestic smoke alarms and the other focusing on evidence to inform the implementation of such interventions. In undertaking these demonstration syntheses detailed notes were kept of all major decisions taken and the reasoning behind them. This approach of prospectively documenting the synthesis process was a helpful aid to transparency and recall. We would recommend this to all reviewers adopting a narrative approach.

We do not claim to have produced the definitive guide to narrative synthesis – there is much work still to be done to develop and refine this approach to evidence synthesis. However, we do believe that the guidance offers both a general framework and specific tools and techniques that can help to increase the transparency and trustworthiness of systematic reviews involving narrative synthesis. We would also stress that whilst the guidance describes a range of tools and techniques that if used appropriately will improve the process of narrative synthesis these will not remove the need for reviewers to combine sound methodology with creative interpretative work.

We hope that people will find the guidance useful and that they will let us have feedback so that we can revise the guidance in light of this. The guidance is to be made available on the project website (address to be added) and comments can be sent by email to j.popay@lancaster.ac.uk

REFERENCES

1. NHS Centre for Reviews and Dissemination. *Undertaking systematic reviews of research on effectiveness: CRD's guidance for carrying out or commissioning reviews. CRD report 4*. 2nd ed. York: NHS Centre for Reviews and Dissemination, 2001.
2. Dixon-Woods M, Agarwal S, Young B, Jones D, Sutton A. *Integrative approaches to qualitative and quantitative evidence*. London: NHS Health Development Agency, 2004. Available from: http://www.hda.nhs.uk/documents/integrative_approaches.pdf
3. Human Resources at Massachusetts Institute of Technology. *Performance review forms [monograph online]*. Human Resources at Massachusetts Institute of Technology; [cited Jan 2006]. Available from: http://web.mit.edu/hr/performance/forms.html.
4. Eisen GM, Weinberg DS. Narrative review: screening for colorectal cancer in patients with a first-degree relative with colonic neoplasia. *Ann Intern Med* 2005;143:190-98.
5. Higgins JPT, Green S. *Cochrane handbook for systematic reviews of interventions 4.2.5 [updated May 2005]*. Available from: http://www.cochrane.org/resources/handbook/index.htm (accessed 31st May 2005)
6. EPPI Centre. *EPPI-Centre Review Group Manual: working document version 1.1 [monograph online]*. London: EPPI Centre, 2001. Available from: http://eppi.ioe.ac.uk/EPPIWebContent/downloads/RG_manual_version_1_1.pdf
7. Weiss CH. *Evaluation: methods for studying programs and policies*. New Jersey: Prentice-Hall, 1998.
8. Shadish W. Meta-analysis and the exploration of causal mediating processes: a primer of examples, methods, and issues. *Psychological Methods* 1996;1:47-65.
9. Pawson R. *Does Megan's Law work? A theory-driven systematic review [monograph online]*. London: ESRC Centre for Evidence Based Policy and Practice, 2002. Available from: http://www.evidencenetwork.org/Documents/wp8.pdf
10. Miller N, Pollock VE. Meta-analytic synthesis for theory development. In: Cooper H, Hedges LV, editors. *The handbook of research synthesis*. New York: Russell Sage Foundation; 1994. p. 457-83.
11. Powell S, Tod J. A systematic review of how theories explain learning behaviour in school contexts. In: *Research Evidence in Education Library*. London: EPPI-Centre, Social Science Research Unit, Institute of Education; 2004. Available from: http://eppi.ioe.ac.uk/EPPIWeb/home.aspx?&page=/reel/reviews.htm
12. Pawson R, Greenhalgh T, Harvey G, Walshe K. *Realist synthesis: an introduction*. RMP Methods Paper 2/2004. Manchester: ESRC Research Methods Programme; 2004. Available from: http://www.ccsr.ac.uk/methods/publications/documents/RMPmethods2.pdf
13. Petticrew M, Roberts H. *Systematic reviews in the social sciences: a practical guide*. Oxford: Blackwell, 2006.
14. Light RJ, Pillemer DB. *Summing up: the science of reviewing research*. Cambridge, MA, USA: Harvard University Press, 1984.
15. Cook TD, Leviton LC. Reviewing the literature: a comparison of traditional methods versus meta-analysis. *J Pers* 1980;48:449-72.
16. Grimshaw J, Freemantle N, Wallace S, Russell IT, Hurwitz B, Watt I, et al. Developing and implementing clinical guidelines. *Qual Health Care* 1995;4:55-64.
17. Rychetnik L, Frommer M, Hawe P, Shiell A. Criteria for evaluating evidence on public health interventions. *J Epidemiol Community Health* 2002;56.
18. Hawe P, Shiell A, Riley T. Complex interventions: how "out of control" can a randomised controlled trial be? *BMJ* 2004;2004:1561-63.
19. Campbell M, Fitzpatrick R, Haines A, Kinmonth A, Sandercock P, Spiegelhalter D, et al. Framework for design and evaluation of complex interventions to improve health. *BMJ* 2000;321:694-6.
20. Jackson N, Cochrane Health Promotion and Public Health Field, Victorian Health Promotion Foundation. *Handbook: systematic reviews of health promotion and public health interventions*. Victoria, Australia: Deakin University, 2004.
21. Roen K, Arai L, Roberts H, Popay J. Extending systematic reviews to include evidence on implementation: methodological work on a review of community-based initiatives to prevent injuries [in press]. *Soc Sci Med* 2006.
22. Evans D. Systematic reviews of interpretive research: interpretive data synthesis of processed data. *Aust J Adv Nurs* 2002;20:22-6.

23. Long TJ, Convey JJ, Chwalek AR. *Completing dissertations in the behavioral sciences and education: a systematic guide for graduate students*. San Francisco: Jossey-Bass, 1985.
24. Burls A, Cummins C, Fry-Smith A, Gold L, Hyde C, Jordan R, et al. *West Midlands Development and Evaluation Service handbook [monograph online]*. Birmingham: West Midlands Development and Evaluation Service, 2000. Available from: http://www.publichealth.bham.ac.uk/wmhtac/pdf/wmhandbook.pdf
25. Mays N, Roberts E, Popay J. Synthesising research evidence. In: Fulop N, Allen P, Clarke A, Black N, editors. *Studying the organisation and delivery of health services: research methods*. London: Routledge; 2001. p. 188-220.
26. Mulrow C, Cook D. *Systematic reviews: synthesis of best evidence for health care decisions*. Philadelphia: American College of Physicians, 1998.
27. Hedges LV, Olkin I. *Statistical methods for meta-analysis*. London: Academic Press, 1985.
28. Cwikel J, Behar L, Rabson-Hare J. A comparison of a vote-count and meta-analysis review of intervention research with adult cancer patients. *Res Soc Work Pract* 2000;10:139-58.
29. Clinkenbeard PR. Beyond summary: constructing a review of the literature. In: Buchanan NK, Feldhusen JF, editors. *Conducting research and evaluation in gifted education: a handbook of methods and applications*. New York, NY: Teachers College Press; 1991. p. 33-50.
30. Curlette WL, Cannella KS. Going beyond the narrative summarization of research findings: the meta-analysis approach. *Res Nurs Health* 1985;8:293-301.
31. Suri H. A critique of contemporary methods of research synthesis. *Post-Script* 2000;1:49-55.
32. Hunt M. Is it true that "throwing money at schools" does no good? In: Hunt M, editor. *How science takes stock: the story of meta-analysis*. New York: Russell Sage Foundation; 1997. p. 54-57.
33. Hanushek EA. Throwing money at schools. *Journal of Policy Analysis and Management* 1981;1:19-41.
34. Hedges LV, Laine RD, Greenwald R. Does money matter? A meta-analysis of studies of the effects of differential school inputs on student outcomes. *Educational Researcher* 1994;23:5-14.
35. Noblit GW, Hare RD. *Meta-ethnography: synthesizing qualitative studies*. London: Sage, 1988.
36. Mays N, Pope C, Popay J. Systematically reviewing qualitative and quantitative evidence to inform management and policy-making in the health field. *J Health Serv Res Policy* 2005;10 Suppl 1:6-20.
37. Stemler S. An overview of content analysis. *Practical Assessment, Research and Evaluation [serial online]* 2001 [cited 2006 Jan 5];7. Available from: http://PAREonline.net/getvn.asp?v=7&n=17
38. Cooper H, Hedges LV, editors. *The handbook of research synthesis*. New York: Russell Sage Foundation, 1994.
39. Cooper HM. *Synthesizing research: a guide for literature reviews*. 3rd ed. London: Sage, 1998.
40. Mulrow C, Langhorne P, Grimshaw J. Integrating heterogeneous pieces of evidence in systematic reviews. In: Mulrow C, Cook D, editors. *Systematic reviews: synthesis of best evidence for health care decisions*. Philadelphia: American College of Physicians; 1998. p. 103-12.
41. Foster RL. Addressing epistemologic and practical issues in multimethod research: a procedure for conceptual triangulation. *ANS Adv Nurs Sci* 1997;20:1-12.
42. Begley CM. Using triangulation in nursing research. *J Adv Nurs* 1996;24:122-8.
43. Barbour RS, Barbour M. Evaluating and synthesizing qualitative research: the need to develop a distinctive approach. *J Eval Clin Pract* 2003;9:179-86.
44. Maggs-Rapport F. Combining methodological approaches in research: ethnography and interpretive phenomenology. *J Adv Nurs* 2000;31:219-25.
45. Gough D, Oakley Elbourne D. Methodologies for the systematic synthesis of experimental and non-experimental designs [poster]. In: *1st Campbell Collaboration Methods Group Conference*; 2002 Sep 17-19; Baltimore, Maryland, USA. 2002.
46. Slavin RE. Best-evidence synthesis: an alternative to meta-analytic and traditional reviews. In: Shadish WRJ, Reichardt CS, editors. *Evaluation studies: review annual*. Thousand Oaks, CA: Sage Publications, Inc.; 1987. p. 667-73.
47. Slavin RE. Best evidence synthesis: an intelligent alternative to meta-analysis. *J Clin Epidemiol* 1995;48:9-18.
48. Centers for Disease Control and Prevention. *The guide to community preventive services: what works to promote health? Task Force on Community Preventive Services [monograph online]*. Atlanta, GA: Centers for Disease Control and Prevention, 2005. Available from: http://www.thecommunityguide.org/

49. Busse R, Orvain J, Velasco M, Perleth M, Drummond M, Gurtner F, et al. Best practice in undertaking and reporting health technology assessments. Working group 4 report. *Int J Technol Assess Health Care* 2002;18:361-422.
50. Britten N, Campbell R, Pope C, Donovan J, Morgan M, Pill R. Using meta ethnography to synthesise qualitative research: a worked example. *J Health Serv Res Policy* 2002;7:209-15.
51. DiGuiseppi C, Higgins JPT. *Interventions for promoting smoke alarm ownership and function*. In: The Cochrane Database of Systematic Reviews: Reviews 2001 Issue 2 John Wiley & Sons, Ltd Chichester, UK DOI: 10.1002/14651858.
52. Arai L, Roen K, Roberts H, Popay J. It might work in Oklahoma but will it work in Oakhampton? Context and implementation in the effectiveness literature on domestic smoke detectors. *Inj Prev* 2005;11:148-51.
53. Jadad AR, Moore RA, Carroll D, Jenkinson C, Reynolds DJ, Gavaghan DJ, et al. Assessing the quality of reports of randomized clinical trials: is blinding necessary? *Control Clin Trials* 1996;17:1-12.
54. Kelly B, Sein C, McCarthy PL. Safety education in a pediatric primary care setting. *Pediatrics* 1987;79:818-24.
55. Kendrick D, Marsh P, Fielding K, Miller P. Preventing injuries in children: cluster randomised controlled trial in primary care. *BMJ* 1999;318:980-3.
56. Ploeg J, Black MF, Hutchison BG, Walter SD, Scott EAF, Chambers IW. Personal, home and community safety promotion with community-dwelling elderly persons: response to a public health nurse intervention. *Can J Public Health* 1994;85:188-91.
57. Williams GE. *An analysis of prenatal education classes: an early start to injury prevention [dissertation]*. Kansas City, KS: University of Kansas, 1988.
58. Clamp M, Kendrick D. A randomised controlled trial of general practitioner safety advice for families with children under 5 years. *BMJ* 1998;316:1576-9.
59. Jenkins HM, Blank V, Miller K, Turner J, Stanwick RS. A randomized single-blind evaluation of a discharge teaching book for pediatric patients with burns. *J Burn Care Rehabil* 1996;17:49-61.
60. King WJ, Klassen TP, LeBlanc J, Bernard-Bonnin AC, Robitaille Y, Pham B, et al. The effectiveness of a home visit to prevent childhood injury. *Pediatrics* 2001;108:382-8.
61. Popay J, Arai L, Roberts H, Roen K. *Exploring methodological and practical issues in the systematic review of factors affecting the implementation of child injury prevention initiatives.* London: Health Development Agency, 2003.
62. Campbell DeLong Resources Inc. *Barriers to maintaining working smoke alarms: service area demographic study and interview research*. Portland, Oregan: Campbell DeLong Resources Inc., 2003.
63. Camit M. *Smoke alarms wake you up if there is a fire. Report on the Arabic Chinese and Vietnamese communities smoke alarm purchase and installation program 1998*. NSW Australia: NSW Multicultural Health Communication Service, 1998.
64. Camit M. Smoke alarms wake you up if there's a fire: a smoke alarm campaign targeting Arabic, Chinese, and Vietnamese communities in New South Wales. *Social Marketing Quarterly* 2002;8:52-54.
65. DiGuiseppi C, Slater S, Roberts I, Adams L, Sculpher M, Wade A, et al. The "Let's Get Alarmed!" initiative: a smoke alarm giveaway programme. *Inj Prev* 1999;5:177-82.
66. McConnell CF, Dwyer WO, Leeming FC. A behavioral approach to reducing fires in public housing. *J Community Psychol* 1996;24:201-12.
67. Roberts H, Curtis C, Liabo K, Rowland D, DiGuiseppi C, Roberts I. Putting public health evidence into practice: increasing the prevalence of working smoke alarms in disadvantaged inner city housing. *J Epidemiol Community Health* 2004;58:280-85.
68. Young M, Camit M, Mihajlovic M. A smoke alarm campaign in Arabic, Chinese and Vietnamese communities. *NSW Public Health Bulletin* 1999;10:133-35.
69. Thompson SG. Systematic review: why sources of heterogeneity in meta-analysis should be investigated. *BMJ* 1994;309:1351-5.

APPENDIX 1: METHODS USED IN THE PRODUCTION OF THE GUIDANCE

Literature Search

It was suggested by the funders that rather than reviewing the literature in this area systematically, the applicants could use their existing knowledge of this question to identify the relevant methodological literature relating to narrative synthesis of research based evidence. The team compiled an initial list of 52 works, but subsequently reviewed the decision to search in this way and initiated a limited literature search of key databases and the websites of relevant organisations. It was expected that this search might retrieve literature beyond that already identified, and that it would reduce bias introduced by a reliance on references known to the research team.

Furthermore, it was expected that relevant literature was more likely to be found in textbooks, reports and guidelines than in journal articles (it should be noted that many electronic bibliographic databases only index journals). A complication, which made devising a search strategy problematic, was the lack of a definitive terminology for 'narrative synthesis'. Searching for 'systematic review' or 'meta-analysis' would have been too sensitive and produced unmanageable results. An attempt was made to find definitions and search terms in the database indexes, textbooks and journal articles already identified, and through citation searches, but little additional information was found. There were many possible terms, none of which was consistently used.

Because of the difficulties encountered retrieving useful references from searching electronic databases, an internet search was also undertaken. It was expected that there would be more success looking at potentially relevant sites associated with conducting systematic reviews/meta-analyses, centres interested in evidence based health and social care research (and research methodology), health technology assessment organisations, and similar sites. Further searches of conference proceedings, reference lists, bibliographies and other resources identified while searching the Internet were undertaken. The public catalogues of the British Library and the Library of Congress were searched for general systematic review/meta-analysis books. Finally, a search using general search engines (Copernic and Google) and information gateway sites (OMNI and SOSIG) was undertaken.

Terminology

A number of books and journal articles were searched for definitions or descriptions of 'narrative synthesis'. Some citation searching was also undertaken. Terms found included 'realistic evaluation', 'realist synthesis', 'collective interpretation', 'interpretative synthesis', and 'explanatory synthesis', as well as methods of synthesis such as 'meta-ethnography' and 'triangulation'. The books and articles searched included the following:

- Busse R, Orvain J, Velasco M*, et al.* Best practice in undertaking and reporting health technology assessments. Working group 4 report. *Int J Technol Assess Health Care* 2002;**18**:361-422.

- Cooper H, Hedges LV. *The handbook of research synthesis*. New York: Rusell Sage Foundation, 1994.

- Egger M, Davey Smith G, Altman DG. *Systematic reviews in health care: meta analysis in context*. 2nd ed. London: BMJ Publishing, 2001.

- Forbes A, Griffiths P. Methodological strategies for the identification and synthesis of 'evidence' to support decision-making in relation to complex healthcare systems and practices. *Nurs Inq* 2002;**9**:141-55.

- Glasziou P, Irwig L, Bain C*, et al. Systematic reviews in health care: a practical guide*. Cambridge: Cambridge University Press, 2001.

- Khan KS, Ter Riet G, Glanville J*, et al. Undertaking systematic reviews of research on effectiveness: CRD's guidance for carrying out or commissioning reviews: CRD report 4*. 2nd ed. York: NHS Centre for Reviews and Dissemination, 2001.

- Mays N, Roberts E, Popay J. Synthesising research evidence. In: Fulop N, Allen P, Clarke A, Black N, editors. *Studying the organisation and delivery of health services: research methods*. London: Routledge, 2001.

- Williamson JW, Weir CR, Turner CW*, et al. Healthcare informatics and information synthesis; developing and applying clinical knowledge to improve outcomes*. Thousand Oaks, CA, USA: Sage Publications, 2002.

Internet searches

Websites on evidence-based policy and practice were browsed for publications, guidelines, ongoing research and other information of potential interest. Searches of organisation websites where search engines were available were undertaken using single terms and phrases such as 'narrative', 'synthesis' 'systematic review' or 'meta-analysis' or combinations of these. Anything of potential interest was added to an EndNote Library (bibliographic management software).

The sites searched, with dates and results were as follows:

Centre for Reviews and Dissemination (CRD).
University of York. 8th September 2003.
http://www.york.ac.uk/inst/crd/
Found: CRD Report 4 Undertaking systematic reviews of research on effectiveness: CRD's guidance for carrying out or commissioning reviews (2nd edition 2001) and useful links to other sites.

EPPI-Centre (Evidence for Policy and Practice Information Co-ordinating Centre).
SSRU ,University of London. 8th September 2003.
http://eppi.ioe.ac.uk/EPPIWeb/home.aspx
Found: A number of reviews about young people including methodological advances for using and synthesising qualitative data were found, the Centre's Review group manual was downloaded and some further useful links were noted.

Health Evidence Bulletins.
University of Wales, Cardiff. 8th September 2003.
http://hebw.uwcm.ac.uk/
Found: Project methodology was downloaded.

Scottish Intercollegiate Guidelines Network (SIGN).
Edinburgh. 8th September 2003.
http://www.sign.ac.uk/index.html
Found: SIGN 50. Guideline developer's handbook.

Agency for Healthcare Research and Quality (AHRQ).
U.S. Department of Health and Human Services. 8th September 2003.
http://www.ahcpr.gov/
Found: Reviews, assessments and guidelines were identified.

Aggressive Research Intelligence Facility (ARIF).
University of Birmingham. 8th September 2003.
http://www.bham.ac.uk/arif/
Found: Nothing of relevance was identified.

Bandolier.
Oxford. 8th September 2003.
http://www.jr2.ox.ac.uk/bandolier/index.html
Found: Nothing of relevance.

***Centre for Evidence-Based Medicine*.**
Oxford. 8th September 2003.
http://www.cebm.net/

Centre for Evidence-Based Child Health.
London. 8th September 2003.
http://www.ich.ucl.ac.uk/

Centre for Evidence-Based Mental Health.
Oxford. 8th September 2003.
http://www.cebmh.org

Centre for Evidence-Based Dentistry.
Oxford. 8th September 2003.
http://www.ihs.ox.ac.uk/cebd/index.htm

Centre for Evidence-Based Pharmacotherapy.
Birmingham. 8th September 2003.
http://www.aston.ac.uk/lhs/teaching/pharmacy/cebp/

Centre for Evidence-Based Nursing.
York. 8th September 2003.
http://www.york.ac.uk/healthsciences/centres/evidence/cebn.htm
Found: Nothing of relevance on any of these evidence-based centre sites was identified.

Guidelines International Network. (G-I-N).
8th September 2003.
http://www.g-i-n.net/index.cfm?fuseaction=home
Found: Nothing of relevance was identified, though this is a new organisation and website with little content as yet.

Health Development Agency.
London. 8th September 2003.
http://www.hda-online.org.uk/
Found: In the Evidence Based Medicine section of the site papers about good quality evidence, presentations by Hammersley and Marks and process and policy paper including a section on 'synthesising the evidence'.
Health Information Research Unit (HIRU).
McMaster University. 8th September 2003.
http://hiru.mcmaster.ca/default.htm
Found: Nothing of relevance was identified.

National electronic Library for Health (NeLH).
8th September 2003.
http://www.nelh.nhs.uk/
Found: Nothing of relevance was identified.

National Institute of Clinical Excellence (NICE).
London. 8th September 2003.
http://www.nice.org.uk/cat.asp
Found: Nothing of relevance was identified. The guidelines for undertaking technology assessments were not available online.

International Network of Agencies for Health Technology Assessment (INAHTA).
8th September 2003.
http://www.inahta.org/
Found: Nothing of relevance was found on the INAHTA site, or on any of the member sites. Any guides or guidelines about conducting health technology assessment did not describe how to undertake narrative synthesis. However searching on a number of the sites was problematic because we could only search in English.

INAHTA member sites searched:

- Australia (ASERNIP, MSAC)
- Austria (ITA)
- Canada (AETMIS, AHFMR, CCOHTA)
- Chile (ETESA)
- Cuba (INHEM)
- Denmark (DACEHTA, DSI)
- Finland (FinOHTA)
- France (ANAES, CEDIT)
- Germany (DIMDI)
- Netherlands (CVZ, GR, TNO, ZonMW)
- New Zealand (NZHTA)
- Norway (SMM)
- Spain (AETS, AETSA, CAHTA, OSTEBA)
- Sweden (CMT, SBU)
- Switzerland (SNHTA, TA-SWISS)

School of Health and Related Research (ScHARR) – Netting the Evidence.
University of Sheffield. 8th September 2003.
http://www.nettingtheevidence.org.uk/
Found: Nothing of relevance was identified.

King's Fund.
London. 8th September 2003.
http://www.kingsfund.org.uk/
Found: Nothing of relevance was identified.

OMNI (Organising Medical Networked Information).
8th September 2003.
http://omni.ac.uk/
Internet gateway to evaluated, quality Internet resources in health and medicine. Searched for 'narrative synthesis', 'synthesis' 'systematic review' and 'meta-analysis'. Nothing of relevance was identified.

TRIP Database (Turning Research into Practice).
8th September 2003.
http://www.update-software.com/trip/about.htm
Searched for 'narrative synthesis', 'synthesis' 'systematic review' and 'meta-analysis'. Nothing of relevance was identified.

Evidence Network.
The Economic and Social Research Council (ESRC) site for Evidence Based Policy and Practice Research in the UK. 11th September 2003.
http://www.evidencenetwork.org/home.asp
Found: A number of potentially relevant publications were found.
The websites of the seven research teams involved in the Network were also searched individually with a couple of additional publications of interest being identified. (What works for Children, Centre for Neighbourhood Research, Centre for Evidence-Based Public Health Policy, Centre for Economic Evaluation, Research Unit for Research Utilisation, Centre for Evidence in Ethnicity, Health and Diversity, and the Centre for Comparative European Policy Evaluation).

Health Care Practice Research and Development Unit (HCPRDU).
University of Salford. 11th September 2003.
http://www.fhsc.salford.ac.uk/hcprdu/
Found: A section on systematic reviewing was downloaded, and reference to 2 reviews unavailable online, including narrative syntheses of the evidence was noted.

Social Care Institute for Excellence (SCIE).
London. 11th September 2003.
http://www.scie.org.uk/
Found: Nothing of relevance was identified.

electronic Library for Social Care (eLSC).
11th September 2003.
http://www.elsc.org.uk/
Found: The site houses the CareData bibliographic database, which was searched and identified a number of useful references. (See database searches below).

Centre for Evidence-Based Social Services.
University of Exeter. 11th September 2003.
http://www.ex.ac.uk/cebss/
Found: Nothing of relevance was identified.

The Qual-Quan Evidence Synthesis Group.
University of Leicester. 11th September 2003.
http://www.prw.le.ac.uk/research/qualquan/index.htm
Found: Reference to a number of ongoing projects was found and completed publications about the synthesis of qualitative and quantitative data in systematic reviews were downloaded.

The Campbell Collaboration.
11th September 2003.
http://www.campbellcollaboration.org/
Found: Guidelines for the preparation of reviews, presentations given at the 2003 Campbell Colloquium, newsletters of the Campbell Methods Group, and protocols and references via the Cochrane Qualitative Research Methods Group and Campbell Process Implementation Methods Group were downloaded. A methods protocol was found on the Reviews database (C2-RIPE), but nothing specifically about narrative synthesis was found on the trials register (C2-SPECTR).

The Cochrane Collaboration.
11th September 2003.
http://www.cochrane.de/beta/index0.htm
Found: Cochrane Reviewers Handbook version 4.2 was identified. We looked at all 50 Cochrane Review Group websites for any additional guidance to the Reviewers Handbook.
See also searches of the Cochrane Library Methodology Register and *Conference Proceedings* below.

SOSIG (The Social Science Information Gateway).
11th September 2003.
http://www.sosig.ac.uk/
Internet gateway to evaluated, quality Internet resources in the social sciences, business and law. Searched for ‘narrative synthesis’, ‘synthesis’ ‘systematic review’ and ‘meta-analysis’. Nothing of relevance was identified.

British Library Public Catalogue.
11th September 2003.
http://blpc.bl.uk/
Searched in title and abstract for ‘narrative synthesis’, ‘systematic review’ and ‘meta-analysis’. The bibliographic details of any that looked of potentially relevant were retained.

Library of Congress Online Catalog.
11th September 2003.
http://www.loc.gov/
Searched for ‘narrative synthesis’, ‘systematic review’ and ‘meta-analysis’. Almost all potentially relevant titles had already been found in the British Library Catalogue search. One reference of potential interest was retained.

Copernic (meta-search engine).
12th September 2003.
http://www.copernic.com
Searched for 'narrative synthesis', 'systematic review' and 'meta-analysis'. Browsed through hits, but found nothing of relevance that had not already been identified.

Google (general search engine).
12th September 2003.
http://www.google.com
Searched for 'narrative synthesis', 'systematic review' and 'meta-analysis'. Browsed through the first 100 hits, but found nothing of relevance that had not already been identified.

Any references of potential interest were saved and details added to an EndNote Library.

Additional Internet searches

Additional Internet sites were searched. These were identified from previous searches or were suggested by members of the project team.

If anything of relevance was found the details were added to the Internet Endnote library.

Additional websites were searched on 22nd September 2003 and included:
Joanna Briggs Institute (http://www.joannabriggs.edu.au/about/home.php), US Preventive Services Task Force (http://www.ahcpr.gov/clinic/uspstfix.htm), Canadian Task Force on Preventive Health Care (http://www.ctfphc.org/), AHRQ Evidence Based Practice Centers (http://www.ahcpr.gov/clinic/epc/), Alberta Heritage Foundation for Medical Research (http://www.ahfmr.ab.ca/).

Database searches

Although it was recognised that database searching would be difficult, a number of electronic databases were searched. The strategies used were fairly limited in the use of search terms because of the lack of definitive terms for 'narrative synthesis'. Ideally a search for 'synthesis' alone would have retrieved further relevant records, but would also have resulted in an unmanageable number of irrelevant references. Even combining 'synthesis' with terms used to identify systematic reviews/meta-analyses would have produced a large number of references (again almost entirely irrelevant). This is a problem experienced when searching for any reports of methodological research, but particularly in this case where there is no definitive terminology.

The databases, strategies, dates and results of the database searches were as follows:

MEDLINE: Ovid Gateway. Internet. 1966-2003/August week 4. 9th September 2003.
The MEDLINE search covered the date range 1966 to August 2003. 347 records were identified.
1. review.ab.
2. review.pt.
3. meta-analysis.ab.
4. meta-analysis.pt.
5. meta-analysis.ti.
6. 1 or 2 or 3 or 4 or 5
7. letter.pt.
8. editorial.pt.
9. comment.pt.
10. 7 or 8 or 9
11. 6 not 10
12. (narrativ$ adj3 (synth$ or summar$ or description$ or analy$ or finding$ or form or forms))
13. realistic evaluation$
14. collective interpret$
15. meta ethnograp$
16. meta stud$

17. grounded theory
18. realist synth
19. interp$ synth
20. meta synth$
21. (meta matrix) or (meta matrices)
22. mini synth$
23. explanatory synth$
24. triangulation
25. theory led
26. bayesian adj2 hierarch$
27. or/12-26
28. 11 and 27

Sociological Abstracts: WebSPIRS. Internet. 1963-2003/6. 9th September 2003.
The Sociological Abstracts search covered the date range 1963 to June 2003. 92 records were identified.

#1 review in ti,ab,de
#2 meta analy*
#3 #1 or #2
#4 narrative near3 (synth* or summar* or analy* or description* or finding* or form or forms)
#5 realistic evaluation*
#6 collective interp*
#7 meta ethnograp*
#8 meta stud*
#9 grounded theory
#10 realist synth*
#11 interp* synth*
#12 meta synth*
#13 mini synth*
#14 explanatory synth*
#15 triangulation
#16 (meta matrix) or (meta matrices)
#17 theory led
#18 bayesian near3 hierarch*
#19 #4 or #5 or #6 or #7 or #8 or #9 or #10 or #11 or #12 or #13 or #14 or #15 or #16 or #17 or #18
#20 #3 and #19

Social Science Citation Index (SSCI): Web of Science. Internet. 1981-2003/8. 9th September 2003.
The SSCI search covered the date range 1981 to August 2003. 195 records were identified.
TS=metaanalysis
TS=meta analysis
TS=systematic SAME TS=review*
TS=systematic SAME TS=overview*
TS=literature SAME TS=review*
#1 or #2 or #3 or #4 or #5
TS=narrative SAME TS=synth*
TS=narrative SAME (TS=summar* or TS=description*)
TS=narrative SAME (TS=finding* or TS=review*)
TS=narrative SAME (TS=form or TS=forms)
TS=meta SAME (TS=ethnography OR TS=synthesis OR TS=study)
(TS=realistic evaluation) or (TS=collective interp*)
TS=synthesis SAME (TS=interp* OR TS=explanatory)
TS=synthesis SAME (TS=mini OR TS=realist)
TS=grounded theory
(TS=meta matrix) or (TS=theory led)
TS=bayesian SAME TS=hierarch*

TS=triangulation
#7 or #11 or #8 or #9 or #10 or #11 or #12 or #13 or #14 or #15 or #16 or #17 or #18
#6 and #19

PsycINFO: BIDS. Internet. 1872-2003/9. 9th September 2003.
The PsycINFO search covered the date range 1872 to September 2003. This search identified 352 records.
#1 META-ANALYSIS in PT
#2 LITERATURE-REVIEW-RESEARCH-REVIEW in PT
#3 metaanaly* in ti,de
#4 meta-analy* in ti,de
#5 (review* or overview*) in ti
#6 (review literature) in ti
#7 synthes* near3 ((literature* or research or studies or data) in ti)
#8 ((review* or overview*) in ti) near10 ((systematic* or methodologic* or quantitativ* or research* or literature or studies or trial* or effective*) in ti)
#9 #1 or #2 or #3 or #4 or #5 or #6 or #7 or #8
#10 narrative near3 (synth* or summar* or analy* or description* or finding* or form or forms)
#11 realistic evaluation*
#12 collective interpret*
#13 meta ethnograp*
#14 meta stud*
#15 grounded theory
#16 realist synth*
#17 interp* synth*
#18 meta synth*
#19 mini synth*
#20 explanatory synth*
#21 triangulation
#22 (meta matrix) or (meta matrices)
#23 theory led
#24 bayesian near3 hierarch*
#25 #10 or #11 or #12 or #13 or #14 or #15 or #16 or #17 or #18 or #19 or #20 or #21 or #22 or #23 or #24
#26 #9 and #25

Cochrane Library Methodology Register: Internet. 2003:Issue 3. 9th September 2003.
The Cochrane Library Methodology Register search identified 8 records.
#1 (narrativ* next synth*)
#2 (narrativ* next summar*)
#3 (narrativ* next description*)
#4 (narrativ* next finding*)
#5 (narrativ* next form*)
#6 (realistic next evaluation*)
#7 (collective next interp*)
#8 (meta next ethnograp*)
#9 (grounded next theory)
#10 (realist next synth*)
#11 (interp* next synt*)
#12 (meta next synth*)
#13 (mini next synth*)
#14 (explanatory next synth*)
#15 (#1 or #2 or #3 or #4 or #5 or #6 or #7 or #8 or #9 or #10 or #11 or #12 or #13 or #14)

CareData. Internet. 9th September 2003.
http://www.elsc.org.uk/caredata/caredata.htm
CareData produced 23 unique records. The search interface available for CareData does not allow for sophisticated search strategies. Separate phrase searching was undertaken, firstly in the abstract field and then in the keyword field. The phrases searched in the abstract field were 'narrative

synthesis', 'synthesis' 'systematic review' and 'meta analysis'. The keyword field had an index and the terms 'literature review' and 'research methods' were combined. The results of the 5 separate searches were pooled, and the duplicate references removed.

DARE (Database of Abstracts of Reviews of Effects): Internal CRD administration database. CAIRS T System. 1994-2003/8. 9th September 2003.

The internal CRD administration version of DARE was searched for methodology papers identified as part of the DARE production process, and CRD records which are not available on the public DARE database. This search identified 32 records.

s narrativ$(w3)(synth$ or summar$ or description$ or analy$ or finding$ or form or forms or review$)
s realistic(w)evaluation$
s collective(w)interpret$
s meta(w)ethnograp$
s meta(w)stud$
s grounded(w)theory
s realist(w)synth$
s interp$(w)synth$
s meta(w)synth$
s mini(w)synth$
s explanatory(w)synth$
s theory(w)led
s (meta(w)matrix) or (meta(w)matrices)
s triangulation
s Bayesian(w3)hierarch$
s s1 or s2 or s3 or s4 or s5 or s6 or s7 or s8 or s9 or s10 or s11 or s12 or s13 or s14 or s15
s m/st1
s s16 and s17

Applied Social Sciences Index and Abstracts (ASSIA): Cambridge Scientific Abstracts (CSA). Internet. 1987-2003. 9th September 2003.

The ASSIA search covered the date range 1987 to date. The search identified 55 records ((synthesis) OR (narrative)) AND ((systematic review) OR (meta analysis) OR KW=(systematic reviews) OR (meta analysis))

Educational Resources Information Center (ERIC): Dialog. Internet. 1966-2003/6. 9th September 2003.

The ERIC search covered the date range 1966 to June 2003. The search identified 176 records. EXPLANATORY SYNTH? OR MINI SYNTH? OR META SYNTH? OR INTERP? SYNTH? OR REALIST SYNTH? OR GROUNDED THEORY OR META STUD? OR META ETHNOGRAP? OR COLLECTIVE INTERPRET? OR REALISTIC EVALUATION? OR NARRATIV? WITH(3) (SYNTH? OR SUMMAR? OR DESCRIPTION? OR ANALY? OR FINDING? OR FORM OR FORMS OR REVIEW?) AND META ANALYSIS OR 1 term(s): ERIC Subject Headings=("META ANALYSIS") OR REVIEW

All references were downloaded into an EndNote Library and deduplicated.

Additional database searches

The search strategies were rerun to include further search terms on 21st October 2003. The new search terms were derived from references identified by the original searches or by reviewer hand searches. The following search terms were added to each strategy:
'Cross design', 'evaluation synthesis', 'descriptive synthesis' and 'best evidence synthesis'.

The updated Medline search strategy as an example is presented below. The other database strategies were amended in similar ways.

MEDLINE: Ovid.

1. review.ab.
2. review.pt.
3. meta-analysis.ab.
4. meta-analysis.pt.
5. meta-analysis.ti.
6. 1 or 2 or 3 or 4 or 5
7. letter.pt.
8. editorial.pt.
9. comment.pt.
10. 7 or 8 or 9
11. 6 not 10
12. (narrativ$ adj3 (synth$ or summar$ or description$ or analy$ or finding$ or form or forms))
13. realistic evaluation$
14. collective interpret$
15. meta ethnograp$
16. meta stud$
17. grounded theory
18. realist synth
19. interp$ synth
20. meta synth$
21. (meta matrix) or (meta matrices)
22. mini synth$
23. explanatory synth$
24. triangulation
25. theory led
26. bayesian adj2 hierarch$
27. cross design$
28. evaluation synth$
29. (best evidence adj3 synth$)

30. descrip$ synth$.mp
31. or/12-30
32. 11 and 31

The results of the searches were added to an Endnote library and then deduplicated against the original search results. A further 192 references were identified from all the databases using these additional terms.

Conference Proceedings
A number of conference proceedings were handsearched, and any potentially relevant abstracts were added to the EndNote Library. The following conference proceedings were searched:

- *19th Annual Meeting of the International Society of Technology Assessment in Health Care*; 2003 Jun 22-25; Canmore, Canada.
- *18th Annual Meeting of the International Society of Technology Assessment in Health Care*; 2002 Jun 9-12; Berlin, Germany.
- *17th Annual Meeting of the International Society of Technology Assessment in Health Care*; 2001 Jun 3-6; Philadelphia, USA.
- *16th Annual Meeting of the International Society of Technology Assessment in Health Care*; 2000 Jun 18-21; The Hague, The Netherlands.
- *15th Annual Meeting of the International Society of Technology Assessment in Health Care*; 1999 Jun 20-23; Edinburgh, Scotland.
- *14th Annual Meeting of the International Society of Technology Assessment in Health Care*; 1998 Jun 7-10; Ottawa, Canada.
- *13th Annual Meeting of the International Society of Technology Assessment in Health Care*; 1997 May 25-28; Barcelona, Spain.
- *12th Annual Meeting of the International Society of Technology Assessment in Health Care*; 1996 Jun 23-26; San Francisco, USA.
- *11th Annual Meeting of the International Society of Technology Assessment in Health Care*; 1995 Jun; Stockholm, Sweden.
- *10th Annual Meeting of the International Society of Technology Assessment in Health Care*; 1994; Baltimore, USA.
- *4th Symposium on Systematic Reviews: Pushing the Boundaries*; 2002 Jul 2-4; Oxford, UK.
- *3rd Symposium on Systematic Reviews*; 2000 Jul 3-5 Oxford, UK.
- *2nd Symposium on Systematic Reviews: Beyond the Basics*; 1999 Jan 5-7; Oxford, UK.
- *1st Symposium on Systematic Reviews: Beyond the Basics*; 1998 Jan 8-9; Oxford, UK.
- *11th Cochrane Colloquium: Evidence, health care and culture*; 2003 Oct 26-31; Barcelona, Spain.
- *10th Cochrane Colloquium*; 2002 31 Jul-3 Aug; Stavanger, Norway.
- *9th Cochrane Colloquium: The evidence dissemination process: how to make it more efficient*; 2001 Oct 9-13; Lyon, France.

- *8th Cochrane Colloquium: Evidence for action: challenges for the Cochrane Collaboration in the 21st century*; 2000 Oct 25-29; Cape Town, South Africa.

- *7th Cochrane Colloquium: The best evidence for healthcare: the role of the Cochrane Collaboration*; 1999 Oct 5-9; Rome, Italy.

- *6th Cochrane Colloquium: Systematic reviews: evidence for action*; 1998 Oct 22-26; Baltimore, USA.

- *5th Cochrane Colloquium: Using the evidence*; 1997 Oct 8-12; Amsterdam, Holland.

- *4th Cochrane Colloquium*; 1996; Adelaide, Australia.

- *3rd Cochrane Colloquium*; 1995 Oct 4-8; Oslo, Norway.

- *2nd Cochrane Colloquium*; 1994; Hamilton, Canada

Methods texts selection process

A total of 1,307 articles were retrieved from the literature searches. Two reviewers independently selected articles from the titles and abstracts available from the searches. Articles were included if they offered guidance on the conduct of reviews or combining data from different studies. Where reviewers disagreed, the full article was included for further investigation. This resulted in a total of 260 full publications being ordered for further assessment. One reviewer then selected all published articles that reported any tool or technique meeting the following criteria:

1) Was concerned with the synthesis of primary research
2) Was not a strictly statistical technique (e.g. meta-analysis)
3) Could conceivably be applied or adapted to the context of a systematic review of the literature.

A total of 69 studies were selected on the basis of these criteria, and were used to inform our guidance.

The majority of included articles were initially identified from the internet searches (36%) and database searches (33%) (see figure 1.1). Thirteen (19%) of the initial texts identified by the project team were included in the final 66 selected articles. A further eight articles (12%) were identified by handsearching/scanning of reference lists.

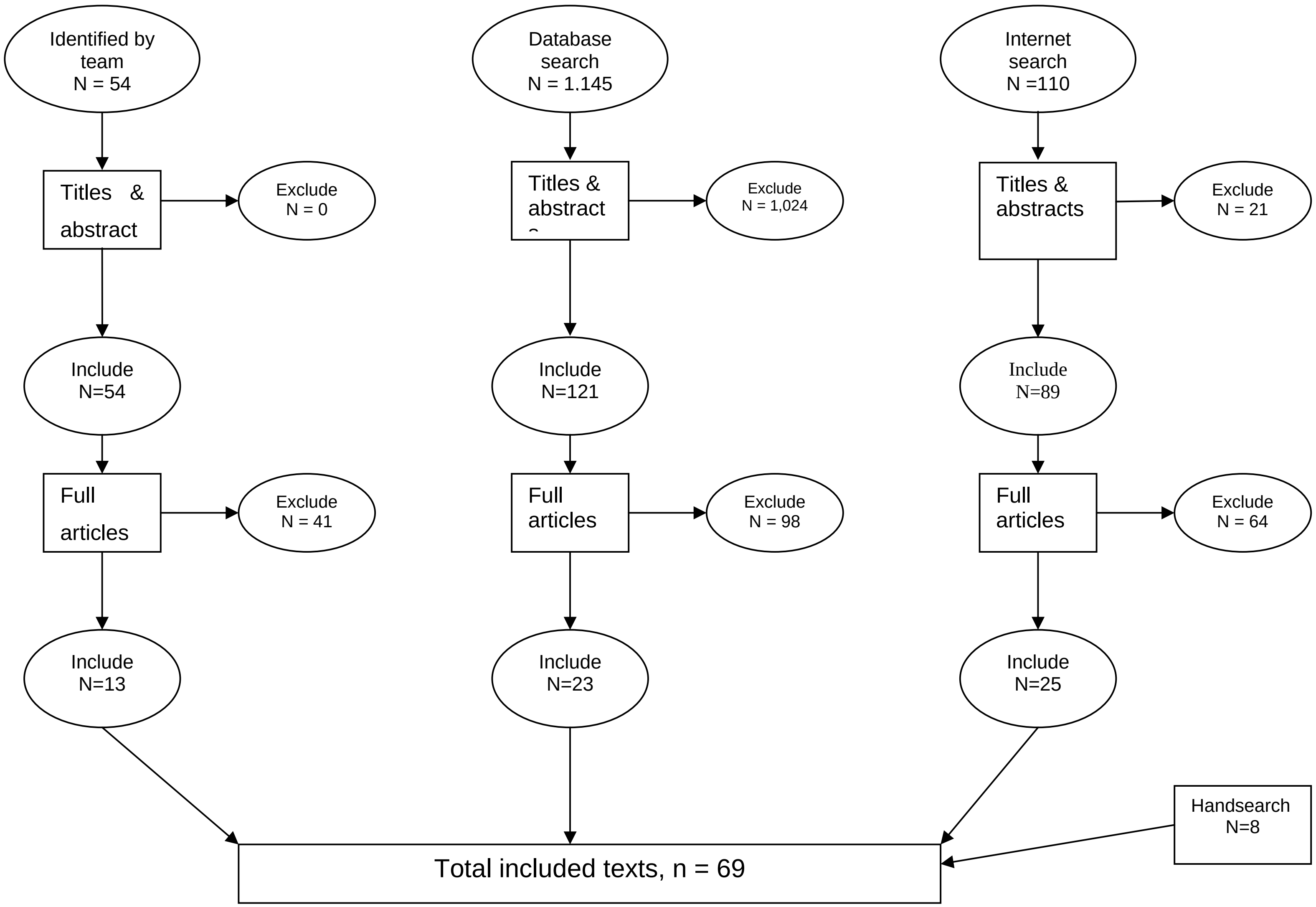

Figure 1.1: Methods text selection process.

APPENDIX 2: BIBLIOGRAPHY OF METHODOLOGICAL TEXTS USED IN THE PRODUCTION OF THE GUIDANCE

Extracted methods texts

1. Bangert Drowns RL, Wells Parker E, Chevillard I. Assessing the methodological quality of research in narrative reviews and meta-analyses. In: Bryant KJ, Windle M, editors. *The science of prevention: methodological advances from alcohol and substance abuse research*. Washington, DC, US: American Psychological Association, 1997. p. 405-429.

2. Barbour RS, Barbour M. Evaluating and synthesizing qualitative research: the need to develop a distinctive approach. *J Eval Clin Pract* 2003;9:179-186.

3. Beck CT. Mothering multiples: a meta-synthesis of qualitative research. *MCN, American Journal of Maternal Child Nursing.* 2002;27:214-21.

4. Becker BJ. Examining theoretical models through research synthesis: the benefits of model driven meta-analysis. *Eval. Health Prof.* 2001;24:190-217.

5. Begley CM. Using triangulation in nursing research. *J Adv Nurs* 1996;24:122-8.

6. Bennett JA. A case for theory triangulation. *Nursing Science Quarterly.* 1997;10:97-102; discussion 103-6.

7. Brannen J. *Mixing methods: qualitative and quantitative research*. Aldershot: Avebury, 1992.

8. Britten N, Campbell R, Pope C, Donovan J, Morgan M, Pill R. Using meta ethnography to synthesise qualitative research: a worked example. *J Health Serv Res Policy* 2002;7:209-15.

9. Burls A, Cummins C, Fry-Smith A, Gold L, Hyde C, Jordan R, et al. *West Midlands Development and Evaluation Service handbook [monograph online]*. Birmingham: West Midlands Development and Evaluation Service, 2000. Available from: http://www.publichealth.bham.ac.uk/wmhtac/pdf/wmhandbook.pdf

10. Busse R, Orvain J, Velasco M, Perleth M, Drummond M, Gurtner F, et al. Best practice in undertaking and reporting health technology assessments. Working group 4 report. *Int J Technol Assess Health Care* 2002;18:361-422.

11. Campbell R, Pound P, Pope C, Britten N, Pill R, Morgan M, et al. Evaluating meta-ethnography: a synthesis of qualitative research on lay experiences of diabetes and diabetes care. *Soc. Sci. Med.* 2003;56:671-84.

12. Clinkenbeard PR. Beyond summary: constructing a review of the literature. In: Buchanan NK, Feldhusen JF, editors. *Conducting research and evaluation in gifted education: a handbook of methods and applications*. New York, NY: Teachers College Press, 1991. p. 33-50.

13. The Cochrane Collaboration. *Cochrane reviewers' handbook (Version 4.2.0: March, 2003) [monograph online]*, 2003. Available from: http://www.cochrane.dk/cochrane/handbook/hbook.htm

14. Cook TD, Leviton LC. Reviewing the literature: a comparison of traditional methods versus meta-analysis. *J Pers* 1980;48:449-72.

15. Cooper H, Hedges LV, editors. *The handbook of research synthesis*. New York: Russell Sage Foundation, 1994.

16. Curlette WL, Cannella KS. Going beyond the narrative summarization of research findings: the meta-analysis approach. *Research in Nursing & Health.* 1985;8:293-301.

17. Dixon-Woods M, Fitzpatrick R, Roberts KA. Including qualitative research in systematic reviews: opportunities and problems. *J Eval Clin Pract* 2001;7:125-133.

18. Eaves YD. A synthesis technique for grounded theory data analysis. *J Adv Nurs* 2001;35:654-63.

19. EPPI Centre. *EPPI-Centre Review Group Manual: working document version 1.1 [monograph online]*. London: EPPI Centre, 2001. Available from: http://eppi.ioe.ac.uk/EPPIWebContent/downloads/RG_manual_version_1_1.pdf

20. Evans D. Systematic reviews of interpretive research: interpretive data synthesis of processed data. *Australian Journal of Advanced Nursing* 2002;20:22-6.

21. Flinspach SL. Interpretive synthesis: a methodology for reviewing qualitative case-study research. *Dissertation Abstracts International, A: The Humanities and Social Sciences* 2001;61:4971-A.

22. Forbes A, Griffiths P. Methodological strategies for the identification and synthesis of 'evidence' to support decision-making in relation to complex healthcare systems and practices. *Nurs Inq* 2002;9:141-55.

23. Foster RL. Addressing epistemologic and practical issues in multimethod research: a procedure for conceptual triangulation. *Advances in Nursing Science* 1997;20:1-12.

24. Fulop N, Allen P, Clarke A, Black N. *Studying the organisation and delivery of health services: research methods*. London: Routledge, 2001.

25. Jensen LA, Allen MN. Meta-synthesis of qualitative findings. *Qualitative Health Research* 1996;6:553-60.

26. Jones K. *Mission drift in qualitative research, or moving toward a systematic review of qualitative studies, moving back to a more systematic narrative review [monograph online]*. Leicester: Centre for Evidence in Ethnicity, Health & Diversity, De Montfort University, 2003. Available from: http://users.wbs.warwick.ac.uk/cms_attachment_handler.cfm?f=d250896e-82a2-48e6-b356-c310d3be6861&t=mission_drift.pdf

27. Light RJ, Pillemer DB. *Summing up: the science of reviewing research*. Cambridge, MA, USA: Harvard University Press, 1984.

28. Long TJ, Convey JJ, Chwalek AR. *Completing dissertations in the behavioral sciences and education: a systematic guide for graduate students*. San Francisco: Jossey-Bass, 1985.

29. Maggs-Rapport F. Combining methodological approaches in research: ethnography and interpretive phenomenology. *J Adv Nurs* 2000;31:219-25.

30. Mulrow C, Cook D. *Systematic reviews: synthesis of best evidence for health care decisions*. Philadelphia: American College of Physicians, 1998.

31. Khan KS, Ter Riet G, Glanville J, Sowdon AJ, Kleijnen J. *Undertaking systematic reviews of research on effectiveness: CRD's guidance for carrying out or commissioning reviews: CRD report 4*. 2nd ed. York: NHS Centre for Reviews and Dissemination, 2001.

32. Noblit GW, Hare RD. Meta-ethnography: issues in the synthesis and replication of qualitative research. In: *Annual Meeting of the American Educational Research Association*; 1983 Apr 11-15; Montreal, Quebec. 1983. p. 23.

33. Noblit GW, Hare RD. *Meta-ethnography: synthesizing qualitative studies*. London: Sage, 1988.

34. Nurius PS, Yeaton WH. Research synthesis reviews: an illustrated critique of "hidden" judgments, choices, and compromises. *Clin Psychol Rev* 1987;7:695-714.

35. Pawson R. Evidence-based policy: the promise of realist synthesis. *Evaluation* 2002;8:340-358.

36. Popay J, Rogers A, Williams G. Rationale and standards for the systematic review of qualitative literature in health services research. *Qualitative Health Research* 1998;8:341-51.

37. Ragin CC. *The comparative method: moving beyond qualitative and quantitative strategies*. Berkeley, CA: University of California Press, 1987.

38. Rees R, Harden A, Shepherd J, Brunton G, Oliver S, Oakley A. *Young people and physical activity: a systematic review of research on barriers and facilitators [monograph online]*. London: EPPI Centre, 2001. Available from: http://eppi.ioe.ac.uk/EPPIWebContent/hp/reports/physical_activity01/physical_activity.pdf

39. Risjord MW, Dunbar SB, Moloney MF. A new foundation for methodological triangulation. *J Nurs Scholarsh* 2002;34:269-75.

40. Shepherd J, Harden A, Rees R, Brunton G, Garcia J, Oliver S, et al. *Young people and healthy eating: a systematic review of research on barriers and facilitators [monograph online]*. London: EPPI Centre, 2001. Available from: http://eppi.ioe.ac.uk/EPPIWebContent/hp/reports/health_eating01/healthy_eating_yp.pdf

41. Slavin RE. Best-evidence synthesis: an alternative to meta-analytic and traditional reviews. In: Shadish WRJ, Reichardt CS, editors. *Evaluation studies: review annual*. Thousand Oaks, CA: Sage Publications, Inc., 1987. p. 667-673.

42. Slavin RE. Best evidence synthesis: an intelligent alternative to meta-analysis. *J Clin Epidemiol* 1995;48:9-18.

43. Suri H, Clarke D. Revisiting methods of literature synthesis. In: *Annual Meeting of the American Educational Research Association*; 1999 Apr 19-23; Montreal, Canada. 1999. p. 2-15.

44. Suri H. The process of synthesising qualitative research: a case study. In: *Annual Conference of the Association for Qualitative Research*; 1999 Jul 6-10; Melbourne. 1999. Available from: http://www.latrobe.edu.au/aqr/offer/papers/HSuri.htm

45. Suri H. A methodologically inclusive model for research synthesis [monograph online]. In: *Australian Association for Research in Education (AARE) and the New-Zealand Association for Research in Education (NZARE) Joint-Conference*; 1999 Dec; Melbourne. 1999. Available from: http://www.aare.edu.au/99pap/sur99673.htm

46. Suri H. A critique of contemporary methods of research synthesis. *Post-Script* 2000;1:49-55. Available from: http://www.edfac.unimelb.edu.au/student/insight/postscriptfiles/vol1/suri.pdf

47. Sutton A, Abrams KR, Jones DR, Sheldon TA, Song F. Systematic reviews of trials and other studies. *Health Technol Assess* 1998;2.

48. Thomas J, Harden A, Oakley A, Oliver S, Sutcliffe K, Rees R, et al. Integrating qualitative research with trials in systematic reviews. *BMJ* 2004;328:1010-1012.

49. Williamson JW, Weir CR, Turner CW, Lincoln MJ, Cofrin KMW. *Healthcare informatics and information synthesis; developing and applying clinical knowledge to improve outcomes*. Thousand Oaks, CA, USA: Sage Publications, 2002.

50. Yin RK, Heald KA. Using the case survey method to analyze policy studies. *Administrative Science Quarterly* 1975;20:371-81.

Methods texts covering material similar or identical to previously extracted texts

1. ACC/AHA Task Force on Practice Guidelines. Section II: tools and methods for creating guidelines. Step four: synthesize and interpret the evidence [monograph online]. In: *Manual for ACC/AHA Guideline Writing Committees.Methodologies and Policies from the ACC/AHA Task Force on Practice Guidelines*. Bethesda, MD: American College of Cardiology, American Heart Association, 2002. Available from: http://www.acc.org/clinical/manual/manual_IIstep4.htm

2. Booth A. Cochrane or cock-eyed? How should we conduct systematic reviews of qualitative research? [monograph online]. In: *Qualitative Evidence-Based Practice Conference*; 2001 May 14-16; Coventry University. 2001. Available from: http://www.leeds.ac.uk/educol/documents/00001724.htm

3. Bryman A. *Social research methods*. Oxford: Oxford University Press, 2001.

4. Canadian Task Force on Preventive Health Care. *Canadian Task Force Methodology [monograph online]*. London, Ontario, Canada: Canadian Task Force on Preventive Health Care, 1998. Available from: http://www.ctfphc.org/Methodology.html

5. Smith MC, Stullenbarger E. A prototype for integrative review and meta-analysis of nursing research. *J Adv Nurs* 1991;16:1272-83.

6. Gall MD, Borg WR, Gall JP. *Educational research. an introduction*. 7th ed. Boston, MA: Allyn & Bacon, 2003.

7. Gough D, Elbourne D. Systematic research synthesis to inform policy, practice and democratic debate. *Social Policy and Society* 2002;1:225-236.

8. Green BN, Johnson CD, Adams A. Writing narrative literature reviews for peer-reviewed journals: secrets of the trade. *Journal of Sports Chiropractic & Rehabilitation* 2001;15:5-19.

9. Letzel H. 'Best evidence synthesis:an intelligent alternative to meta-analysis': discussion. a case of "either or" or "as well" (comment). *J Clin Epidemiol* 1995;48:19-21.

10. Pawson R. *Assessing the quality of evidence in evidence-based policy: why, how and when? Draft [monograph online]*. London: ESRC, 2003. Available from: http://www.ccsr.ac.uk/methods/publications/Pawson.pdf

11. Pawson R. Evidence-based policy: in search of a method. *Evaluation* 2002;8:157-181.

12. Pearson A, Wiechula R, Long L. QARI: a systematic approach to the appraisal, extraction and synthesis of the findings of qualitative research [abstract]. In: *11th Cochrane Colloquium: Evidence, health care and culture*; 2003 Oct 26-31; Barcelona, Spain. 2003.

13. Pearson A, Wiechula R, Long L. The role of text in enhancing Cochrane reviews of effectiveness [abstract]. In: *11th Cochrane Colloquium: Evidence, health care and culture*; 2003 Oct 26-31; Barcelona, Spain. 2003.

14. Swann C, Falce C, Morgan A, Kelly M. *HDA Evidence Base: process and quality standards manual for evidence briefings. Draft [monograph online]*. London: Health Development Agency Public Health Evidence Steering Group, 2002. Available from: http://www.hda.nhs.uk/evidence/ebmanual.pdf

15. Thomas J, Harden A, Sutcliffe K, Oakley A, Rees R, Oliver S, et al. New methods for integrating qualitative and quantitative research in a systematic review: an example from public health [abstract]. In: *11th Cochrane Colloquium: Evidence, health care and culture*; 2003 Oct 26-31; Barcelona, Spain. 2003.

Methods texts not yet received/extracted

1. Cwikel J, Behar L, Rabson-Hare J. A comparison of a vote-count and meta-analysis review of intervention research with adult cancer patients. *Res Soc Work Pract* 2000;10:139-158.

2. Edwards A, Russell I, Stott N. Signal versus noise in the evidence base for medicine: an alternative to hierarchies of evidence? *Fam Pract* 1998;15:319-322.

3. Tashakkori A, Teddlie C. *Mixed Methodologogy: combining qualitative and quantitative approaches*. Thousand Oaks, CA: Sage Publications, 1998.

4. Wachter KW, Straf ML, editors. *The future of meta-analysis*. New York, NY, US: Russell Sage Foundation, 1990.
