# Supplementary material for: The NRF2 transcription factor plays a dual role in colorectal cancer: A systematic review
Source: PLoS One. 2017 May 18;12(5):e0177549. doi: 10.1371/journal.pone.0177549 (PMC5436741; doi:10.1371/journal.pone.0177549)
Supplement: S1 Table — (DOCX) [file pone.0177549.s003.docx]

S1 Table. Excluded reports which not keep the inclusion criteria.

| **Title** | **Reason for exclusion** |
| --- | --- |
| Anti-inflammatory/Anti-oxidative stress activities and differential regulation of Nrf2-mediated genes by non-polar fractions of tea Chrysanthemum zawadskii and licorice Glycyrrhiza uralensis. | Does not discuss CRC |
| Coffee constituents as modulators of Nrf2 nuclear translocation and ARE (EpRE)-dependent gene expression. | Does not discuss CRC |
| Dietary squalene supplementation improves DSS-induced acute colitis by downregulating p38 MAPK and NFkB signaling pathways. | No relationship between Nrf2 and CRC |
| Induction of Nrf2-mediated cellular defenses and alteration of phase I activities as mechanisms of chemoprotective effects of coffee in the liver. | Does not discuss CRC |
| Mechanistic insight into beta-carotene-mediated protection against ulcerative colitis-associated local and systemic damage in mice. | No Nrf2 |
| Methylation of the KEAP1 gene promoter region in human colorectal cancer | No Nrf2 |
| Nadroparin sodium activates Nrf2/HO-1 pathway in acetic acid-induced colitis in rats. | No relationship between Nrf2 and CRC |
| NRF-1 is the major transcription factor regulating the expression of the human TOMM34 gene | No Nrf2 |
| Oxidative stress in health and disease: the therapeutic potential of Nrf2 activation. | Does not discuss CRC |
| Proteasome inhibitors MG-132 and bortezomib induce AKR1C1, AKR1C3, AKR1B1, and AKR1B10 in human colon cancer cell lines SW-480 and HT-29. | No relationship between Nrf2 and CRC |
| Red meat and colorectal cancer: Nrf2-dependent antioxidant response contributes to the resistance of preneoplastic colon cells to fecal water of hemoglobin- and beef-fed rats. | No relationship between Nrf2 and CRC |
| Regulation of human carbonyl reductase 3 (CBR3; SDR21C2) expression by Nrf2 in cultured cancer cells. | No relationship between Nrf2 and CRC |
| Somatic mutations of the KEAP1 gene in common solid cancers. | No relationship between Nrf2 and CRC |
| Synergy between sulforaphane and selenium in protection against oxidative damage in colonic CCD841 cells. | No Nrf2 |
| The proteasome inhibitor lactacystin enhances GSH synthesis capacity by increased expression of antioxidant components in an Nrf2-independent, but p38 MAPK-dependent manner in rat colorectal carcinoma cells. | No relationship between Nrf2 and CRC |
| The secretome of colon cancer stem cells contains drug-metabolizing enzymes. | No Nrf2 |
| The sensitivity of cancer cells to pheophorbide a-based photodynamic therapy is enhanced by Nrf2 silencing. | No relationship between Nrf2 and CRC |
| The use of nitric oxide-donating nonsteroidal anti-inflammatory drugs in the chemoprevention of colorectal neoplasia. | No relationship between Nrf2 and CRC |
| Tomato powder impedes the development of azoxymethane-induced colorectal cancer in rats through suppression of COX-2 expression via NF-ÎºB and regulating Nrf2/HO-1 pathway. | No Nrf2 |
| GRP78 knockdown enhances apoptosis via the down-regulation of oxidative stress and Akt pathway after epirubicin treatment in colon cancer DLD-1 cells. | No relationship between Nrf2 and CRC |

S2 Table. Tabulation of included reports.

| **First author** | **Publication date** | **Organism/**  **Cell type** | **Primary results** | **Conclusion** |
| --- | --- | --- | --- | --- |
| [Xu DG](http://www.ncbi.nlm.nih.gov/pubmed/?term=Xu%20DG%5BAuthor%5D&cauthor=true&cauthor_uid=25178491) (24) | 2015 | Cell culture (SW620, HCT116 and SW480) | PYDDT promotes ROS production, increases Nrf2 expression and decreases the CRC risk due to a reduction in genotoxicity and an increment of apoptosis. | Activation of the ROS-JNK signalling pathway triggered by PYDDT induces mitochondrial apoptosis in human colon cancer cells. PYDTT could thus serve as an antitumor agent against CC. |
| [Jung KA](http://www.hindawi.com/59612750/)(32) | 2013 | Cell culture (HT29 and HCT116) | Silenced Keap1 activates Nrf2 signalling in colon cancer cell lines. Silencing Keap1 can upregulate the expression of AKRs and attenuate oxidative damage caused by stress. | AKRs are inducible human genes regulated by Nrf2 in the epithelial cells of the colon. AKR induction is associated with 4HNE detoxification and is highly relevant for carcinogenesis in the human colon. These results support the anti-cancer role of the Nrf2 pathway in colon tissue. |
| [Kang KA](http://europepmc.org/search;jsessionid=2mERN2yZh7jp1WsDlr9D.0?page=1&query=AUTH:%22Kang+KA%22) (43) | 2016 | Cell culture (SNUC5) | Nrf2 expression is higher in those colon cells resistant to 5-FU. Demethylation upregulates Nrf2 in 5-FU resistant SNUC5 cells. | Nrf2 in 5-FU resistant colon cancer cells is upregulated by interacting with DNA methyltransferases and histone demethylases through oxidative stress. |
| [Lu MC](http://www.ncbi.nlm.nih.gov/pubmed/?term=Lu%20MC%5BAuthor%5D&cauthor=true&cauthor_uid=27215610) (33) | 2016 | Cell culture (HepG2 y NCM640) | CPUY192018 provides cytoprotection against oxidative damage caused by DSS in NCM460 cells. Pretreatment with CPUY192018 increases the survival rate by inhibiting apoptosis and by impeding the DSS-induced arrest of the S phase of the cell cycle. CPUY192018 induces an increase in Nrf2 protein levels in colonic NCM460 cells and its accumulation in the nucleus. | Inhibition of the Keap1-Nrf2 interaction is a promising strategy to activate defence mechanisms and may be beneficial for ulcerative colitis. |
| [Yokoo Y](http://www.ncbi.nlm.nih.gov/pubmed/?term=Yokoo%20Y%5BAuthor%5D&cauthor=true&cauthor_uid=26899729) (37) | 2016 | Animal test (mice) | In Nrf2^-/-^ mice treated with KBrO3, atypical hyperplasia, adenoma and adenocarcinoma were all observed in the upper small intestine. These same mice displayed an increase in the number of aberrant crypts compared with their Nrf2^+/+^ counterpart. | The lack of Nrf2 increases the susceptibility of the intestine to carcinogenesis. This involves oxidative stress-induced COX2 overexpression followed by the stimulation of cell cycle progression. This data suggests that individuals with SNPs in the Nrf2 gene have an increased risk of CRC. |
| [Pettersen K](http://www.ncbi.nlm.nih.gov/pubmed/?term=Pettersen%20K%5BAuthor%5D&cauthor=true&cauthor_uid=26585906) (18) | 2016 | Cell culture (SW620 and Caco2) | In SW620 cells, DHA induces an increase in ROS and causes the nuclear import of Nrf2. An increase in the level of Nrf2 was detected in the nucleus of CaCo2 and SW620 cells. | The development of diagnostic tools capable of detecting autophagy and autophagy regulators is necessary to verify that cancer cells lose autophagy and to check their sensitivity to DHA. |
| Ishaq M (44) | 2014 | Cell culture (Caco2, HCT116, SW480 and HT29) | Nrf2 is involved in the protective response of HT29 cancer cells. It induces apoptosis through activating caspase 3/7. | AGP-induced NADPH oxidase generates intracellular ROS. This in turn plays a role in inducing apoptosis in CRC culture cells by activating the ASK1 pathway. In addition, AGP resistant cells develop an efficient, antioxidant Nrf2-mediated system in which Srx protects cells from the toxic effects of anticancer therapy. |
| [Myers JN](http://www.ncbi.nlm.nih.gov/pubmed/?term=Myers%20JN%5BAuthor%5D&cauthor=true&cauthor_uid=25078150) (19) | 2014 | Subjects with Crohn's and UC and Cell culture (NCM460 y SW620) | Nrf2 levels are lower in inflamed tissue of patients with UC and Crohn´s disease. Nrf2 protein levels were twice as high in patients with diverticulitis than in patients with UC-associated cancer. | Hb-α is a mediator of the DNA damage caused by ROS. DNA damage increases the likelihood of CRC. The use of antioxidants or the stimulation of sensitive redox pathways may decrease colitis associated CRC. |
| [Yang Y](http://www.ncbi.nlm.nih.gov/pubmed/?term=Yang%20Y%5BAuthor%5D&cauthor=true&cauthor_uid=24602443)(28) | 2014 | Cell culture (Caco2, HT29, HepG2 and HEK-293) | Low concentrations of digitoflavonoids are potential Nrf2/Are activators in colon tumour, liver and kidney cells. Digitoflavonoids stimulate the expression of antioxidant defence proteins, the expression of Nrf2, and its translocation to the nucleus. After treatment with digitoflavonoids, AKT, ERK1/2 and p38 AMPK phosphorylation increases. Inhibition of AKT and ERK1/2 phosphorylation does not influence Nrf2 activation. p38 phosphorylation however is essential for Nrf2 activation. Mice treated with digitoflavonoids exhibited a decrease in the number and size of tumours and crypts as a result of Nrf2 (and its target genes) activation. | Intestinal digitoflavonoids improve antioxidant power by inducting the expression of the main detoxifying enzymes in a p38- and Nrf2-dependent manner. Digitoflavonoids exert a protective effect and potentially could be used as chemopreventive agents against CRC. |
| [Stachel I](http://www.ncbi.nlm.nih.gov/pubmed/?term=Stachel%20I%5BAuthor%5D&cauthor=true&cauthor_uid=24311782) (35) | 2014 | Cell culture (NCM460) and mice C57BL/6 | IER3 overexpression inhibits Nrf2 activation. The absence of IER3 causes a reduction in ROS levels as a result of an increase in Nrf2 activity. | IER3 balances the crosstalk between the NF-kB and Nrf2 transcription factors. A failure to do so could contribute to oncogenesis initiated by chronic inflammation, such as in the case of colitis-associated CRC. |
| [Sebens S](http://www.ncbi.nlm.nih.gov/pubmed/?term=Sebens%20S%5BAuthor%5D&cauthor=true&cauthor_uid=21990354) (36) | 2011 | Cell culture (NCM460) | Nrf2 is activated when cells are exposed to inflammatory macrophages. This increases proteosomal activity and the expression of proteosomal genes in a Nrf2-dependent manner. | Nrf2 has a high potential to induce the expression of proteosomal genes and to stimulate proteasome activity in untransformed epithelial cells. This is an anti-oxidative response that occurs upon exposure to inflammatory cells. Under these conditions, survival and tumorigenic phenotypes are favoured. An increase in the expression of Nrf2 therefore adds to the oncogenic nature of Nrf2 and its dual role in human disease. |
| [Kim TH](http://www.ncbi.nlm.nih.gov/pubmed/?term=Kim%20TH%5BAuthor%5D&cauthor=true&cauthor_uid=21278237) (6) | 2011 | Cell culture (HCT116 and HT29) | Hypoxia-induced angiogenesis can be blocked by inhibiting Nrf2. Inhibition of Nrf2 reduced tumour growth and decreased angiogenesis in mice xenografts, and was associated with a lower accumulation of HIF-1α under hypoxic conditions. Hypoxia cannot activate HIF-1α in Nrf2-inhibited colon cancer cells. PGC1-α and NRF1 levels were unaffected by inhibition of Nrf2. However, destabilization of HIF-1α is associated with a weakened mitochondrial function in colon cancer cells. | Reduced O_2_ consumption by the mitochondria in Nrf2-inhibited colon cancer cells causes a blockage in NIF-1α-VEGF signalling and a subsequent decrease in angiogenesis and tumour growth. |
| [Saw CL](http://www.ncbi.nlm.nih.gov/pubmed/?term=Saw%20CL%5BAuthor%5D&cauthor=true&cauthor_uid=21261563)L (50) | 2011 | Nrf2 KO and WT mice | Nrf2 KO mice suffer more severe colitis than WT mice after treatment with DSS. After adding AOM, Nrf2 KO mice have a greater tendency to develop CRC and dysplasia, and display increased prolapse and rectal bleeding. Nrf2 is required for protection against inflammation-associated CRC. Nrf2 KO mice have a greater susceptibility of developing aberrant crypts associated with inflammation. | Chemopreventive agents led by Nrf2, block aberrant inflammation and reduce oxidative stress. This not impedes the development of CRC, but can also work against other types of cancer. |
| [Wondrak GT](http://www.ncbi.nlm.nih.gov/pubmed/?term=Wondrak%20GT%5BAuthor%5D&cauthor=true&cauthor_uid=20657484) (30) | 2010 | Cell culture (TH29, HCT116 and FHC) | Cinnamaldehyde upregulates Nrf2 and its downstream target genes. Nrf2 provides protection against oxidative stress-induced genotoxicity. | Cinnamon activates Nrf2 in colon cells and provides chemopreventive benefits against colorectal carcinogenesis. |
| [Li CQ](http://www.ncbi.nlm.nih.gov/pubmed/?term=Li%20CQ%5BAuthor%5D&cauthor=true&cauthor_uid=19706542) (31) | 2009 | Cell culture (HCT116) | Exposing HTC116 cells to NO results in the nuclear translocation of Nrf2 as well as its transcriptional upregulation. | In HTC116 cells, the activation of Nrf2-Keap1 by NO confers resistance to NO toxicity itself. |
| [Osburn WO](http://www.ncbi.nlm.nih.gov/pubmed/?term=Osburn%20WO%5BAuthor%5D&cauthor=true&cauthor_uid=17631644) (48) | 2007 | Nrf2 KO and WT mice | DSS exposure in Nrf2-deficient mice led to the formation of multiple aberrant crypts. In addition, treatment with AOM promoted colitis-associated tumorigenesis (due to inflammation of the colon). | CRC associated with colitis remains a serious health problem. The development of chemoprevention strategies against colitis-associated CRC could be a promising way to prevent it. Given the great influence genotype has on the progression of Nrf2-mediated tumorigenesis and inflammation, the modulation of the Nrf2 pathway could be a useful chemoprevention strategy. |
| [Khor TO](http://www.ncbi.nlm.nih.gov/pubmed/?term=Khor%20TO%5BAuthor%5D&cauthor=true&cauthor_uid=17178849) (49) | 2006 | Nrf2(−/−) mice and WT mice(C57BL/SV129) | Compared with WT mice, Nrf2 KO mice had a smaller average colon length. After exposure to DSS, Nrf2 KO mice showed a loss in crypts with severe inflammation. | Mice deficient Nrf2 showed an increased susceptibility to DSS-induced colitis, possibly due to the reduced expression of antioxidant/phase II detoxifying enzymes, and the increased concomitant expression of proinflammatory mediators. This is the first study that analyses the role of Nrf2 in DSS-induced colitis. This study also supports the rationale that Nrf2 could be an important target for developing chemoprevention strategies, particularly for the prevention of CRC. |
| Bat-Chen W (11) | 2013 | Cell culture (Caco-2 and HT-29) | Allicin induces apoptosis in colon cancer cells and stimulates Nrf2 nuclear accumulation. | Allicin directly promotes cytotoxic effects in human colon cancer cells by inducing apoptosis through Nrf2-associated mechanisms. |
| Pandurangan AK (12) | 2014 | Cell culture (HT-29) | Nrf2 transactivation. | Luteolin controls colon cancer in many ways via the Nrf2 pathway. |
| Xi M (20) | 2013 | Cell culture (HepG2 and HCT116) | Nrf2 has an inhibitory effect on the development of the CRC. Nrf2 diminishes the development of dysplasia. Nrf2 is overexpressed in tumour tissues. | The expression of Nrf2 in standard conditions significantly reduces the development of colon cancer associated with inflammation. |
| Li W (46) | 2008 | Nrf2 KO and WT mice | Compared with WT mice, Nrf2 KO mice suffer from more severe ulcerative colitis, loss of crypts, an infiltration of inflammatory cells, and rectal bleeding. | Nrf2 protects against colonic inflammation and carcinogenesis. |
| Tan BL (25) | 2015 | Mice | Brewer's rice promotes Nrf2 activation. | Brewer's rice decreases oxidative stress and colon carcinogenesis. |
| Kim H (13) | 2013 | Cell culture (HCT116) | Catechol residues are essential for activating Nrf2. | CAPE reduces colonic inflammation. |
| Hu T (41) | 2013 | 76 patients with CRC | Nrf2 is higher expressed in tumour tissue than in normal tissue. Nrf2 overexpression is related to larger tumours with advanced stage and metastasis. Directional metastases may be associated with activation of Nrf2. | CXCR4 and Nrf2 play a very important role in the development of CRC. |
| Kruse ML (27) | 2016 | Cell culture (NCM60) | IMC-co-cultured NCM460 or Colo320 cancer cells were less sensitive to TRAIL/etoposide-induced apoptosis due to Nrf2-induced proteasome activity. Immunostaining of IBD tissues confirmed Nrf2 activation within inflamed areas of the colonic epithelium, and greater proteasome protein expression. | The adaptation of colon epithelial cells to the oxidative stress caused by inflammatory cells is dependent on Nrf2. This process involves an increase in proteasome activity and a resistance to apoptosis. This on one hand protects against colitis-induced tissue damage, but may also favour carcinogenesis. |
| Ji L (42) | 2014 | Metastatic CRC patients | Nrf2 expression is higher in CRC tissue, and is positively correlated with Duke's stage and clinical prognosis. | Nrf2 expression increases in CRC tissue. |
| Pandurangan AK (14) | 2015 | BALB/c mice | Cocoa actives Nrf2 expression. | Cocoa is a chemopreventive agent in colorectal carcinogenesis. |
| Kang KA (38) | 2014 | Cell culture (SNUC5) | Nrf2 promotes resistance to 5-FU treatment in CRC cells. | Nrf2 is proposed as a potential therapeutic target to prevent 5-FU resistance in CRC. |
| Chang LC (52) | 2013 | Subjects with a preoperative diagnosis of CRC | Proteins of the Nrf2 pathway are actively expressed in CRC. | Differences in the expression profiles of Nrf2 pathway proteins between tumour and normal tissues may offer a new direction for CRC treatment. |
| Arlt A (45) | 2009 | Cell culture (LoVo, Colo320 and NCM460) | There is a high activity of Nrf2 in the nucleus of CRC cells. Nrf2 overexpression increases proteasomal activity. | Increases Nrf2 activity in CRC cells promotes proteasome activity and contributes to tumorigenesis. While in early stages Nrf2 is beneficial, in the later stages it contributes to the genesis of CRC. |
| Volonte D (26) | 2013 | Cell culture (HCT116) | Calveolin inhibits Nrf2 translocation to the nucleus, thereby promoting premature senescence of CRC cells. | Additional work is necessary to fully understand the mechanisms by which the caveolin-Nrf2 balance is regulated. It still needs to be determined whether therapeutic interventions aimed at altering caveolin-1/Nrf2 signalling can enhance tumour suppression without accelerating aging and/or age-related phenotypes, or whether they can slow the aging process without necessarily promoting tumour initiation and/or progression. |
| Li Y (15) | 2016 | C57BL/6 mice | Nrf2 is activated by luteolin. | Luteolin protects against CC via the Nrf2 pathway. |
| Pandurangan AK (29) | 2014 | Balb/c mice | Nrf2 is activated by luteolin | Luteolin actives Nrf2 target genes and protects against the progression of CRC. |
| Trivedi PP (23) | 2014 | Swiss Albino mice | MEL increases the expression of Nrf2 and other target genes. | Targeting autophagy and Nrf2 signalling pathways may offer new insights into cancer biology and therapeutic interventions for the treatment of CACC. |
| Cheung KL (51) | 2012 | C57BL/6J male mice | Nrf2 KO mice have an increased number and size of polyps and cell proliferation. | The absence of Nrf2 promotes a hyperproliferation of crypts and an expansion in the size and number of polyps in the colon. |
| Zhao XQ (39) | 2015 | Cell culture (SNU‑C5 CRC and SNU‑C5R) | Nrf2 expression is higher in CRC cells that are resistant to 5-FU treatment. | Epigenetic modifications of Nrf2 could help in blocking resistance to 5-FU. |
| Chiou YS (16) | 2011 | BALB/c mice | PS and RS promote Nrf2 activation and reduce colon tumorigenesis. | PS could be more powerful than RS in mediating the anti-tumorigenic effect of Nrf2 activation. |
| Trivedi PP (17) | 2013 | Swiss mice | Lipoic acid (LA) increases Nrf2 and HO-1 expression in the colon. LA protects against DNA damage in the colon. | LA is a potential agent to ameliorate the severity of UC and its associated systemic damage. |
| Park EJ (21) | 2010 | Cell culture (HT29, A549, and HCT116) | Rottlerin induces Nrf2 nuclear translocation. | Rottlerin induces HO-1 gene expression in HT29 cells. This effect is mediated by the generation of ROS, the activation of p38 MAPK and ERK, and the nuclear translocation of Nrf2. |
| Jang HJ (22) | 2016 | Cell culture (HT-29) | Simvastatin induces Nrf2 expression and its nuclear translocation. Simvastatin induces Nrf2-related antioxidant expression through the ERK and PI3K/Akt pathways. | Simvastatin activates Nrf2 and promotes its translocation into the nucleus. Subsequently the expression of HO-1 related antioxidants is induced via the ERK and PI3K/Akt pathways in HT-29 cells. Further studies are needed to explore the exact role of these simvastatin-stimulated antioxidants. Simvastatin suppresses cell proliferation and increases apoptosis in colon cancer cells. |
